# Dysregulated cellular redox status during hyperammonemia causes mitochondrial dysfunction and senescence by inhibiting sirtuin‐mediated deacetylation

**DOI:** 10.1111/acel.13852

**Published:** 2023-04-26

**Authors:** Saurabh Mishra, Nicole Welch, Manikandan Karthikeyan, Annette Bellar, Ryan Musich, Shashi Shekhar Singh, Dongmei Zhang, Jinendiran Sekar, Amy H. Attaway, Aruna Kumar Chelluboyina, Shuhui Wang Lorkowski, Sanjoy Roychowdhury, Ling Li, Belinda Willard, Jonathan D. Smith, Charles L. Hoppel, Vidula Vachharajani, Avinash Kumar, Srinivasan Dasarathy

**Affiliations:** ^1^ Department of Inflammation and Immunity Lerner Research Institute, Cleveland Clinic Cleveland Ohio USA; ^2^ Departments of Gastroenterology and Hepatology Cleveland Clinic Cleveland Ohio USA; ^3^ Proteomics and Metabolomics core Lerner Research Institute, Cleveland Clinic Cleveland Ohio USA; ^4^ Departments of Pulmonary Medicine Cleveland Clinic Cleveland Ohio USA; ^5^ Cardiovascular and Metabolic Sciences Lerner Research Institute, Cleveland Clinic Cleveland Ohio USA; ^6^ Department of Pharmacology Case Western Reserve University School of Medicine Cleveland Ohio USA; ^7^ Critical Care Medicine, Respiratory Institute, Cleveland Clinic Cleveland Ohio USA

**Keywords:** acetylation, human inducible pluripotent stem cells, mitochondria, multiomics, redox, sirtuin, skeletal muscle, systems biology

## Abstract

Perturbed metabolism of ammonia, an endogenous cytotoxin, causes mitochondrial dysfunction, reduced NAD^+^/NADH (redox) ratio, and postmitotic senescence. Sirtuins are NAD^+^‐dependent deacetylases that delay senescence. In multiomics analyses, NAD metabolism and sirtuin pathways are enriched during hyperammonemia. Consistently, NAD^+^‐dependent Sirtuin3 (Sirt3) expression and deacetylase activity were decreased, and protein acetylation was increased in human and murine skeletal muscle/myotubes. Global acetylomics and subcellular fractions from myotubes showed hyperammonemia‐induced hyperacetylation of cellular signaling and mitochondrial proteins. We dissected the mechanisms and consequences of hyperammonemia‐induced NAD metabolism by complementary genetic and chemical approaches. Hyperammonemia inhibited electron transport chain components, specifically complex I that oxidizes NADH to NAD^+^, that resulted in lower redox ratio. Ammonia also caused mitochondrial oxidative dysfunction, lower mitochondrial NAD^+^‐sensor Sirt3, protein hyperacetylation, and postmitotic senescence. Mitochondrial‐targeted *Lactobacillus brevis* NADH oxidase (MitoLbNOX), but not NAD+ precursor nicotinamide riboside, reversed ammonia‐induced oxidative dysfunction, electron transport chain supercomplex disassembly, lower ATP and NAD^+^ content, protein hyperacetylation, Sirt3 dysfunction and postmitotic senescence in myotubes. Even though Sirt3 overexpression reversed ammonia‐induced hyperacetylation, lower redox status or mitochondrial oxidative dysfunction were not reversed. These data show that acetylation is a consequence of, but is not the mechanism of, lower redox status or oxidative dysfunction during hyperammonemia. Targeting NADH oxidation is a potential approach to reverse and potentially prevent ammonia‐induced postmitotic senescence in skeletal muscle. Since dysregulated ammonia metabolism occurs with aging, and NAD^+^ biosynthesis is reduced in sarcopenia, our studies provide a biochemical basis for cellular senescence and have relevance in multiple tissues.

## Graphical abstract

Ammonia is an endogenous cytotoxin that causes post‐mitotic senescence but the molecular mechanisms are poorly understood. Complementary multiomics, molecular, and metabolic analyses across a comprehensive array of preclinical models and human skeletal muscle show that mitochondrial oxidative dysfunction results in impaired oxidation of NADH with redox (NAD+/NADH ratio) imbalance. Consequent impairment in mitochondrial sirtuin 3, an NAD+ dependent deacetylase, results in hyperacetylation of cellular, primarily mitochondrial, proteins. Restoration of redox status, rather than targeting sirtuin 3 or providing NAD+ precursor, reversed cellular dysfunction and post‐mitotic senescence.

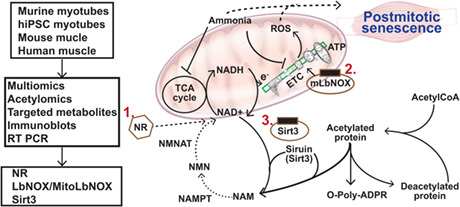

## 1 INTRODUCTION

Hepatic uptake and ureagenesis are the principal physiological mechanisms of disposal of ammonia, a cytotoxic molecule generated during amino acid catabolism, gut bacterial metabolism, and purine breakdown (Dasarathy et al., 2017). Dysregulated ammonia metabolism occurs in a number of chronic diseases including heart, lung, renal and liver disease and in Alzheimer's disease (Adlimoghaddam et al., 2016; Dasarathy & Hatzoglou, 2018; Komatsu et al., 2022). Increased non‐hepatic tissue ammonia, primarily in the skeletal muscle (Ganda & Ruderman, 1976; Mohan et al., 1987; Qiu et al., 2013), is a biochemical mechanism of non‐ureagenic ammonia disposal. Skeletal muscle hyperammonemia causes a number of perturbations including mitochondrial oxidative and electron transport chain (ETC) dysfunction with impaired complex I function, reduced NADH oxidation and consequent lower cellular redox ratio (NAD^+^/NADH) (Davuluri et al., 2016). In addition, lower cellular redox ratio can also be due to decreased NAD^+^ synthesis (Canto & Auwerx, 2011). Even though mitochondrial dysfunction and its consequences during hyperammonemia have been well described(Bai et al., 2001; Davuluri et al., 2016; Kumar et al., 2021), the molecular mechanisms and functional consequences of lower redox ratio are not known.

Cellular redox ratio regulates a number of critical metabolic biosynthesis and oxidation–reduction reactions (e.g. carbohydrate, lipid protein and alcohol metabolism), as well as cellular signaling responses via acetylation of proteins (Canto & Auwerx, 2011). Lysine acetylation is a reversible post‐translational modification that is determined by the relative contributions of acetylases and deacetylases (Choudhary et al., 2014). Sirtuins (Sirt1‐7) are NAD^+^‐dependent class III deacetylases that are responsive to redox ratio and utilize NAD^+^ during deacetylation of lysine and regulate a number of cellular metabolic processes including delayed senescence (Buler et al., 2016; Choudhary et al., 2014). The seven known sirtuins have distinct and yet overlapping subcellular distribution: Sirt1 is localized to the nucleus; Sirt2 is a cytosolic protein that senses cellular redox status to initiate mitochondrial responses; Sirt3, 4 and 5 modulate mitochondrial acetylation status; while Sirt6 and 7 are primarily nuclear proteins involved in histone acetylation and transcriptional regulation (Buler et al., 2016; Choudhary et al., 2014). As a metabolic cofactor, redox determinant, and co‐substrate for sirtuins, NAD^+^ availability is critical for cellular functions (Canto & Auwerx, 2011). Therefore, a number of cellular mechanisms for NAD^+^ generation exist and include de novo synthesis from the essential amino acid tryptophan, a salvage pathway that restores NAD^+^ from nicotinamide generated during sirtuin‐mediated deacetylation and the Preiss‐Handler pathway that generates NAD^+^ from nicotinic acid (Canto & Auwerx, 2011; Johnson & Imai, 2018).

We have previously reported that clinically relevant concentrations of ammonia impair mitochondrial oxidation with a lower redox ratio in murine myotubes and skeletal muscle from mice (Davuluri et al., 2016; Kumar et al., 2021; Qiu et al., 2013). In the present studies, we evaluated NAD^+^ generation and utilization pathways during hyperammonemia using data generated by unbiased approaches. The mechanisms and consequences of reduced redox ratio during hyperammonemia were identified by chemical and genetic methods including the use of nicotinamide riboside (NR) an NAD^+^ precursor, (Trammell et al., 2016), overexpression of either *Lactobacillus brevis* NADH oxidase (LbNOX) without/with a mitochondrial localizing sequence (MitoLbNOX) (Titov et al., 2016), or Sirt3 that preferentially localizes to the mitochondria (Parodi‐Rullan et al., 2018). Translational relevance of these observations was demonstrated in human inducible pluripotent stem cell (hiPSC)‐derived myotubes. Our unbiased data analyses showed lower sirtuin signaling and critical components of NAD^+^ synthesis pathways. Consistently, expression and deacetylase activity of Sirt 3 were lower during hyperammonemia. Whole cell acetylome analyses showed more differentially acetylated proteins in hyperammonemia, including a number of mitochondrial ETC proteins. Increasing NADH oxidation by overexpression of MitoLbNOX reversed the molecular, metabolic and functional consequences of hyperammonemia. Overexpression of Sirt3 reversed acetylation and senescence markers but not mitochondrial dysfunction while NR did not reverse the molecular or functional perturbations induced by hyperammonemia. These data provide a mechanistic basis for how adenine dinucleotides and intermediary metabolites regulate mitochondrial function and signaling responses. Our data also show that targeting the primary defect of impaired NADH oxidation to restore cellular NAD^+^ concentrations and redox ratio rather than providing precursors for NAD^+^ reversed postmitotic senescence markers.

## 2 RESULTS

### 2.1 Hyperammonemia causes perturbations in dinucleotide and sirtuin signaling pathways

**FIGURE 1:**
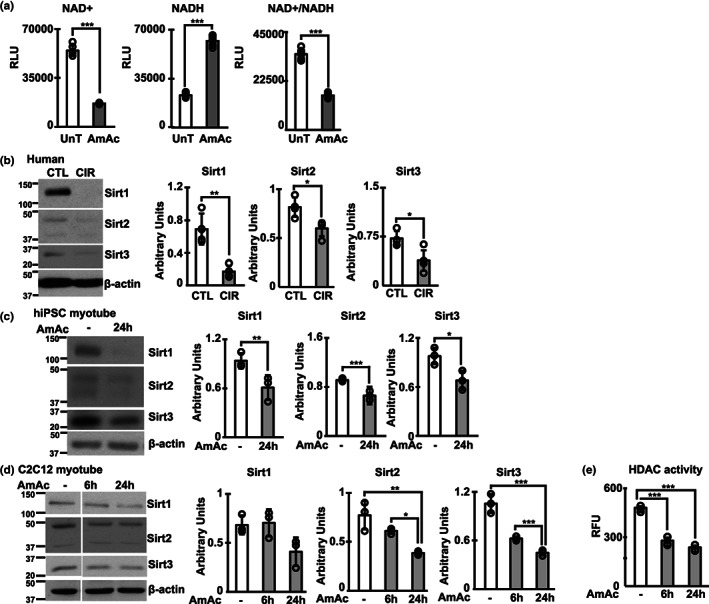
Redox ratio and unbiased approaches show NAD signaling and NAD and sirtuin pathways are perturbed during hyperammonemia. Studies were performed in untreated (UnT) and 10 mM ammonium acetate (AmAc) for 6 or 24 h differentiated murine C2C12 myotubes or human inducible pluripotent stem cell‐derived (hiPSC) myotubes, gastrocnemius muscle from hyperammonemic and control mice, and human skeletal muscle from patients with cirrhosis and controls. (a) Fluorescence‐based detection of NAD^+^, NADH, and the NAD^+^/NADH ratio was performed in myotubes in response to 24 h 10 mM ammonium acetate. NAD^+^/NADH ratio in panel 3 was quantified separately using a different assay and not from the absolute values. (b–d) Representative immunoblots and densitometries of sirtuin (Sirt1, 2, and 3) proteins in myotubes and human skeletal muscle. (e) HDAC activity in murine myotubes. Experiments were performed in biological replicates of *n* = 3 in each group for myotubes. The representative loading controls for panels B and D correspond to the same replicates shown for all proteins probed, respectively; for panel C, representative loading control corresponds to replicates shown for Sirt1 and Sirt3. All data expressed as mean ± SD. *p < 0.05; **p < 0.01; ***p < 0.001 from Student's two tailed *t*‐test for independent samples (panels a–c). Analysis of variance with Tukey post hoc test (panels d and e).

Consistent with previous reports of mitochondrial dysfunction and lower cellular redox ratio (NAD^+^/NADH) during hyperammonemia (Bai et al., 2001; Davuluri et al., 2016; Kumar et al., 2021), we noted lower NAD^+^ and higher NADH concentrations, and lower NAD^+^/NADH ratio (Figure 1a) in differentiated murine myotubes treated with 10 mM ammonium acetate for 24 h (24hAmAc). Consistent with lower NAD^+^, higher NADH, and lower NAD^+^/NADH ratio, our integrated multiomics analyses (Figure S1a–d) identified lower expression of mitochondrial electron transport chain (ETC) complex I components during hyperammonemia across datasets (Figure S2a–c). Differential accessibility of chromatin to NFkB, and transcription of plasma membrane enzymes, *nuclear factor erythroid 2‐related factor 2* (Nfe2l2, also known as Nrf2) and NAD(P)H: quinone oxidoreductase 1 (Nqo1), were increased and are consistent with adaptive responses to restore redox ratio during mitochondrial dysfunction.

**FIGURE 2:**
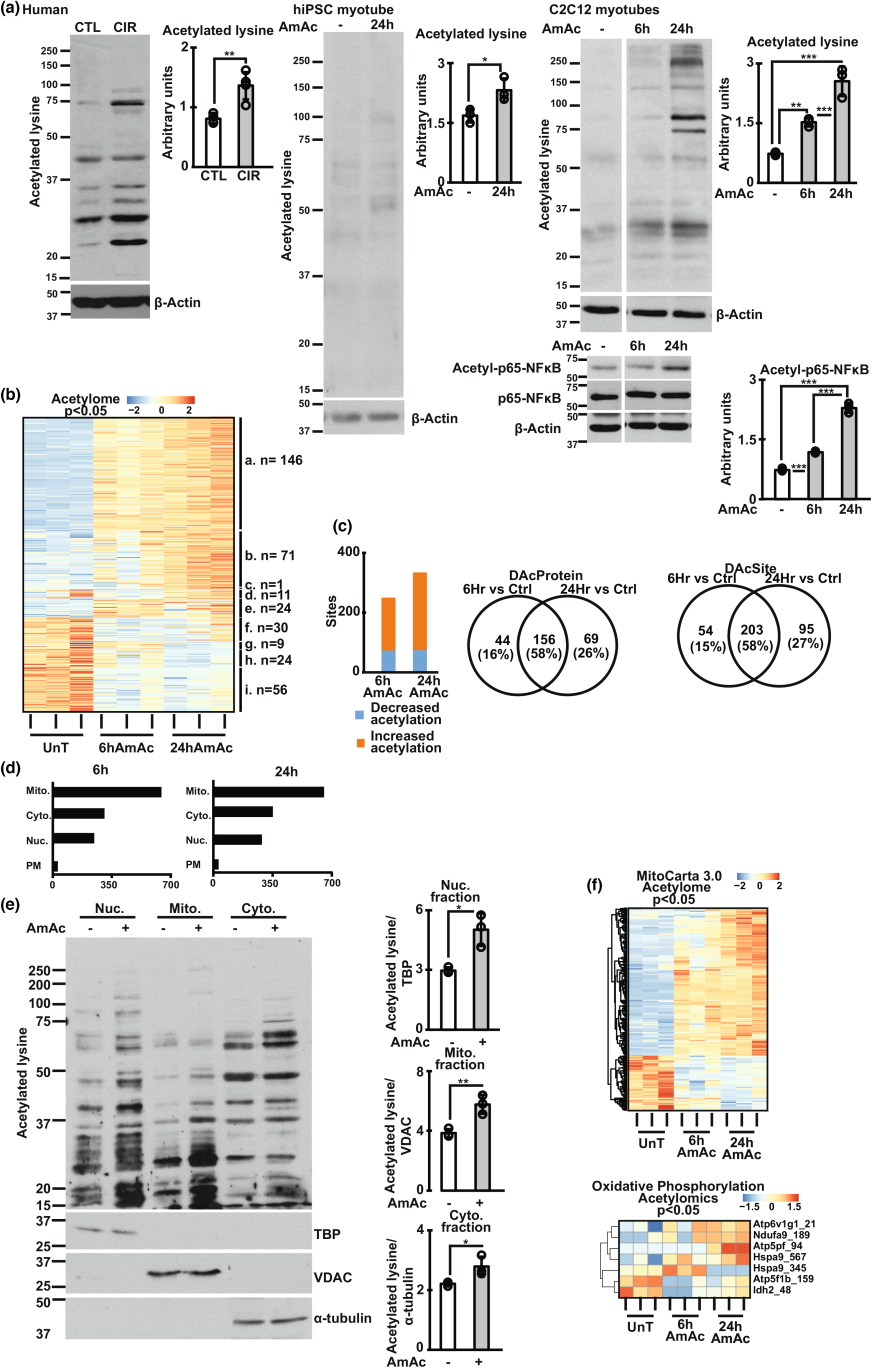
Hyperammonemia increased protein acetylation in myotubes and skeletal muscle. Studies done in C2C12 myotubes or human skeletal myotubes derived from male inducible pluripotent stem cells (hiPSC) treated with 10 mM ammonium acetate for 6 h (6hAmAc) or 24 h (24hAmAc), and vastus lateralis skeletal muscle from human patients with cirrhosis (CIR) and healthy controls (CTL). (a) Representative immunoblots and densitometry of global acetylation of proteins and acetyl‐p65 NFkB. (b) Temporally clustered heatmap of DAP in 6hAmAc, 24 hAmAc and UnT myotubes based on the direction of temporal change: (A, I). *Persistent change* (DAP that have a significant change (increased/decreased in the same direction) in acetylation at both 6hAmAc and 24 hAmAc compared to UnT); (B, H). *Late change* (DAP that do not change at 6hAmAc but have increased/ decreased acetylation at 24 hAmAc compared to UnT); (c). *Early decrease, late increase* (DAP whose expression decrease at 6hAmAc and increases at 24 hAmAc); (d,g). *Pseudosilent change* (DAP that significantly different (increased/decreased) at 6hAmAc compared with 24 hAmAc but neither is different compared with UnT); (E,F). *Early Transient change* (DAP that have a significant change (increase/decrease) in acetylation at 6hAmAc but have no difference in acetylation at 24 hAmAc compared to UnT). (c) Proteins with altered (increased/decreased) acetylation during hyperammonemia. Shared and unique differentially acetylated proteins (DAP) and acetylated lysine sites on DAP (DAS) shown as Venn diagrams. (d) Acetylated proteins in different cellular fractions identified from known datasets (MitoCarta 3.0; Ingenuity Pathway Analysis database). (e) Representative immunoblots and densitometry of global lysine acetylation in cellular fractions. (f) Heatmap of DAS in mitochondrial proteins (mouse MitoCarta 3.0 and oxidative phosphorylation gene sets on Ingenuity Pathway Analysis). All experiments in myotubes performed in *n* = 3 biological replicates and for human muscle studies *n* = 4 in each group. The representative loading controls for all panels with immunoblots correspond to the same replicates shown for all proteins probed. All data expressed as mean ± SD. *p < 0.05; **p < 0.01; ***p < 0.001 from Student's two‐tailed *t*‐test for independent samples for (a, e). Analysis of variance with Tukey post hoc test for A. Statistical significance cutoff (unadjusted *p* < 0.05) for differentially expressed molecules (panels b, c, f).

Given the lower NAD^+^/NADH ratio, we next determined if expression of molecules regulating NAD synthesis in our unbiased datasets were altered during hyperammonemia (Nikiforov et al., 2011) (Figure S3a–c). Transcripts of nicotinamide phosphoribosyl transferase (Nampt) and nicotinamide mononucleotide adenylyl transferase family member 3 (Nmnat3), critical components of the NAD salvage pathway were increased across multiple datasets, suggesting an adaptive response to low NAD^+^ and redox ratio. Expression of nicotinamide riboside kinase 2 (Nmrk2), another critical enzyme in the salvage pathway of NAD^+^ synthesis in the skeletal muscle (Deloux et al., 2018), however, was decreased. Since NAD^+^ biosynthesis mechanisms appeared to be upregulated yet NAD+/NADH ratio was still perturbed with hyperammonemia, we next determined whether downstream utilization of NAD^+^ was altered. We had previously identified NAD^+^ and NAD^+^‐dependent lysine deacetylase (sirtuin) signaling pathway enrichment in the unbiased datasets generated in hyperammonemic murine myotubes and skeletal muscle from mice and humans with cirrhosis (Welch et al., 2021). We generated interaction plots in the sirtuin and NAD signaling pathways (Figure S4a–c). Expression of Protein mono‐ADP‐ribosyltransferase proteins (Parp) 9,12,and 14, that are NAD^+^ consuming enzymes involved in DNA repair(Murata et al., 2019) was higher in the hyperammonemic cellular RNAseq data, though the changes in expression were variable in other models (Figure S3a,b). These data suggest lower sirtuin‐dependent deacetylation in hyperammonemia consumes NAD^+^ and generates Parp for further protein modifications. We next determined whether the individual sirtuins were altered in hyperammonemia.

**FIGURE 3:**
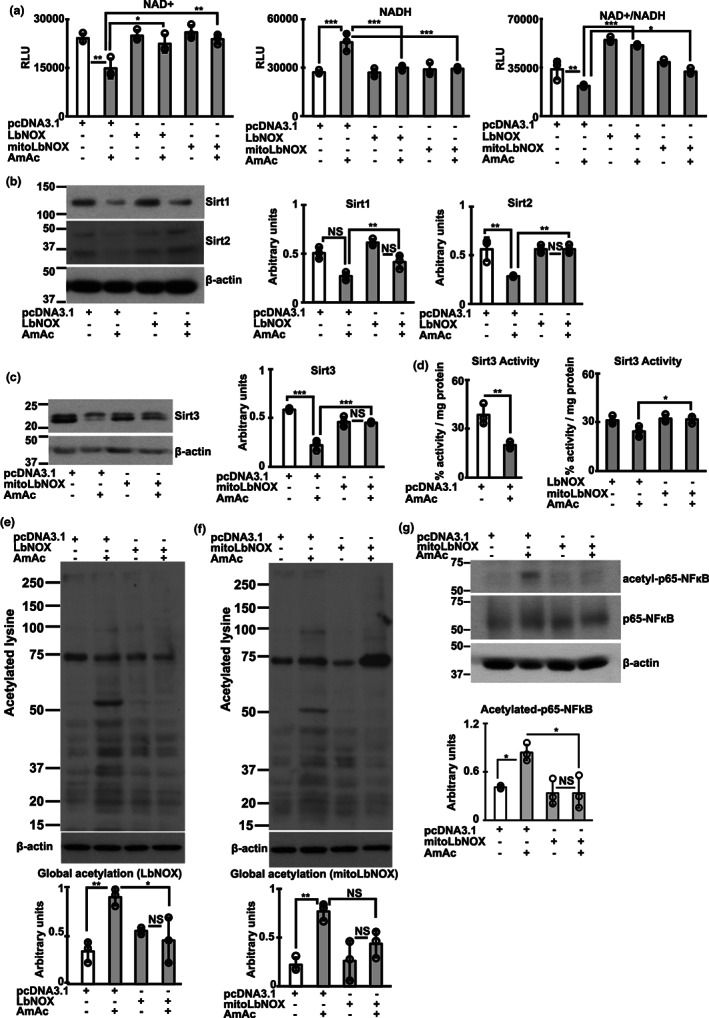
Overexpression of NADH oxidase reverses redox dependent protein acetylation during hyperammonemia. Studies in differentiated C2C12 myotubes that were untreated (UnT) or treated with 10 mM ammonium acetate (AmAc) for 24 h transfected with either empty vector (pcDNA3.1), *Lactobacillus brevis* NADH oxidase without (LbNOX), or with mitochondrial localizing sequence (MitoLbNOX). (a) Fluorescence‐based quantification of NAD^+^, NADH, and the NAD^+^/NADH ratio. NAD^+^/NADH ratio in panel 3 was quantified separately using a different assay and not from the absolute values. (b, c) Representative immunoblots and densitometry of sirtuins (Sirt1, 2, and 3). (d) Sirt3 activity assay in myotubes without/with empty vector (pcDNA 3.1) or transfected with LbNOX/MitoLbNOX with/without 24 hAmAc. (e–g) Representative immunoblots and densitometry of global protein acetylation and acetylation of p65NFkB with LbNOX or MitoLbNOX during hyperammonemia. All experiments in myotubes performed in *n* = 3 biological replicates. All data expressed as mean ± SD. *p < 0.05; **p < 0.01; *** *p* < 0.001 on ANOVA with Tukey post hoc analyses. Experiments with LbNOX and MitoLbNOX were performed at the same time and used the same untreated samples as controls. For Panel C, Sirt3 was probed on the same membrane as Sirt1 and Sirt 2 (Figure S14,B) and the loading control was therefore the same in both panels. The representative loading control for Panel B corresponds to the replicates in the representative blot shown for Sirt1. For all other panels with immunoblots, loading controls correspond to the same replicates shown for all proteins probed.

**FIGURE 4:**
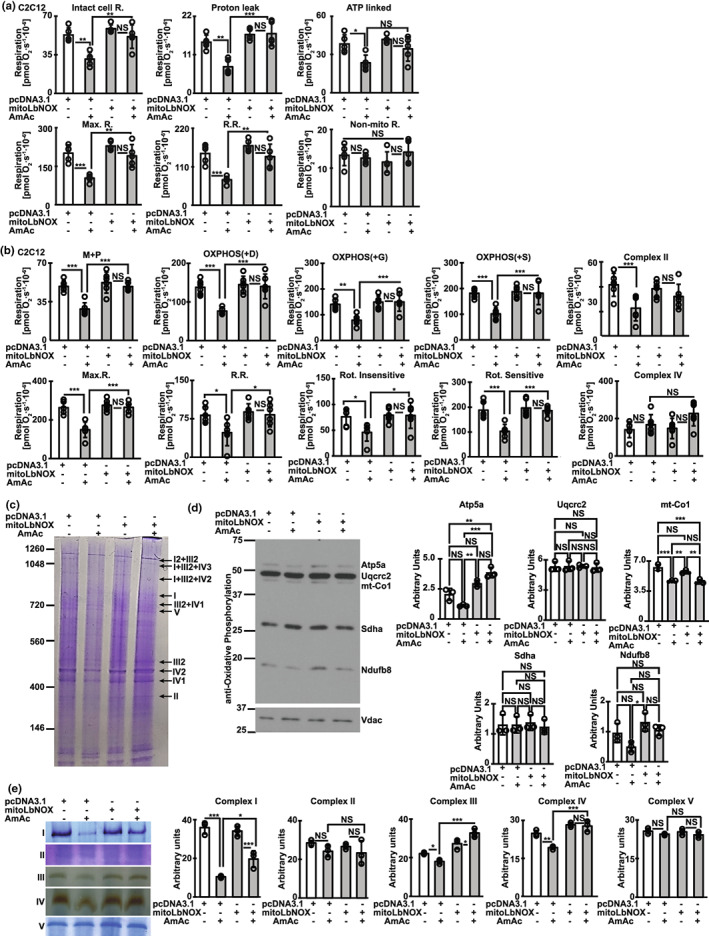
Mitochondrial oxidative defects during hyperammonemia were reversed by restoring redox ratio. Mitochondrial oxygen consumption was measured using high resolution respirofluorometry in differentiated murine C2C12 myotubes that were overexpressed with vector (pcDNA3.1), *Lactobacillus brevis* NADH oxidase without (LbNOX), or with mitochondrial localizing sequence (MitoLbNOX) that were UnT/treated with 24 hAmAc. (**a,** b) Oxygen consumption was measured in intact and digitonin permeabilized myotubes. Studies in intact myotubes included responses to complex I and V inhibitor and uncoupler of oxidative phosphorylation to determine intact cell respiration (Basal R.), proton leak, ATP linked respiration, maximum respiration (Max. R.), reserve respiratory capacity (R.R.) and non‐mitochondrial respiration (Non. Mito.R.). Studies in permeabilized myotubes included proton leak in response to malate (M), pyruvate (P), oxidative phosphorylation (OXPHOS) in response to ADP (D), glutamate (G), and succinate (S), maximum respiration (Max. R.), reserve respiratory capacity (R.R.), rotenone‐sensitive/insensitive respiration and complex II/IV function were measured. (c) Representative images showing mitochondrial supercomplex assembly on blue native gel electrophoresis. (d) Representative immunoblots of components of oxidative phosphorylation and loading control (mitochondrial protein, voltage dependent anion channel‐Vdac). (e) In‐gel activity of electron transport chain (ETC) complexes. Supercomplex assembly and electron transport chain (ETC) component activity and assays were performed in mitochondria isolated from murine myotubes transfected with empty vector/MitoLbNOX C2C12 myotubes treated with/without 24 hAmAc. Oxygraph studies in myotubes were performed in *n* = 5–6 biological replicates and for other studies *n* = 3 biological replicates. Each representative blot in Panel D shows the same four replicates. *p < 0.05; **p < 0.01; ***p < 0.001 compared with respective controls on ANOVA with Tukey post hoc analysis (panels a–d).

### 2.2 Hyperammonemia results in decreased skeletal muscle sirtuin expression and increased protein acetylation

Our experimental and bioinformatics analyses show significant alterations in the NAD and sirtuin pathways during hyperammonemia. Cellular NAD^+^ and NAD^+^/NADH ratio, as well as critical components of NAD^+^ biosynthesis pathways on multiomics data were lower in hyperammonemic models (compared to controls). Consistently, expression of Sirt1‐3 was lower across multiple models of hyperammonemia, including hiPSC‐derived myotubes and skeletal muscle from human patients with cirrhosis. (Figure 1b,c). Similar observations were noted in preclinical models, including differentiated C2C12 murine myotubes, and gastrocnemius muscle from hyperammonemic mice and rats with portacaval anastomosis (PCA) (Figure 1d; Figure S5a,b). Expression of other sirtuin proteins showed variable responses, including a reduction in Sirt5‐7 but no change in Sirt4 in hyperammonemic murine myotubes (Figure S5C). There were no changes in expression of sirtuin mRNA (Figure S5d) in murine myotubes. Decreased expression of sirtuin proteins during hyperammonemia was associated with lower histone deacetylase activity (Figure 1e). Consistently, global muscle protein acetylation was increased in these models (Figure 2a; Figure S5e,f). Lysine acetylation was increased in response to ammonia but not acetate, as demonstrated in myotubes treated with different ammonium salts or equimolar sodium acetate or acetic acid (Figure S5g–i). To determine the functional relevance of increased total protein acetylation, we performed targeted assessment of acetyl‐NFkB p65 expression, an activating post‐translational modification (Chen et al., 2002), because increased transcriptional activity of p65NFkB has been reported during hyperammonemia (Qiu et al., 2013). We observed higher acetyl‐NFkB p65 during hyperammonemia (Figure 2a), which is also consistent with NFkB target gene responses in our unbiased datasets (Figure S6a–g). Based on these protein acetylation studies, we performed quantitative untargeted acetylated protein analyses to identify cellular responses to hyperammonemia.

**FIGURE 5:**
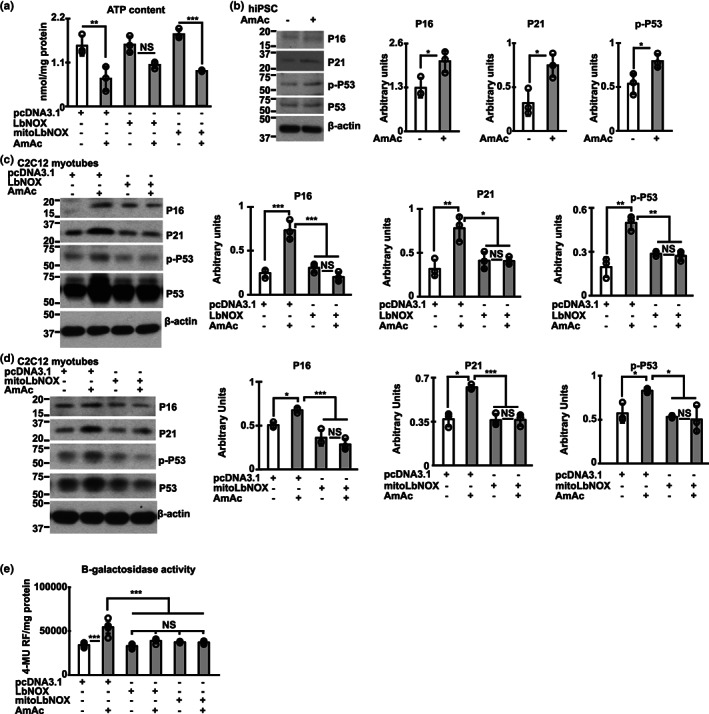
Overexpression of NADH oxidase in myotubes inhibits the hyperammonemia‐induced postmitotic senescence. Studies done in human inducible pluripotent stem cell (hiPSC)‐derived myotubes and murine C2C12 myotubes transfected with *Lactobacillus brevis* NADH oxidase (LbNOX) without/with mitochondrial localizing sequence (MitoLbNOX) treated without (UnT)/ with 10 mM ammonium acetate for 24 h (24hAmAc). (a) ATP content in murine myotubes. (b) Representative immunoblots and densitometry of postmitotic senescence markers (P16, P21, phospho‐P53) in hiPSC‐derived myotubes. (c, d) Representative immunoblots and densitometry of postmitotic senescence markers (P16, P21, phospho‐P53) in LbNOX/MitoLbNOX transfected murine C2C12 myotubes. (e) Senescence associated β‐galactosidase activity in murine myotubes. Studies in differentiated murine C2C12 or hiPSC‐derived myotubes were performed in *n* = 3 biological replicates for all other myotube experiments. The representative loading control shown in panel (b) corresponds to the replicates shown for P21, P‐P53, and P53. For all other panels with immunoblots, representative loading controls correspond to the same replicates shown for all proteins probed. All data expressed as mean ± SD. *p < 0.05; **p < 0.01; ***p < 0.001 compared with respective controls on ANOVA (a, c–e) and Student's two‐tailed *t*‐test for independent samples (b).

**FIGURE 6:**
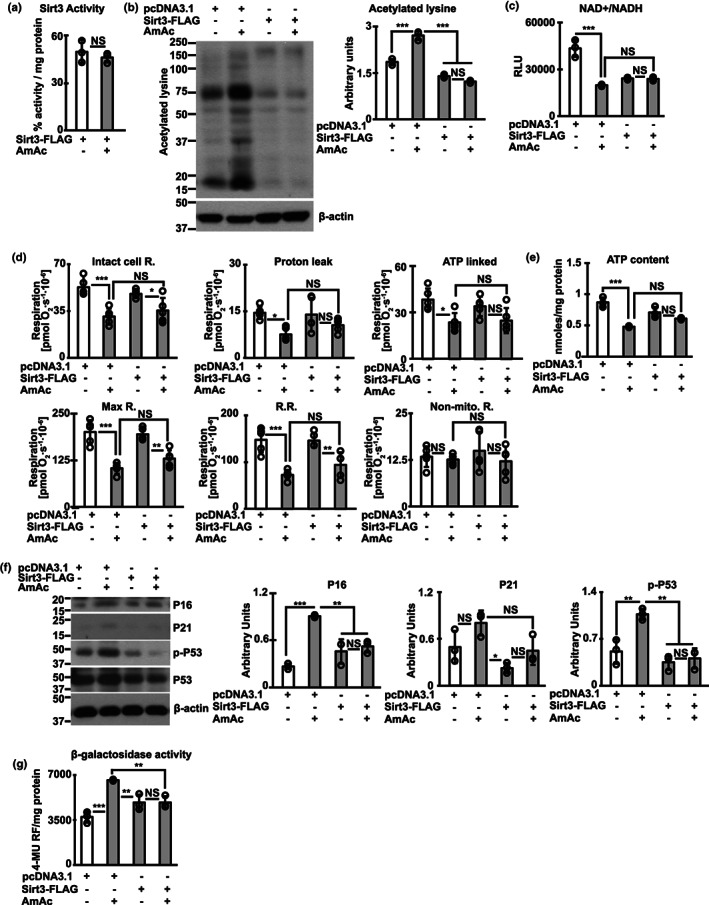
Overexpression of Sirt3 reverses ammonia‐induced protein hyperacetylation in myotubes.Studies done in differentiated murine C2C12 myotubes treated without (UnT)/with 10 mM ammonium acetate for 24 h (24hAmAc) transfected without/with empty vector (pcDNA3.1) or FLAG‐Sirtuin3 construct in pcDNA3.1 (FLAG‐Sirt3). (a) Sirt3 lysine deacetylase activity. (b) Representative immunoblots and densitometry of global protein acetylation. (c) Fluorescence‐based quantification of NAD^+^/NADH ratio. (d) Intact cell respiration measured by high‐resolution respirofluorometry in myotubes with/without overexpression of Sirt3, with/without AmAc compared to pcDNA3.1‐transfected controls. (e) ATP content. (f) Representative immunoblots and densitometries of senescence associated markers P21, P16 and phosphorylated‐P53 (p‐P53). (g) Senescence associated β‐galactosidase activity. Oxygraph studies were performed in *n* = 5–6 biological replicates and all other studies were performed in *n* = 3 biological replicates. For all other panels with immunoblots, representative loading controls correspond to the same replicates shown for all proteins probed.*p < 0.05; **p < 0.01; ***p < 0.001 compared with respective controls on Student's two‐tailed *t*‐test for independent samples (a) and ANOVA with Tukey post hoc analysis (b–g).

### 2.3 Global acetylation landscape in myotubes reveals distinct temporal clustering of molecular responses

We performed an affinity purification‐mass spectrometry of the acetylome in myotubes at 6 and 24 h of hyperammonemia followed by a systems biology approach to analyze and interpret these data. Quality measures including principal component analyses showed clear separation in acetylation patterns between UnT, 6hAmAc and 24 hAmAc in murine myotubes (Figure S7 a,b). Temporal molecular responses to hyperammonemic cellular stress can be clustered based on these patterns as reported earlier (Krishna et al., 2010; Kumar et al., 2021; Welch et al., 2021). Supervised and unsupervised heatmaps showed temporal clustering of differentially acetylated sites (DAS) during 6 and 24 h of hyperammonemia (Figure 2b; Figure S8a–c). Volcano plots of acetylated proteins showed that the 25 differentially acetylated proteins (DAP) in the 6 and 24 h hyperammonemic sets with the lowest *p*‐values showed shared and unique molecules (Figure S8d). Shared DAP that were increased at both 6 and 24 h of hyperammonemia included Eif4b, Map4, Arpc1b, Eif3b, S100a4, Smad4, and Hist1h2bb. Many of these molecules, either directly or indirectly, regulate mitochondrial oxidative function, free radical generation, antioxidant responses, and cell cycle regulation, suggesting their contribution to the molecular phenotype of postmitotic senescence during hyperammonemia as reported earlier (Gorg et al., 2015; Kumar et al., 2021). There were more unique DAP and DAS on proteins at 24 h compared to 6 h of hyperammonemia (Figure 2C; Figure S8e,f), consistent with our finding of lower expression of sirtuin deacetylases with prolonged hyperammonemia in different models. Since different sirtuins have preferential subcellular localization, we performed a bioinformatics based partitioning of the DAP by overlaying the acetylome with known mitochondrial and nuclear, cytosolic and plasma membrane proteins. Our acetylomics data showed that the highest number of DAPs was in the mitochondrial fraction (Figure 2d).

Experimental validation of these bioinformatics studies was performed by quantifying lysine acetylation of cellular compartment proteins in UnT and 24hAmAc‐treated myotubes (Figure 2e). Hyperammonemia causes mitochondrial oxidative dysfunction (Davuluri et al., 2016; Kumar et al., 2021) and others have reported acetylation‐induced defects in ETC components (Ahn et al., 2008). We generated a feature‐extraction heatmap of DAP during hyperammonemia using the mitochondrial and ETC gene sets (Figure 2f). These data, complemented by ETC molecule network analyses, showed that during hyperammonemia, components of the ETC were acetylated (Figure S9a–c). Complementary pairwise correlation studies were used to identify potential regulatory interactions between different proteins based on acetylation sites (Figure S10). Feature analyses of these correlations were followed by STRING protein–protein interaction analyses which showed positive and negative correlations between interacting components of mitochondrial processes, including those of the ETC and the NAD and sirtuin signaling pathways (Figure S11a–f). These findings are consistent with prior reports that the acetylation status of ETC components affects their function (Ahn et al., 2008).

Bioinformatics approaches were used to identify potential preferred amino acid sequences near the acetylated lysine in both the 6hAmAc and 24hAmAc acetylomics data. Unlike the selectivity of kinases, which is determined by linear amino acid sequences or motifs near target phosphorylation sites, target motifs for acetyl transferases have not been as clearly defined, though some motifs have been reported (Dyda et al., 2000; Krtenic et al., 2020). We identified three motifs each in the 6hAmAc and 24hAmAc datasets. A glutamic acid on the immediate N‐terminal (XXXEKXXK) and a leucine (XXXKLXXX) on the immediate C‐terminal were found on a number of acetylated proteins in both the 6hAmAc and 24hAmAc datasets (Figure S11G). These motifs had overlap with known protein domains/functional sites like BH_4 at 6 h of hyperammonemia and CTF/NF‐1 binding domain at both 6 and 24 h of hyperammonemia. Additionally, the KEN box domain identified in our analyses (XXXEKXXK) has been reported to be present on many proteins which regulate cell cycle and consequent postmitotic senescence (Pfleger & Kirschner, 2000). Our findings suggest these motifs may be a site of acetylation regulation in hyperammonemia and could be potential targets for therapeutic interventions. We then determined the mechanisms of increased acetylation and functional consequences during hyperammonemia by strategies that restore cellular NAD^+^ concentrations either by providing a precursor molecule, nicotinamide riboside or increased oxidation of NADH.

### 2.4 Restoration of cellular redox ratio restored sirtuin 3 expression and reverses hyperacetylation

In contrast to the previously reported beneficial effects of NR by others (Canto et al., 2012; Trammell et al., 2016), we did not observe reversal of ammonia‐induced increase in NADH, reduction in NAD^+^ concentrations, and lower NAD^+^/NADH ratio in differentiated murine or hiPSC‐derived myotubes (Figure S12a,b). Low sirtuin (1–3) expression, protein hyperacetylation, and impaired mitochondrial oxidative responses during hyperammonemia were also not altered by NR supplementation (Figure S12c–e). Lack of NR reversal of ammonia‐induced NAD^+^ deficiency or lower redox ratio may be related to the unaltered nicotinamide riboside kinase 1 (NRK1) during hyperammonemia (as seen on our bioinformatics analyses) or context‐dependent responses to NR reported in skeletal muscle by others (Dollerup et al., 2020). We then evaluated if increasing cellular NAD^+^ by oxidizing NADH reversed the redox dependent perturbations.

Overexpression of *Lactobacillus brevis* NADH oxidase (LbNOX) without or with a mitochondrial localizing sequence (MitoLbNOX) to oxidize NADH to NAD^+^, reversed lower redox ratio during hyperammonemia (Figure 3a; Figure S13a,b). These data also show that the decreased ETC complex I activity during hyperammonemia (Davuluri et al., 2016; Kumar et al., 2021) results in lower cellular redox ratio. We determined the functional consequences of restoring cellular redox ratio on the expression of Sirt1‐3. Both LbNOX and MitoLbNOX reversed hyperammonemia‐induced reduction in Sirt1‐3 expression (Figure 3b,c; Figure S13c,d). Interestingly and consistent with the predominantly mitochondrial localization of Sirt3, only MitoLbNOX reversed Sirt3 activity (Figure 3d). Restoration of the expression of Sirt1‐3 by LbNOX/MitoLbNOX was accompanied by reversal of ammonia‐induced protein hyperacetylation in murine myotubes (Figure 3e,f). We also noted reversal of acetyl‐p65NFkB expression only by MitoLbNOX showing the importance of reversing mitochondrial redox in mediating hyperacetylation of certain proteins (Figure 3g; Figure S13e). Interestingly, reversing the redox ratio with either LbNOX or MitoLbNOX restored the expression of NAMPT, NMNAT, enzymes in the NAD salvage pathway enzymes (Figure S13F), showing that these enzymes are redox dependent. Generation of mitochondrial free radicals during hyperammonemia was reversed in response to oxidation of NADH, by MitoLbNOX (Figure S13G). These data extend previous reports that ammonia impairs Complex I function with increased generation of free radicals and oxidative modification of proteins in myotubes and skeletal muscle (Davuluri et al., 2016; Kumar et al., 2021). We then determined if restoring NAD^+^ concentration and redox ratio improved mitochondrial oxidative function.

### 2.5 Hyperammonemia‐induced mitochondrial oxidative dysfunction is reversed by restoration of redox ratio

Consistent with previous reports in murine myotubes and mouse skeletal muscle (Davuluri et al., 2016; Kumar et al., 2021), we noted decreased mitochondrial oxidative function in intact and permeabilized hiPSC‐derived myotubes (Figure S14a–c) which demonstrates the translational relevance of our studies. There were no sex‐based differences in mitochondrial oxidative function in intact hiPSC‐derived myotubes (Figure S14a), consistent with clinical data that muscle loss in patients with cirrhosis who have muscle hyperammonemia is sex‐independent (Welch et al., 2020). Cellular redox ratio regulates mitochondrial oxidative function by regulating NAD^+^ availability for critical biochemical and signaling reactions (Martinez‐Reyes & Chandel, 2020). Mitochondrial oxidative function in differentiated murine myotubes showed that MitoLbNOX reversed hyperammonemia‐induced mitochondrial oxidative dysfunction and ETC responses to targeted substrates and inhibitors (Figure 4a,b). These findings suggest that ETC components and their functional responses to their substrates are redox dependent.

Oxidation of NADH by respiratory complex 1 in the ETC is the first step towards the physiological mechanism of replenishing NAD^+^ from NADH. We and others have reported that ETC components exist as supercomplexes (Jha et al., 2016; Singh et al., 2021) that are disrupted during hyperammonemia (Kumar et al., 2021). Our present studies are consistent with our previous report (Kumar et al., 2021) that hyperammonemia causes ETC supercomplex disassembly and also show decreased expression of components of ETC complexes IV and IV proteins(Figure 4c,d). Recovery of hyperammonemia induced mitochondrial oxidative dysfunction and cellular redox state by MitoLbNOX was accompanied by reversal of ETC supercomplex disassembly and decreased expression of components of ETC (Figure 4c,d). In‐gel activity of ETC complex components also showed reversal of hyperammonemia induced defects (Figure 4e). These studies show that cellular redox regulates mitochondrial oxidative function by altering ETC component assembly and function and provide novel data extending the known redox regulation of enzymes in the TCA cycle (Martinez‐Reyes & Chandel, 2020). Hyperammonemia induced mitochondrial dysfunction was related to acetylation of critical mitochondrial molecules as noted with increased acetylation of mitochondrial biogenesis regulatory molecule, peroxisome proliferator‐activated receptor‐gamma co‐activator (PGC)‐1α as well as critical ETC complex 1, NDUFA9 and their reversal by MitoLbNOX (Figure S15a,b). Hyperammonemia causes mitochondrial oxidative dysfunction and free radical generation that in turn cause postmitotic senescence in myotubes and skeletal muscle (Kumar et al., 2021). We therefore studied if ammonia‐induced skeletal muscle senescence is responsive to redox modulation.

### 2.6 Hyperammonemia induced postmitotic senescence is reversed by redox restoration

Functional consequences of mitochondrial oxidative dysfunction include decreased ATP synthesis and postmitotic senescence (Kudryavtseva et al., 2016; Kumar et al., 2021). Consistent with our findings that MitoLbNOX reversed mitochondrial oxidative dysfunction, ETC complex disassembly and decreased activity by increasing NAD^+^ and redox ratio during hyperammonemia, we identified that ATP content was also restored (Figure 5a). Similar to previous reports of increased senescence markers during hyperammonemia in skeletal muscle in preclinical models (Kumar et al., 2021; Welch et al., 2021) and neural tissue (Gorg et al., 2015; Jo et al., 2021), hiPSC‐derived myotubes also demonstrated similar markers including increased expression of P16, P21 and phosphorylated P53 (Figure 5b) showing the translational relevance of these observations. Interestingly, LbNOX and MitoLbNOX also reversed senescence markers in murine myotubes (Figure 5c–e), which may be related to a combination of restoration of cellular NAD^+^ and redox ratio, mitochondrial oxidative function, and reduction in free radical generation.

Given the potential for sirtuins to reverse senescence, we determined if Sirt3 can directly reverse ammonia induced mitochondrial dysfunction and postmitotic senescence molecular phenotype without altering the redox ratio.

### 2.7 Sirt3 does not restore redox‐dependent mitochondrial dysfunction during hyperammonemia

We overexpressed Sirt3 in differentiated murine myotubes (Figure **S** 15C) and showed that this results in maintained Sirt3 activity during hyperammonemia (Figure 6a). Consistent with the maintained activity with overexpression of Sirt3, ammonia‐induced protein hyperacetylation was reversed (Figure 6b). Interestingly, despite reversal of protein hyperacetylation, decreased NAD^+^ and NAD^+^/NADH ratio was not reversed by Sirt3 overexpression (Figure 6c). Sirt3 overexpression also did not reverse ammonia‐induced mitochondrial free radical generation (Figure S15d), mitochondrial oxidative dysfunction (Figure 6d), or ATP content (Figure 6e) suggesting that Sirt3 mediated deacetylation of hyperacetylated proteins did not reverse ammonia‐induced mitochondrial oxidative dysfunction. Finally, overexpression of Sirt3 reversed ammonia‐induced senescence markers (Figure 6f,g). The discordant responses to Sirt3 overexpression including unaltered ammonia‐induced changes in cellular redox ratio, partial reversal of mitochondrial dysfunction and free radical generation, and reversal of senescence markers suggest that these functional outcomes/responses to sirtuins are not fully coupled in the cellular systems.

Supporting data for all bioinformatics analyses and figures to allow for rigor and reproducibility are shown in Index Table.

## 3 DISCUSSION

In the present studies, we show that hyperammonemia results in impaired ETC function with lower cellular redox ratio, impaired mitochondrial oxidative function, increased free radical generation, and accelerated senescence. Lower oxidation of NADH, due to less expression, activity, and assembly of complex I into the ETC. supercomplex, is the initiating mechanism of lower redox ratio and consequent mitochondrial oxidative dysfunction with cellular senescence (Graphical abstract). Simultaneous defects in NAD biosynthesis and salvage pathways result in lower NAD^+^ concentrations, even with pharmacologic supplementation of NR, an NAD^+^ precursor. As a consequence of lower redox ratio, expression and activity of Sirt3, a mitochondrial predominant NAD^+^ dependent lysine deacetylase, are lower with increased acetylation of cellular proteins, primarily in the mitochondrial compartment. However, Sirt3 overexpression reversed global protein acetylation, but redox ratio was not restored and mitochondrial oxidative dysfunction was only partially reversed. Interestingly, increased mitochondrial NADH oxidation reverses lower redox ratio, mitochondrial oxidative dysfunction, reduced Sirt3 expression, protein hyperacetylation, and postmitotic senescence. Perturbed skeletal muscle ammonia metabolism occurs in a number of chronic diseases and with aging (Dasarathy & Hatzoglou, 2018; Mohan et al., 1987), as well as in neuronal tissue in Alzheimer's disease showing a broad relevance for our studies (Adlimoghaddam et al., 2016; Komatsu et al., 2022; Wiley & Campisi, 2021). These data also provide a biochemical and metabolic mechanistic basis for decreased mitochondrial capacity and lower NAD biosynthesis in sarcopenia of aging (Migliavacca et al., 2019).

Our multiomics analyses were initiated with a layered comparative evaluation of chromosomal access, transcripts and proteins because regulation of components of the ETC, TCA cycle regulatory enzymes, NAD biosynthesis, and sirtuin signaling has been suggested to occur at multiple levels (Buler et al., 2016). There was significant but not complete concordance across layers and models, that may be due to a number of factors that include tissue and context dependent responses (Buler et al., 2016). Lower translation during hyperammonemia (Davuluri et al., 2019; Qiu et al., 2013) and variability between samples in in vivo studies (Kumar et al., 2019) also contribute to discordance across molecular layers. Our multiomics data showed that temporal changes in expression of sirtuins, NAD^+^‐dependent deacetylases, is also a consequence of lower redox ratio. Multiple, novel protein–protein interactions within the broader sirtuin and NAD signaling pathways in our studies showed concordance of critical, differentially expressed molecules during hyperammonemia between interacting molecule. Our network and interactome studies show that expression of components shared between the NAD^+^ and sirtuin signaling pathways, including critical molecules of mitochondrial oxidative phosphorylation, ETC, and the TCA cycle were lower during hyperammonemia. These interactome studies in silico lay the foundation for further complementary studies and direct experimental evaluation of potential regulatory targets at the molecular level during hyperammonemia. Lower sirtuin activity results in increased lysine acetylation of histone and non‐histone proteins in different cellular compartments with consequent alteration in expression and functional perturbations of these modified proteins (Bao & Sack, 2010; Choudhary et al., 2014).

Acetylation is recognized to be among the most prevalent post‐translational modifications in mitochondrial proteins, despite the lack of histones in mitochondrial genome or nucleoids (Bogenhagen, 2012; Choudhary et al., 2014). Our experimental evidence shows lower expression of sirtuins and deacetylase activity during hyperammonemia with increased protein acetylation. Global acetylome analysis showed that acetylation of sirtuins themselves was not altered by hyperammonemia, suggesting that non‐acetylated post‐translational modifications of sirtuins may also play a regulatory role, as has been suggested by others (Flick & Luscher, 2012). Others have reported mitochondrial and non‐mitochondrial non‐histone proteins to be acetylated in heart failure, a condition in which ammonia metabolism is perturbed (Dasarathy & Hatzoglou, 2018; Tsuda et al., 2018). Our complementary acetylomics and subcellular fraction acetylation data, in murine myotubes, suggest that nuclear and mitochondrial proteins are most acetylated during hyperammonemia. Our bioinformatics analyses across multiple molecular layers showed that critical components of the ETC are acetylated and these modifications may contribute to impaired mitochondrial oxidative function because acetylation impairs protein function (Narita et al., 2019). Experimentally, ammonia disrupts the ETC supercomplex with lower expression of complex I component (NDUFB8). Additionally, acetylation of complex I component, NDUFA9, a post translational modification that impairs protein function occurs during hyperammonemia. Restoration of redox ratio improved mitochondrial oxidative function by multiple potential mechanisms including reversal of acetylation of NDUFA9, a component of complex 1, and PGC1α, a regulator of mitochondrial biogenesis as well as supercomplex disassembly, and lower in gel activity of ETC complexes. Reversal of mitochondrial oxidative and ETC. dysfunction was accompanied by lower mitochondrial free radical generation. Thus, ammonia contributes to lower cellular redox ratio by multiple mechanisms that culminate in lower NADH oxidation and free radical generation by targeting complex I in the ETC. Our observations are similar to those reported by others that acetylation decreases complex I function (Ahn et al., 2008; Parodi‐Rullan et al., 2018). Hyperacetylation of mitochondrial proteins during hyperammonemia are consistent with decreased expression and activity of Sirt3, the primary mitochondrial deacetylase. The high acetyl CoA content in mitochondria and a large number of lysine residues in many ETC components have been suggested to contribute to hyperacetylation and oxidative dysfunction (Ahn et al., 2008; Fernandez‐Marcos et al., 2012; Parodi‐Rullan et al., 2018; Tsuda et al., 2018). These data are also similar to previous reports that hyperammonemia impairs mitochondrial oxidative function and ATP synthesis in murine myotubes and mouse muscle tissue (Davuluri et al., 2016; Kumar et al., 2021). In the present studies, we also showed translational relevance of the effects of hyperammonemia in hiPSC‐derived myotubes that had protein hyperacetylation and lower Sirt3 expression with decreased mitochondrial oxidative function and impaired complex I response to substrates. Defects in ETC complex I function results in impaired NADH oxidation with accumulation of NADH and lower NAD^+^ and NAD^+^/NADH ratio(Canto et al., 2015; Davuluri et al., 2016; Kumar et al., 2021; Titov et al., 2016). Of the ETC components, increased acetylation of complex I is consistent with current and previous reports of impaired complex I during hyperammonemia (Davuluri et al., 2016).

In addition to the critical role of adenine dinucleotides for electron transport from multiple oxidation–reduction reactions, NAD^+^ also regulates protein acetylation and provides ADP‐ribose for poly(ADP‐ribose) catalyzed reactions that include regulation of transcription factors, gene expression and DNA repair(Canto et al., 2015; Canto & Auwerx, 2011). Synthesis of NAD^+^ requires precursors and intermediates including tryptophan, nicotinamide, nicotinic acid, nicotinamide riboside and nicotinamide mononucleotide (Canto et al., 2015; Johnson & Imai, 2018). In mammals, the salvage pathway (synthesis of NAD^+^ from nicotinamide regulated by the rate limited enzyme NAMPT) is believed to the major pathway for NAD^+^ biosynthesis (Canto & Auwerx, 2011). Our current studies show that NAMPT and NMNAT, critical enzymes in the salvage pathway, are sensitive to cellular redox status as demonstrated by restoration of their expression with LbNOX. Whether these findings are due to the direct effects of an increase in NAD^+^ and the redox ratio, or, indirectly, via altering metabolite concentrations, is currently unclear. These data suggest alternative strategies to increase NAMPT/NMNAT can promote NAD^+^ salvage and restore metabolic homeostasis. Other approaches include upregulation of NAMPT and inhibiting NAD degradation. Inhibiting NAD utilizing pathways have consequences, including inhibition of sirtuins, PARP, and cluster of differentiation 38 (CD38), which can alter the cellular stress responses. Our integrated bioinformatics analyses of the global landscape of hyperammonemia in skeletal muscle and myotubes shows that critical enzymes of the salvage pathway are differentially altered, suggesting multiple levels of homeostatic regulation of these enzymes. However, experimentally, expression of the critical enzymes NAMPT and NMNAT are both decreased during hyperammonemia and this reduced expression is reversed by restoration of NAD^+^ concentrations, suggesting their redox dependency. Despite increased need for NAD^+^ during hyperammonemia, expression of the sirtuin pathway enzymes (including those in the salvage pathway) on myotube transcriptomics and proteomics is lower at 24 h than at earlier times. These data suggest an adaptive exhaustion of NAD biosynthesis that can be restored by exogenous NADH oxidation.

Interestingly, NR is a pharmacological approach to restore NAD^+^ and redox ratio and support sirtuin mediated deacetylation by generating NAD^+^(Johnson & Imai, 2018; Trammell et al., 2016); however, NR was not effective in reversing the mitochondrial oxidative dysfunction or protein acetylation during hyperammonemia. Our observations are consistent with reports by others that NR does not consistently improve mitochondrial function (Dollerup et al., 2020). Generating NAD^+^ from NR requires NRK1 and ATP (Bieganowski & Brenner, 2004; Johnson & Imai, 2018), both of which are lower during hyperammonemia providing the mechanistic basis of lack of response to NR. Even though we did not directly measure NR uptake, the concentrations and treatment times used in our studies have been shown to result in uptake in skeletal muscle supporting our interpretation that NR metabolism is critical to generate NAD^+^.

Overexpression of Sirt3 alone reversed protein acetylation but did not restore lower mitochondrial oxidative dysfunction and NADH oxidation that may be due to a greater need for NAD^+^ for mitochondrial oxidative function. Compared to genetic approaches to oxidize NADH, Sirt3 overexpression restored only some of the hyperammonemia‐induced perturbations and may be due to inefficient NADH oxidation and consequent lack of restoration of redox ratio. Even though Sirt3 mediated deacetylation is NAD^+^ dependent, reversal of ammonia‐induced hyperacetylation by Sirt3 overexpression with restoration of redox ratio may be due to novel NAD^+^‐independent deacetylation that needs to be evaluated in future. An alternative explanation could be that multiple sirtuins are inhibited during ammonia‐induced redox alteration and the effects of targeting only one of the mitochondrial sirtuins may not be as effective as redox restoration which has the potential to favorably affect multiple sirtuins. These data also suggest that non‐mitochondrial mechanisms are potential contributors of hyperacetylation during hyperammonemia. Even though lower redox ratio and expression of sirtuins can explain increased acetylation during hyperammonemia, protein acetylation levels are also dependent on non‐sirtuin deacetylases, acetyl CoA flux and acetyl‐transferase expression/activity. Multiple metabolic pathways are altered during hyperammonemia on multiomics analyses that could also contribute to our observations of mitochondrial dysfunction (Welch et al., 2021). Our studies lay the foundation for future studies to determine the relative contribution of acetylation and deacetylation to protein hyperacetylation. The limited effects of targeting Sirt3 alone show the high biological relevance of dinucleotide precursors in cellular and tissue function. Importantly, despite deacetylation of mitochondrial proteins, Sirt3 alone was unable to reverse oxidative function suggesting that acetylation is a consequence of the lower redox ratio and not necessarily a mediator of the oxidative dysfunction. However, since acetylation affects different components of the ETC complexes, the impact of acetylation/deacetylation of different components on overall function need to be evaluated in future.

Thus, using genetic and pharmacological approaches, we show that direct oxidation of NADH to restore NAD^+^ and redox ratio reversed lower Sirt3 expression and functional perturbations including protein hyperacetylation and mitochondrial oxidative dysfunction during hyperammonemia. Targeting mitochondrial rather than cytosolic NADH oxidation was consistently more effective in restoring the mitochondrial Sirt3 expression and deacetylase activity. Thus, during hyperammonemia, decreased NADH oxidation with lower redox ratio results in decreased sirtuin expression potentially due to lower salvage pathway flux and consequent hyperacetylation. These perturbations were reversed by restoring mitochondrial ETC Complex I function, providing a novel therapeutic target.

Our studies also showed that ammonia induced postmitotic senescence in myotubes(Kumar et al., 2021) was reversed most effectively and consistently with restoration of redox ratio rather than direct targeting of Sirt3 or providing NR. A number of mechanisms could have contributed to the favorable effects or redox restoration on senescence including lower mitochondrial free radical generation and reversal of acetylation of critical proteins with restoration of mitochondrial homeostasis that delays senescence (Vasileiou et al., 2019). Another potential mechanism for reversal of senescence with restoration of redox ratio may be targeted deacetylation including lower acetylation and consequent reduced activation of p65NFkB, a senescence promoting factor in non‐neoplastic cells (Wang et al., 2009). Even though Sirt3 overexpression did reverse some of the senescence markers, the responses were not as robust as with redox restoration, suggesting that redox status is critical for sirtuin effects on senescence. We also noted that redox restoration lowered free radical generation is another potential mechanism for reversal of cellular senescence. Our multiomics data suggest that DNA damage responses were increased during hyperammonemia. Whether restoring redox status or deacetylation can reverse these responses as the underlying mechanism of reversing senescence needs to be evaluated in future. Our data are consistent with our previous data that lowering ammonia can reverse postmitotic senescence and prevent progressive sarcopenia, whether delaying or preventing cellular senescence can reverse sarcopenia is currently not known.

In summary, our data show that metabolic perturbations of adenine dinucleotide metabolism contribute to mitochondrial oxidative dysfunction, lower cellular redox ratio, and hyperacetylation of regulatory proteins during hyperammonemia. Targeting oxidative dysfunction and NADH oxidation, rather than restoring deacetylation by sirtuin expression, or supplementation with a dinucleotide precursor, results in a consistent restoration of mitochondrial oxidative homeostasis and reversal of postmitotic senescence. The limited effects of targeting Sirt3 alone show the high biological relevance of adenine dinucleotides and mitochondrial redox ratio in maintaining cellular and tissue homeostasis. These data are also of relevance to tissues beyond skeletal muscle given the impact of ammonia on metabolic and mitochondrial dysfunction in multiple organs.

## 4 METHODS

The details of the methods including all cell cultures, experimental techniques including transfections, molecular, biochemical, and functional assays are described in detail in the SUPPLEMENTARY METHODS section.

All human studies were conducted after obtaining a written informed consent (CCF‐IRB 08–546) and animal studies were approved by the CCF Institutional Animal Care Use Committee (2017–1834; 0000–2313).

## AUTHOR CONTRIBUTIONS

Conceptualization, design SM, NW, SR, VV, AK, SD. Methodology SM, NW, RM, AB, AK, VV, SR. SDSoftware, analysis NW, RM, SM. SD. Validation SM, NW, MK, JS, SS, DZ, SL, LL, BW, SD. Formal analyses SM, NW, CH, SD. Investigation SM, NW, RM, AB, SD. Resources JS, GT, SL, SD. Writing original draft all authors. Editing draft all authors. Supervision, project administration SD.

## FUNDING INFORMATION

Funded in part by NIH R01 GM119174; R01 DK113196; P50 AA024333; R01 AA021890; 3U01AA026976‐03S1; U01 AA 026976; R56HL141744;U01 DK061732; 5U01DK062470‐17S2; R21 AR 071046 (SD); K12 HL141952 (AA) and the American College of Gastroenterology Clinical Research Award and NIH K08 AA028794 (NW). RO1 HL156499 (JDS), RO1 AA 028763 (VV). The Fusion Lumos instrument was purchased via an NIH shared instrument grant, 1S10OD023436–01.

## CONFLICT OF INTEREST STATEMENT

None.

## Supporting information


**Data S1:** Supporting Information.

**Data S2:** Supporting Information.

**Data S3:** Supporting Information.

**Figure S1.** Cross‐dataset matching of molecules within cellular, mouse, and human models. (a, b) Heatmaps and (c, d) Venn diagrams showing unique and shared molecules in the sirtuin and NAD signaling pathways from assay for transposase accessible chromatin sequencing (ATACseq), whole cell RNA sequencing (RNAseq) quantitative proteomics data within models of hyperammonemia 10 mM ammonium acetate (AmAc) in differentiated murine C2C12 myotubes treated with AmAc for 3 and 24 h vs. untreated (UnT) controls (*n* = 3 biological replicates for each group; hyperammonemic and control mouse gastrocnemius muscle, *n* = 3–5 in each group) and vastus lateralis muscle from human control subjects or patients with cirrhosis and hyperammonemia (*n* = 4–6 each). All counts were used without restriction to demonstrate expression patterns.
**Figure S2**. Multi‐omic analyses of feature extraction of components the sirtuin signaling pathway. Heatmaps of differentially expressed components of sirtuin signaling pathway. (a) Assay for transposase accessible chromatin sequencing (ATACseq), RNAseq, and proteomics in differentiated murine C2C12 myotubes treated with 10 mM ammonium acetate (AmAc) for 3 or 24 h compared to no treatment (UnT). (b) RNAseq from gastrocnemius muscle from mice treated with AmAc for 28 days compared to those treated with vehicle (phosphate‐buffered saline [PBS]). (c) RNAseq and proteomics from skeletal muscle from humans with cirrhosis (CIR) or healthy controls (CTL). Significance cutoffs for each heatmap (ATACseq *p* < 0.005 with fold change >∣1.5∣; RNAseq in C2C12 myotubes adjusted *p* < 0.05 Benjamini–Hochberg correction; all others unadjusted *p* < 0.05) are as shown in the panels. Experiments were performed in biological replicates of *n* = 3 in each group for myotube experiments; *n* = 4 in PBS and 5 in AmAc‐treated mouse experiments; *n* = 4 in each group for RNAseq and *n* = 6 in each group for proteomics in human skeletal muscle.
**Figure S3**. Multi‐omic analyses of feature extraction from differentially expressed molecules in the NAD signaling pathway. Heatmaps of components of NAD signaling pathway. (a) Assay for transposase accessible chromatin sequencing (ATACseq), RNAseq, and proteomics in differentiated murine C2C12 myotubes treated with 10 mM ammonium acetate (AmAc) for 3 or 24 h compared to no treatment(UnT). (b) RNAseq from gastrocnemius muscle from mice treated with AmAc for 28 days compared to those treated with vehicle (phosphate buffered saline‐PBS). (c) RNAseq from skeletal muscle from humans with cirrhosis (CIR) or healthy controls (CTL). Significance cutoffs for each heatmap (ATACseq) *p* < 0.005 with fold change >∣1.5∣; RNAseq in C2C12 myotubes adjusted *p* < 0.05 Benjamini–Hochberg correction; all others unadjusted *p* < 0.05 are as shown in the panels. Experiments were performed in biological replicates of *n* = 3 in each group for myotube experiments; *n* = 4 in PBS and 5 in AmAc‐treated mouse experiments; *n* = 4 in each group for RNAseq and *n* = 6 in each group for proteomics in human skeletal muscle.
**Figure S4**. Protein–protein interactions within the NAD and sirtuin signaling pathways in unbiased data from hyperammonemic myotubes and skeletal muscle. Circle plots showing protein–protein interactions of significantly differentially expressed components in the sirtuin and NAD signaling pathways. (a) Assay for transposase accessible chromatin sequencing (ATACseq) in differentiated murine C2C12 myotubes (3 and 24 h hyperammonemic compared to untreated [UnT] controls). (b) Whole cell RNA sequencing in differentiated murine myotubes comparing 24 h hyperammonemia with UnT, skeletal muscle from hyperammonemic and vehicle treated mice and human control subjects and patients with cirrhosis. (c) Whole cell proteomics from differentiated murine myotubes (3 and 24 h hyperammonemic compared to UnT) and skeletal muscle from human control subjects and patients with cirrhosis. All data from *n* = 3 in each group for murine C2C12 myotubes; *n* = 6 in each group of mice; and *n* = 5 each for skeletal muscle from patients with cirrhosis and controls (color code: purple = decreased in both connected molecules, orange = increased expression in both connected molecules, gray = different expression direction for both molecules, e.g. decreased in one, increased in the interaction partner). Significance levels for ATACseq was *p* < 0.005 and fold change >|1.5|; whole cell RNA sequencing *p* adjusted with Benjamini–Hochberg correction <0.05; mouse and human muscle tissue RNA sequencing, whole cell, mouse and human muscle tissue proteomics, *p* < 0.05.
**Figure S5**. Hyperammonemia inhibits sirtuins and causes protein acetylation. Representative immunoblots and densitometry of sirtuins in gastrocnemius muscle from hyperammonemic and vehicle treated mice portocaval anastomosis (PCA) rat and sham‐operated (Sham) rat and differentiated murine C2C12 myotubes untreated or treated for 6 and 24 h of 10 mM ammonium acetate (AmAc). (a, b) Sirtuins 1–3 in mice and rats. (C) Sirtuins 4–7 in myotubes. (d) Fold change of expression of sirtuins 1–7 mRNA by real time polymerase chain reaction in differentiated myotubes. (e–i) Representative immunoblots and densitometry of global acetylation of protein from gastrocnemius muscle from hyperammonemic or vehicle treated mice, portocaval anastomosis (PCA) rat and sham‐operated (Sham) rat, differentiated C2C12 myotubes that are untreated (CTL) or treated with sodium acetate (10 mM) and acetic acid (10 mM) or different ammonium salts (10 mM) for 24 h. Data obtained from three biological replicates for myotubes; *n* = 6 in each group for mice and rats. The representative loading control shown in (a) corresponds to replicates shown for Sirt3 and in (b), the representative loading control corresponds to replicates shown for Sirt2 and Sirt3. For all other panels with immunoblots, representative loading controls correspond to the same replicates shown for all proteins probed per panel. Data expressed as mean ± SD. *p < 0.05; **p < 0.01; ***p < 0.001 on Student’s *t*‐test (a, b, d–h) and ANOVA with Tukey post hoc analysis (c, i).
**Figure S6**. Differential expression of p65NFkB targets during hyperammonemia. Heat maps of differentially expressed components of p65NFkB targets. (a–d) Differentiated murine C2C12 myotubes were treated with and without (UnT) 6 and 24 h of 10 mM ammonium acetate (AmAc) on assay for transposase accessible chromatin sequencing (ATACseq), RNAseq, quantitative proteomics, and quantitative acetylomics. (e) RNAseq from mouse gastrocnemius muscle from AmAc‐treated and control mice treated with phosphate‐buffered saline (PBS). RNAseq. (f, g) RNAseq and quantitative proteomics in skeletal muscle from human patients with cirrhosis and control patients. Significance cutoffs used were *p* < 0.05 for myotube proteomics, mouse RNAseq, and human RNAseq and proteomics; *p* < 0.005 for myotube ATACseq; and false discovery rate <0.05 for myotube RNAseq.
**Figure S7**. Quality control measures of whole cell protein acetylomics during hyperammonemia. Global protein acetylome was performed in differentiated murine C2C12 myotubes that were untreated (UnT) or treated with 10 mM ammonium acetate (AmAc) for 6 or 24 h. (a) Principal component analysis (PCA). (b) Mean‐average (MA) plots.
**Figure S8**. Clustering of acetylation sites and proteins in myotubes. Temporal course of differentially acetylated sites on (DAS) proteins and differentially acetylated proteins (DAP) in differentiated C2C12 myotubes treated with 10 mM ammonium acetate (AmAc) for 6 or 24 h compared with untreated (UnT) cells was plotted to identify clustering of molecules. (a) Graphical strategy of supervised temporal clustering. (b) Unsupervised hierarchically clustered heatmaps of DAS during hyperammonemia (6hAmAc or 24hAmAc). (c) Supervised heatmap of unchanged acetylation sites at all 3 timepoints (compared to each other). (d) Volcano plots of differentially acetylated proteins (DAP) of UnT versus. Six or 24 h AmAc treated myotubes with the 25 most significantly different molecules being labeled. All molecules were chosen with no restrictions to identify expression patterns. (e, f) Venn diagrams showing shared and unique DAP and differentially acetylated sites that are increased or decreased during 6 and 24 h AmAc compared with UnT myotubes. Differential expression was defined as *p* < 0.05. To avoid a “divide by zero” error during computation of differential expression, a value of +1 was added to all quantified read counts. All data are from three biological replicates.
**Figure S9**. Oxidative phosphorylation pathway components during hyperammonemia. Differentially acetylated electron transport chain (ETC) subcomponents in differentiated murine C2C12 myotubes that were untreated or treated with 10 mM ammonium acetate (AmAc) for 6 or 24 h. (a) 6hAm or (b) 24hAmAc (vs. UnT) and (c) 24hAmAc versus 6hAmAc from whole cell acetylome. All cellular experiments were done in *n* = 3 biological replicates. *p*‐value cutoff for differentially expressed proteins was set at *p* < 0.05 using an unpaired Student’s *t*‐test. Green = decreased expression, red = increased expression. Significance cutoff for all pathways was set at log (*p*‐value) ≥1.3 by a right‐tailed Fisher’s exact test.
**Figure S10**. Correlation plots of differentially expressed acetylated sites. Global associations of differentially acetylated sites on proteins were evaluated for potential interactions. Correlation plots of differentially acetylated molecules in the sirtuin and NAD signaling pathways. Correlations performed using Pearson’s’ correlation coefficient with a significance level set at *p* < 0.05. Color code of correlation coefficients shown in scale. Differential expression was defined by a *p* value <0.05. Raw data provided in Table S10. Data obtained from three biological replicates each.
**Figure S11**. Correlation plot subsets and protein–protein interactions in the hyperammonemic murine myotube acetylome. (a–c) Correlation subplots from untargeted acetylomics expression levels of differentially acetylated proteins (DAP) in the acetylome in differentiated murine C2C12 myotubes untreated or treated with 6 and 24 h of 10 mM ammonium acetate (AmAc). Tricarboxylic acid (TCA) cycle genes, oxidative phosphorylation genes, and electron transport chain (ETC) genes are shown (blue = positive correlation, red = negative correlation). (d–f) Circle plots showing protein–protein interactions derived from the STRING database in the hyperammonemic myotube acetylome that are shared between gene sets including ETC genes, sirtuin signaling pathway (Sirtuin), and the NAD^+^ signaling pathway (NAD). (g) Motif discovery and known motif analysis in untargeted acetylomics for DAS. Scores shown in each panel indicates best fit with a known motif from the motif database. Experiments were performed in biological replicates of *n* = 3 in each group for myotubes. For correlation matrix of co‐dependent molecules, *r* ^2^ values were used without filter with color codes (red to blue) are shown in the scale adjacent to the figure. Color codes of the gene set labels for the correlation are as indicated. For protein–protein interaction analyses, increased acetylation of both interacting molecules is shown with orange link, decreased acetylation status of both interacting molecules is shown in purple and dissimilar acetylation status of interaction molecules is shown in gray. Significance for differentially acetylated proteins was taken at *p* < 0.05 using a Student’s *t*‐test and functional enrichment was considered significant at –log (*p*‐value) ≥1.3.
**Figure S12**. Myotube responses to nicotinamide riboside. All studies performed in differentiated murine C2C12 myotubes treated with 0.5 or 1 mM nicotinamide riboside (NR) for global acetylation and only 1 mM NR for all other studies 1 h prior to treatment with 10 mM ammonium acetate for 24 h. (a, b) Fluorescence‐based quantification of NAD^+^, NADH, and the NAD^+^/NADH ratios in hiPSC (a) and murine myotubes (b). (v) Representative immunoblots and densitometry of global acetylation of cellular proteins. (d) Representative immunoblots and densitometry of sirtuins 1–3. (e) Oxygen consumption measured in digitonin permeabilized differentiated murine C2C12 myotubes in response to NR alone or NR with ammonium acetate. Studies in permeabilized myotubes included proton leak in response to malate (M), pyruvate (P), oxidative phosphorylation (OXPHOS) in response to ADP (D), glutamate (G), and succinate (S), maximum respiration (Max. R.), reserve respiratory capacity (R.R.), rotenone‐sensitive/insensitive respiration and complex II/IV function were measured. All experiments were performed in three biological replicates. For all panels with immunoblots, representative loading controls correspond to the same replicates shown for all proteins probed per panel. Data expressed as mean ± SD. *p < 0.05; **p < 0.01; ***p < 0.001 on ANOVA with Tukey post hoc analysis (a–d) and Student’s *t*‐test (e).
**Figure S13.** Sirtuin expression in murine myotubes overexpressing LbNOX and mitoLbNOX. (a) Vector map of LbNOX and MitoLbNOX. Representative immunoblots and densitometry of expression of specific molecules in differentiated murine C2C12 myotubes overexpressing FLAG‐LbNOX or FLAG‐MitoLbNOX and treated without/with 10 mM ammonium acetate for 24 h. (b) FLAG. (c) Sirtuin 3 (Sirt3). (d) Sirtuin 1 (Sirt1) and 2 (Sirt2). (e) Acetyl p65NFKB (only with LbNOX). (f) NAD salvage pathway enzymes, nicotinamide phosphoribosyl transferase (NAMPT) and nicotinamide acid mononucleotide adenylyl transferase (NMNAT). (g) Representative flow cytometry data of mitochondrial free radical generation using MitoSOX as the fluoroprobe. Data obtained from three biological replicates each. Data expressed as mean ± SD. The representative loading controls shown in panels (b)–(d) correspond to the same replicates shown for all proteins probed per panel. Panels (c) and (d) representative loading control is the same representative loading control as in Figure 3c because the same membrane was probed for Sirt1, 2, and 3 and the same representative replicates are depicted. In panel (f), the representative loading control shown corresponds to the NMNAT blot. *p < 0.05; **p < 0.01; ***p < 0.001 on ANOVA with Tukey post hoc analysis.
**Figure S14**. Intact cell respiration in human inducible pluripotent stem cells from male‐ and female‐derived myotubes. Mitochondrial oxygen consumption in intact and permeabilized human inducible pluripotent stem cell (hiPSC)‐derived myotubes from male and female subjects treated with 10 mM ammonium acetate for 24 h using a substrate, uncoupler, inhibitor titration protocol. Studies in intact myotubes included responses to complex I and V inhibitor and uncoupler of oxidative phosphorylation to determine intact cell respiration (Basal R.), proton leak, ATP linked respiration, maximum respiration (Max. R.), reserve respiratory capacity (R.R.) and non‐mitochondrial respiration (Non. Mito.R.). Studies in permeabilized myotubes included proton leak in response to malate (M), pyruvate (P), oxidative phosphorylation (OXPHOS) in response to ADP (D), glutamate (G), and succinate (S), maximum respiration (Max. R.), reserve respiratory capacity (R.R.), rotenone‐sensitive/insensitive respiration and complex II/IV function were measured. (a) Intact cell respiration in hiPSC‐derived myotubes from male and female subjects analyzed separately for sex differences. (b) Intact cell respiration in hiPSC‐derived myotubes (male and female combined). (c) Cell respiration in digitonin permeabilized male hiPSC‐derived myotubes. Oxygraph studies in myotubes were performed in *n* = 5–6 biological replicates. *p < 0.05; **p < 0.01; ***p < 0.001 compared with respective controls on ANOVA with Tukey post hoc analysis (a) and Student’s two tailed *t*‐test for independent samples (b, c).
**Figure S15**. Reversal of acetylation of critical mitochondrial proteins by restoring redox ratio. Immunoprecipitate of peroxisome proliferator‐activated receptor‐gamma co‐activator (PGC)‐1α (a) and critical ETC complex 1, NDUFA9 (b) in differentiated murine C2C12 myotubes treated without/with 10 mM ammonium acetate probed with acetyl‐lysine antibody to determine response to MitoLbNOX expression. (c) Representative immunoblot for FLAG tag for Sirt3‐FLAG expression (*n* = 1 in each group for a–c). (d) Flow cytometry measurement of mitochondrial free radical generation with MitoSOX as fluoroprobe The representative loading controls shown in panels (a) and (b) correspond to the same replicates shown for acetyl‐Ndufa9 and acetyl‐PGC1a (*n* = 3; Data expressed as mean ± SD). Student’s two‐tailed *t*‐test for independent samples showed no significant differences.
**Figure S16**. Responses to hyperammonemic human‐inducible pluripotent stem cell‐derived myotubes. (a) Representative photomicrographs and immunoblots of markers of satellite cells (Pax7), myoblasts (MYOD) and myotubes (Myogenin) during sequential induction of human inducible pluripotent stem cell (hiPSC) to form myogenically committed stem cells (satellite cells), myoblasts and differentiated myotubes. (b) Representative photomicrographs of myotube from male and female iPSC treated with 24 h hyperammonemia. Myotube diameter from at least 120 myotubes from each group. ***p < 0.001 on ANOVA with Tukey post hoc analysis (b). The representative loading control shown in (a) corresponds to the same replicates shown for all proteins probed in this panel.

## Data Availability

Dysregulated cellular redox status during hyperammonemia causes mitochondrial dysfunction by inhibiting sirtuin mediated deacetylation. This paper http://www.proteomexchange.org with dataset identifier PXD033430 and 10.6019/PXD033430. Reviewer account details: Username: reviewer_pxd033430@ebi.ac.uk Password: Vhguxruy Mitochondrial responses during hyperammonemia0020ProteomeXChange PRIDE repository http://www.proteomexchange.org with dataset identifier PXD026955 and 10.6019/PXD026955 Integrated molecular landscape perturbations underlie cellular responses during hyperammonemia NCBI Gene Expression Omnibus SuperSeries. GSE171645 https://www.ncbi.nlm.nih.gov/geo/query/acc.cgi?acc=GSE171642 https://www.ncbi.nlm.nih.gov/geo/query/acc.cgi?acc=GSE171643 https://www.ncbi.nlm.nih.gov/geo/query/acc.cgi?acc=GSE171644.
