# Supplementary material for: Dysregulated cellular redox status during hyperammonemia causes mitochondrial dysfunction and senescence by inhibiting sirtuin‐mediated deacetylation
Source: Aging Cell. 2023 Apr 26;22(7):e13852. doi: 10.1111/acel.13852 (PMC10352558; doi:10.1111/acel.13852)
Supplement: Supplementary file 1 — Data S1: Supporting Information. [file ACEL-22-e13852-s003.docx]

**STAR Methods**.

**RESOURCE AVAILABILITY**

***Lead contact***

Further requests for information, reagents and resources should be directed to and will be fulfilled by the lead contact, Srinivasan Dasarathy ([dasaras@ccf.org](mailto:dasaras@ccf.org)).

***Materials availability***

This study did not generate any new reagents.

***Data and code availability***

- The mass spectrometry acetylomics data have been deposited to the ProteomeXchange Consortium via the PRIDE(Perez-Riverol et al., 2022) partner repository with the dataset identifier PXD033430 and 10.6019/PXD033430. Cell and tissue proteomics data during hyperammonemia is available at PRIDE under accession number dataset identifier PXD026955 and 10.6019/PXD026955.

Cell and tissue ATACseq and RNAseq data have been previously reported(Davuluri et al., 2019; Welch et al., 2021) and are available at NCBI Gene Expression Omnibus under accession number GSE171645.

- No new code was written for this paper.
- All other data are contained within the article. Any additional information needed to reanalyze the data reported within the article will be provided by the lead contact upon request.

**EXPERIMENTAL MODEL AND SUBJECT DETAILS**

***Cell lines***

Murine C2C12 myotubes. Differentiated murine C2C12 myotubes were used in cellular experiments as described previously(Qiu et al., 2013; Qiu et al., 2012). In brief, C2C12 myoblasts (ATCC, CRL 1772) were grown at 37°C to confluence in Dulbecco’s modified Eagle’s medium (DMEM) with 10% fetal bovine serum (proliferation medium) to ~80% confluence and differentiated in DMEM with 2% horse serum for 48h. Myotubes were then treated with 10mM of ammonium acetate for 6h and 24h. We have reported these concentrations and times of exposure to reproduce the metabolic, molecular and phenotype changes in human skeletal muscle from human patients and mouse models of hyperammonemia. Cell lysis, protein extraction quantification were performed using protocols standard in our laboratory(Qiu et al., 2013; Qiu et al., 2012). All cellular experiments were performed in at least 3 biological replicates.

Human inducible pluripotent stem cell derived myotubes. Healthy human iPSC from male and female subjects were a generous gift from Jonathan Smith (co-author) were cultured in Cultrex® (R & D Systems) coated plates and grown in mTeSR medium (Stem Cell Technologies, Cambridge, MA) as described previously(Attaway et al., 2023). In brief, cultures were maintained media changes on alternate days until 80% confluence. Cells were dissociated using ACCUTASE™ (Stem Cell Technologies, Cambridge, MA) before passage. Commercial media (AMS Biotechnology; Abingdon, UK) were used for myogenic induction of iPSC to form satellite cells, myoblasts and myotubes in Cultrex® coated plates. Once myoblasts reached ~90% confluence, myotubes were formed after ~3-5 days (**S.Fig.16,A**). Interestingly, there were no differences in male and female derived hiPSC derived myotube diameter and hyperammonemia resulted in a reduction in myotube diameter in both sexes (**S.Fig.16,B**). These methods are similar to our previous studies in murine myotubes with hyperammonemia-induced sarcopenic phenotype(Kumar et al., 2021) and are consistent with no sex based differences in muscle mass in patients with cirrhosis with hyperammonemia(Welch et al., 2020).

***Animal studies***

All procedures involving animals were approved by the Cleveland Clinic Institutional Animal Care and Use Committee (2017-1834; 0000-2313).

Rat muscle. Gastrocnemius muscle from previously reported hyperammonemic portacaval anastomosis (PCA) and sham operated control male Sprague-Dawley rats (Charles River Inc, Danvers, MA) were used to determine acetylation of proteins. The details of these animals and protocol have been previously reported(Kumar et al., 2017). All experiments were done in at least 4 rats in each group.

Mouse muscle. Gastrocnemius muscle from control or hyperammonemic C57/Bl6 male mice 8-10 weeks age were used for acetylation studies. Hyperammonemia was induced by placing an Alzet miniosmotic pump delivering 2.5mmol/kg/d and in control mice, phosphate buffered saline was used in the pumps as reported earlier(Kant et al., 2019). All experiments were done in at least 5 mice in each group.

***Human subjects***

The human studies were approved by the Institutional Review Board at the Cleveland Clinic (CCF 08-546), conformed to the Declaration of Helsinki, and were performed after a written informed consent was obtained from all subjects. Rectus abdominis muscle tissue was obtained from patients with cirrhosis and healthy controls (n=5 each) as previously described. Their clinical details have been previously reported(Qiu et al., 2013).

**METHOD DETAILS**

All chemicals were obtained from Sigma-Aldrich (St. Louis, MO) unless specified. Nicotinamide riboside chloride was obtained from MuseChem (Fairfield, NJ). All antibodies were obtained from Cell Signaling Technology (CST, Danvers, MA).

Overexpression of Lactobacillus brevis NADH oxidase in myotubes. Bacterial, water soluble NADH oxidase from Lactobacillus brevis with a C-terminal Flag-tag without (LbNOX) and with a mitochondrial localizing sequence (MitoLbNOX)(Titov et al., 2016), were subcloned into pcDNA3.1 (Genescript SC1626) and transfected into C2C12 myoblasts, selected using hygromycin (50µg/ml) and differentiated as previously reported(Kant et al., 2019).

Unbiased data analyses. RNA seq and proteomics from whole cells, gastrocnemius muscle from hyperammonemic portacaval anastomosis rat and mice as well as human vasus lateralis were performed using methods described earlier(Kumar et al., 2017; Tsien et al., 2015).

*RNAseq*. In brief, total RNA was extracted, quality determined with an Agilent 2100 bioanalyzer, RNAseq libraries generated, sequenced, and bioinformatics analyses done by Novogene using TopHat2 as a mapping tool as described by us(Davuluri et al., 2019). Gene expression was quantified by HTseq v0.6.1 to count the number of reads mapped to each read and read counts normalized by conversion to fragments per kilobase of transcript per million mapped reads. DESeq 2 R package (.18.0) was used for differential gene expression analyses. Readouts were uploaded to Qiagen’s IPA system for core analyses to classify differentially expressed genes in the sirtuins and NAD pathways.

*Proteomics studies*. Protein from hyperammonemic myotubes and mice were processed for unbiased proteomics on a Thermo Scientific Fusion Lumos mass spectrometry system (Thermo Scientific, San Jose, CA) as previously described using a label free quantification method with the MaxQuant program(Kumar et al., 2019). Pathway analyses were performed as previously described(Kumar et al., 2019; Singh et al., 2021).

*Acetylome analyses*. Whole cell acetylome was analyzed in differentiated C2C12 myotubes. Hyperammonemic and untreated myotubes were lysed in ~500μl urea lysis buffer (20mM HEPES, 9M urea), 1X Halt® protease and phosphatase inhibitor cocktail, pH 8.0. Protein concentrations of the samples were quantified using the bicinchoninic acid assay. Aliquots of 10 mg of the protein extract were subjected to in-solution trypsin digestion. Protein extracts were reduced by dithiothreitol and alkylated by iodoacetamide. Sequencing grade trypsin was added to each sample and incubated at room temperature overnight. Post-digestion, the peptides were purified (desalted) with Sep-Pak® C18 columns (Waters Corporation, WAT 051910) and then lyophilized with a Labconco® Freeze Dryer. Each lyophilized sample was spiked with acetylated Peptide Standard Mixture I containing 8pmol each of 3 acetylated peptides (Protea Biosciences Group, Inc., PS-540-1). Equal amounts of peptide from each sample was lyophilized and enriched using PTMScan® Acetyl-Lysine Motif [Ac-K] Kit (Cell Signaling 13416), and the eluted peptides were dried immediately. Each sample was reconstituted in 30 μl 1% acetic acid, and spiked in 10 μl 50 fmole/ μl Thermo Scientific™ Pierce Peptide Retention Time Calibration Mixture™ (Pierce Biotechnology, Rockford, IL) to a final concentration of 12.5 fmole/μl final concentration as a reference.

A ThermoScientific Fusion Lumos mass spectrometer system was used for these analyses with a Dionex 15 cm x 75 μm id Acclaim Pepmap C18, 2μm, 100 Å reversed- phase capillary chromatography column. Five μl volumes of the extract were injected and the peptides eluted from the column by an acetonitrile/0.1% formic acid gradient at a flow rate of 0.25 μl/min were introduced into the source of the mass spectrometer on-line. The microelectrospray ion source was operated at 1.9 kV. The digest was analyzed using the data dependent multitask capability of the instrument acquiring full scan mass spectra to determine peptide molecular weights and product ion spectra to determine amino acid sequence in successive instrument scans. The data were analyzed by using all CID spectra collected in the experiment to search the mouse SwissProtKB databases(Bairoch & Apweiler, 2000) with the program Sequest bundled into Proteome Discoverer 2.4(Tabb, 2015). The resulting peptides were filtered based on an adjusted FDR of <1% using a decoy database strategy.

Label‐free quantitation (LFQ) for the acetylome was performed using Proteome Discoverer 2.4 to determine acetylated peptide intensities. The LFQ intensities were normalized to total peptide abundance and to compare the relative abundance of these peptides across the hyperammonemic and untreated myotubes.

*Bioinformatics analyses*. Unbiased data included assay for transposase accessible chromatin sequencing (ATACseq), RNA sequencing, and proteomics from untreated (UnT, control) and hyperammonemic differentiated murine myotubes. RNAseq and proteomics data were generated from gastrocnemius muscle from phosphate buffered saline treated and hyperammonemic mice and human skeletal muscle from healthy human subjects and patients with cirrhosis. The global landscape of changes with hyperammonemia have been previously reported(Welch et al., 2021). For the present studies, feature extraction with visualization were performed for the NAD and Sirtuin signaling pathways. Details of our bioinformatics analytical methods have been previously reported(Kumar et al., 2021; Welch et al., 2021). Quality of data was assessed by principal component analysis (PCA) plots, log ratio (M) versus mean average (A) plots. Volcano plots of the acetylomics data were generated by plotting the negative logarithm of the p value on the y axis and log2 ratio on the x axis. To avoid a divide by zero error related to detection of acetylated proteins only in one group, an arbitrary value of +1 was assigned to all data.

*Pathway analyses*. NAD signaling pathway was generated using QIAGEN IPA (<https://digitalinsignts.qiagen.com/IPA>) followed by dimensionality reduction with feature extraction of pathway components to include NAD and sirtuin signaling interactions. Additionally, molecular signatures database (MSigDB, v7.5.1)(Subramanian et al., 2005) were mined for Gene Set Enrichment Analysis (GSEA) collections including hallmark gene sets and curated gene sets including those from WikiPathways(Martens et al., 2021), Kyoto Encyclopedia of Genes and Genomes (KEGG)(Kanehisa & Goto, 2000), GO (XXX), and Reactome(Fabregat et al., 2018) databases. R version 4.1.2 (Bird Hippie) was used for analysis and visualization of features. Circlize v0.4.14(Gu, Gu, Eils, Schlesner, & Brors, 2014) was used for circular visualization of pathway features within relevant pathways and STRINGdb database(Szklarczyk et al., 2021) was used to identify potential protein-protein interactions among the multiomics features that were differentially expressed with hyperammonemia.

*Acetylomics and analyses*. Whole cell acetylome data were analyzed for the most significant representation among the molecules in the selected dataset using the Ingenuity Pathway Knowledge Base (IPKB) using methods described earlier(Welch et al., 2021). Networks of upstream regulators and networks of molecules with known interactions were also generated using the IPKB. Regulatory networks of these unique and shared genes for temporal clusters and datasets were generated and integrated for qualitative comparisons. The upstream regulator analysis tool was used to analyze linkages to RNASeq and whole cell acetylome via coordinated expression levels found within a dataset in order to identify potential upstream regulators and components of canonical pathways. the identification of the different cellular localization of the proteins were done using IPAKB. The identification of acetylation in mitochondrial proteins were performed. Acetylation dataset was compared to senescence and transcription factor databases(Chawla, Tripathi, Thommesen, Laegreid, & Kuiper, 2013; Tacutu et al., 2018; Zhao, Chen, & Qu, 2016) identify the acetylated senescence and transcription factor components and matched against the mouse databases for mitochondria (MitoCarta 3.0)(Rath et al., 2021), and Ingenuity Knowledge Base (Qiagen)(Kramer, Green, Pollard, & Tugendreich, 2014) for nuclear, cytosolic, plasma membrane proteins, and transcription factors, to identify known mitochondrial and non-mitochondrial proteins that were acetylated during hyperammonemia. Motif analysis was performed to determine if specific motifs were more susceptible to acetylation.

*Motif Analysis.* Peptides with a length of 11 residues centered around the differentially acetylated lysine in each protein identified in the acetylomics analysis were accumulated using UniProtID from UniProtKB(UniProt, 2021). Protein motifs in those sequences were identified with MoMo(Cheng, Grant, Noble, & Bailey, 2019) using the motif-x algorithm with a 0.000001 p-value cutoff(Cheng et al., 2019). Significant motifs obtained from MoMo for each treatment comparison were analyzed with TomTom(Gupta, Stamatoyannopoulos, Bailey, & Noble, 2007) to identify whether the significant motifs had similarity to known PROSITE(Sigrist et al., 2013) fixed-length motifs using Pearson correlation coefficient and a significance threshold of E-value <10 (program pre-sets).

*Interaction analyses*. Differentially expressed molecules (ATACseq, RNAseq, proteomics, acetylomics) were fit on known networks to identify patterns of differential expression. The direction of known interactions between molecules were performed using STRING database as described above. Potential novel relationships between acetylated molecules were identified using the Pearson correlation coefficient to remove sample variations that may occur if only gene counts across the acetylome are used.

Immunoblots*.* Immunoblots were performed using protocols described by us earlier(Qiu et al., 2012). Tissue or cellular protein were extracted, their concentrations measured using bicinchoninic acid and separated by polyacrylamide gel electrophoresis, electrotransferred onto PVDF membranes (Bio-Rad, Hercules, CA) and incubated with primary antibody (1:1000 concentration unless specified), washed in Tris-buffered saline with 0.1% Tween 20 (TBST) and incubated with appropriate secondary antibodies. Immunoreactivity was detected with a chemiluminescent horse radish peroxidase substrate (Millipore, Billerica, MA, USA) and densitometry quantification done using Image J(Schneider, Rasband, & Eliceiri, 2012). Blots were cropped from the full blots to be representative of the average densitometry and hence not necessarily from the same run or sample replicate. Only one cropped loading control is shown in each panel that was derived from one of the representative blots shown in the figure. However the loading control used to calculate the arbitrary units (densitometry of protein of interest/densitometry of the loading control) was always from the same sample replicate to ensure that the loading effect was taken into consideration. When the same sample was used for multiple blots, only loading control of that membrane was used.

Immunoprecipitation assays. Immunoprecipitation for PGC1α and ETC complex 1 component, NDUFS9 were performed using protocols previously described(Davuluri et al., 2019).

Fluorometric detection of deacetylase activity. Deacetylase activity in ammonia (10mM) treated C2C12 myotubes was measured by a fluorometric assay (BioVision Inc., Milpitas, CA, USA) using manufacturer protocol. In brief, ammonia treated and untreated C2C12 myotubes were lysed using lysis buffer containing 20mM Tris HCl pH8, 137mM NaCl , 1% Nonidet P-40 (NP-40), 2 mM EDTA and protein content was quantified using BCA assay. 50µg of each cell extract was added to 85µl (final volume) of Milli-Q® water in each well in a 96 well plate. Appropriate positive and negative controls were used per the manufacturer protocol. Then, 10µl of the 10X HDAC Assay Buffer followed by 5µl of the HDAC colorimetric substrate was added to each well. The plate was incubated at 37^0^C for 1 hour and the reaction stopped using 10µl of lysine developer and fluorescence recorded at Ex/Em = 368/442 nm on a Spectramax fluorometer (Molecular Devices, Inc. San Jose, CA).

Sirt3 activity assay*.* Sirt3 activity was determined in ammonia treated C2C12 myotubes for 6h and 24h using SirT3 activity Assay kit (Abcam, Cambridge, MA) per manufacturer protocol. In brief, myotubes were lysed using, non-denaturing lysis buffer (20mM Tris HCl pH 8, 137mM NaCl , 1% Nonidet P-40 (NP-40), 2mM EDTA). 25ug of cell lysate, Sirt3 assay buffer, fluor-substrate peptide, NAD^+^ and developer were added to each well of a 96 well microtiter plate and mixed well. Activity was recorded at fluorescence intensity over 30 to 60 minutes using microtiter plate fluorometer with excitation at 500 nm and emission at 540nm.

Mitochondrial respiration using high resolution respirometry*.* Mitochondrial oxygen consumption *in situ* in intact and permeabilized myotubes quantified using high resolution respirometry at 37°C on a high sensitivity respirometer (Oroboros, Innsbruck, Austria) using protocols described by us(Kumar et al., 2019; Kumar et al., 2021; Singh et al., 2021).

*Intact cell respiration*. Intact cell respiration was measured in non-permeabilized intact murine or hiPSC derived myotubes that were either untreated or treated for 24h with 10mM ammonium acetate. ATP-linked respiration, proton leak, and maximum respiratory capacity were measured in response to oligomycin (complex V inhibitor), the protonophore, carbonyl cyanide-4-(tri fluoromethoxy) phenylhydrazone (FCCP), and the response to complex I inhibitor, rotenone, and Antimycin A, an inhibitor of cytochrome c reductase.

*Substrate inhibitor responses in permeabilized myotubes*. In brief, following measurement of oxygen consumption in intact differentiated C2C12 and hiPSC derived myotubes in mitochondrial respiration medium, MiR05, digitonin was used to permeabilize cells. Oxidation coupled to phosphorylation was quantified in response to substrates for complexes I and II and ADP. Mitochondrial substrates malate, pyruvate, and glutamate were added to measure complex I function. Succinate was then added as a complex II substrate. Maximal oxidative capacity, or maximum respiration, was measured by the addition of FCCP, followed by rotenone to inhibit electron flow across complex I. Antimycin A (complex III inhibitor) was then added to determine nonmitochondrial residual oxygen consumption rate. Uncoupled complex IV oxidation rate was calculated using sodium azide followed by TMPD and ascorbate. Oxygen concentration and flow rates were recorded at 2-second intervals to measure oxygen consumption rates using DatLab2 from Oroboros. Data were generated from at least 6–7 biological replicates after calibration of the oxygen sensors and instrument background corrections. All data were expressed as oxygen consumption in pmol/s normalized to cell number allow for comparisons across experiments as validated by us(Kumar et al., 2019).

ATP content. Total cellular ATP content in myotubes was quantified using a bioluminescence assay with a commercial kit (Molecular Probes, Eugene, OR, USA) as previously described(Davuluri et al., 2016; Kumar et al., 2019).

β-galactosidase activity. A fluorometric assay was used to quantify senescence-associated β-galactosidase activity in myotubes using a modification of methods previously described(Kumar et al., 2021). In brief, cells were lysed in buffer (5 mM 3(3-cholamidopropyl) dimethylammonio)-1-propane sulfate, 40mM citric acid, 40mM sodium phosphate, 0.5mM benzamide, 0.25 phenylmethylsulfonyl fluoride), the lysate was vortexed and the supernatant after centrifugation at 12,000g for 5 minutes at 4^0^C was mixed with an equal volume of reaction buffer (40mM citric acid, 40mM sodium phosphate, 300mM sodium chloride, 10mM β-mercaptoethanol, 4mM magnesium chloride, 1.7mM 4-methylumbelliferyl-d-galactopyranoside in water). The reaction was stopped with stop solution (400mM sodium bicarbonate) and fluorescence readings were obtained with an excitation at 360nm and emission at 465nm and data expressed as relative fluorescence units/μg protein.

Mitochondrial supercomplex assembly. Mitochondrial electron transport chain complexes are exist as a supercomplex on the inner mitochondrial membrane(Jha, Wang, & Auwerx, 2016). Supercomplex assembly was evaluated on blue native polyacrylamide gel electrophoresis (BN-PAGE) using methods reported by us(Kumar et al., 2021; Singh et al., 2021). In brief, myotubes were washed in ice cold PBS, trypsinized, collected in proliferation medium, centrifuged at 800g for 2 min at 4°C and resuspended in 1 ml. mitochondrial lysis buffer (0.25M sucrose, 20 mM HEPES-KOH, pH 7.5, 10mM KCl, 1.5mM MgCl2, 1mM EDTA, 1mM EGTA, 0.1mM PMSF) for 20 min. The cell suspension was homogenized and centrifuged at 800g for 10 min at 4°C to remove the nuclear pellet. The supernatant was centrifuged at 10,000g at 4°C, mitochondria isolated, permeabilized with digitonin and centrifuged at 10,000g. The supernatant was subjected to blue native polyacrylamide gel electrophoresis and treated with destaining solution (5:4:1 ratio of water, methanol, acetic acid) to identify mitochondrial supercomplexes.

The supernatant after mitochondrial isolation was mixed with acetone in a 1:3 ratio and incubated at -20^0^C overnight and centrifuged at 1500g for 10 min at 4^0^C to obtain the cytosolic pellet.

Mitochondrial complex activity*.* In gel activity of individual ETC complexes was performed as reported earlier(Kumar et al., 2021). In brief, a high resolution BN-PAGE was performed in mitochondria isolated as described above from untreated or hyperammonemic myotubes. Gels were incubated with substrates for each ETC complex. Reactions were stopped by washing the gels with deionized water and treated with10% acetic acid for colorimetric quantification.

Flow cytometric detection of mitochondrial free radicals. Mitochondrial free radicals were quantified using flow cytometry analyses as previously reported(Kumar et al., 2019). In brief, 10,000 events were collected for each sample after gating out debris. Sample data were collected utilizing a BD FACS flow cytometer. Data files were analyzed using FlowJo V10. Prior to analysis, C2C12 myotubes transfected with pcDNA3.1, LbNOX, MitoLbNOX, and Sirt3 were incubated without/with 10mM ammonium acetate for 24 h at 37 °C and 5% CO_2_. Mitochondrial free radicals were detected using MitoSOX Red superoxide indicator (Invitrogen, Carlsbad, CA) were added to cells at a final concentration of 5 μM and used per manufacturer instructions. All experiments were performed in three biological replicates for each group.

Adenine dinucleotide quantification*.* Individual dinucleotides (NAD^+^, NADH) were quantified in the cell lysates incubated with 0.4N HCl at 60°C for 15 min. followed by the addition to 0.5M of Trizma base and incubation at room temperature for 10 min. for NAD^+^ assay or with 0.5M Tri-HCl for 5 min. for NADH assay using the GLO-assay kit (Promega). In addition, the NAD^+^/NADH ratio was quantified directly by a separate assay as previously described(Davuluri et al., 2016) using a luciferase based protocol per the manufacturer protocol (GLO-NAD/NADH assay kit, Promega).

RT PCR based quantitative measurement of Sirtuins during hyperammonemia. C2C12 myotubes were treated with 10mM ammonium acetate for 24h and total RNA was isolated using RNeasy kit (QIAGEN, MD, USA) . cDNA was synthesized using SuperScript™ III First-Strand Synthesis System using 3ug of RNA. The cDNAs of the target gene were amplified with specific primers, using SYBR green (BIO-RAD, USA) with β-actin as a control. Primers sequences are as follows: Sirt1 FW 5'-CCTTGGAGACTGCGATGTTA-3', Sirt1 RV 5'-GTGTTGGTGGCAACTCTGAT-3'; Sirt2 FW 5'-GCAGTGTCAGAGCGTGGTAA-3', RV 5'-CTAGTGGTGCCTTGCTGATG-3'; Sirt3 FW 5'-TACAGGCCCAATGTCACTCA-3', RV 5'-ACAGACCGTGCATGTAGCTG-3'; Sirt4 FW 5'-CGCTGCTCAAGATCCCTAAG-3', RV 5'-GCGACACAGCTACTCCATCA-3'; Sirt5 FW 5'-GACTCAAGACGCCAGAATCC-3', RV 5'-CAGAGGATGTTCCCACCACT-3'; Sirt6 FW 5'-CTGGTCTGGAACTCACTGCT-3', RV 5'-CGGGTGTGATTGGTAGAGAG-3'; Sirt7 FW 5'-GGCACTTGGTTGTCTACACG-3', RV 5'-GTGATGCTCATGTGGGTGAG-3'. The expression was quantified in the terms of fold changes over control as described by us earlier(Qiu et al., 2012).

Statistical Analyses. Cellular experiments were performed in biological replicates of n=3 in each group for myotubes. In unbiased analyses, experiments were performed in n=4 in PBS and 5 in AmAc-treated mice; n=4 in each group for RNAseq in each group for proteomics in human skeletal muscle. Differential expression (compared to untread) for ATACseq data was taken at p<0.005 and FC>|1.5| and for C2C12 myotube RNAseq data, p-value<0.05 adjusted for multiple testing with the Benjamini-Hotchberg procedure. All other unbiased data, including acetylomics, differential expression was considered at p<0.05. These different p-values were used in order to identify a similar number of molecules for functional enrichment studies. All data were expressed at mean±SD unless specifically stated. Qualitative variables were compared using the chi square test. Quantitative variables were compared using the Student’s ‘t’ test or analysis of variance with Bonferroni post hoc analysis for multiple groups. Significance for all myotube experiments was set at 5%.

Attaway, A. H., Bellar, A., Mishra, S., Karthikeyan, M., Sekar, J., Welch, N., . . . Dasarathy, S. (2023). Adaptive exhaustion during prolonged intermittent hypoxia causes dysregulated skeletal muscle protein homeostasis. *J Physiol, 601*(3), 567-606. doi:10.1113/JP283700

Bairoch, A., & Apweiler, R. (2000). The SWISS-PROT protein sequence database and its supplement TrEMBL in 2000. *Nucleic Acids Res, 28*(1), 45-48. doi:10.1093/nar/28.1.45

Chawla, K., Tripathi, S., Thommesen, L., Laegreid, A., & Kuiper, M. (2013). TFcheckpoint: a curated compendium of specific DNA-binding RNA polymerase II transcription factors. *Bioinformatics, 29*(19), 2519-2520. doi:10.1093/bioinformatics/btt432

Cheng, A., Grant, C. E., Noble, W. S., & Bailey, T. L. (2019). MoMo: discovery of statistically significant post-translational modification motifs. *Bioinformatics, 35*(16), 2774-2782. doi:10.1093/bioinformatics/bty1058

Davuluri, G., Allawy, A., Thapaliya, S., Rennison, J. H., Singh, D., Kumar, A., . . . Dasarathy, S. (2016). Hyperammonaemia-induced skeletal muscle mitochondrial dysfunction results in cataplerosis and oxidative stress. *J Physiol, 594*(24), 7341-7360. doi:10.1113/JP272796

Davuluri, G., Giusto, M., Chandel, R., Welch, N., Alsabbagh, K., Kant, S., . . . Dasarathy, S. (2019). Impaired Ribosomal Biogenesis by Noncanonical Degradation of beta-Catenin during Hyperammonemia. *Mol Cell Biol, 39*(16). doi:10.1128/MCB.00451-18

Fabregat, A., Jupe, S., Matthews, L., Sidiropoulos, K., Gillespie, M., Garapati, P., . . . D'Eustachio, P. (2018). The Reactome Pathway Knowledgebase. *Nucleic Acids Res, 46*(D1), D649-D655. doi:10.1093/nar/gkx1132

Gu, Z., Gu, L., Eils, R., Schlesner, M., & Brors, B. (2014). circlize Implements and enhances circular visualization in R. *Bioinformatics, 30*(19), 2811-2812. doi:10.1093/bioinformatics/btu393

Gupta, S., Stamatoyannopoulos, J. A., Bailey, T. L., & Noble, W. S. (2007). Quantifying similarity between motifs. *Genome Biol, 8*(2), R24. doi:10.1186/gb-2007-8-2-r24

Jha, P., Wang, X., & Auwerx, J. (2016). Analysis of Mitochondrial Respiratory Chain Supercomplexes Using Blue Native Polyacrylamide Gel Electrophoresis (BN-PAGE). *Curr Protoc Mouse Biol, 6*(1), 1-14. doi:10.1002/9780470942390.mo150182

Kanehisa, M., & Goto, S. (2000). KEGG: kyoto encyclopedia of genes and genomes. *Nucleic Acids Res, 28*(1), 27-30. doi:10.1093/nar/28.1.27

Kant, S., Davuluri, G., Alchirazi, K. A., Welch, N., Heit, C., Kumar, A., . . . Dasarathy, S. (2019). Ethanol sensitizes skeletal muscle to ammonia-induced molecular perturbations. *J Biol Chem, 294*(18), 7231-7244. doi:10.1074/jbc.RA118.005411

Kramer, A., Green, J., Pollard, J., Jr., & Tugendreich, S. (2014). Causal analysis approaches in Ingenuity Pathway Analysis. *Bioinformatics, 30*(4), 523-530. doi:10.1093/bioinformatics/btt703

Kumar, A., Davuluri, G., Silva, R. N. E., Engelen, M., Ten Have, G. A. M., Prayson, R., . . . Dasarathy, S. (2017). Ammonia lowering reverses sarcopenia of cirrhosis by restoring skeletal muscle proteostasis. *Hepatology, 65*(6), 2045-2058. doi:10.1002/hep.29107

Kumar, A., Davuluri, G., Welch, N., Kim, A., Gangadhariah, M., Allawy, A., . . . Dasarathy, S. (2019). Oxidative stress mediates ethanol-induced skeletal muscle mitochondrial dysfunction and dysregulated protein synthesis and autophagy. *Free Radic Biol Med, 145*, 284-299. doi:10.1016/j.freeradbiomed.2019.09.031

Kumar, A., Welch, N., Mishra, S., Bellar, A., Silva, R. N., Li, L., . . . Dasarathy, S. (2021). Metabolic reprogramming during hyperammonemia targets mitochondrial function and postmitotic senescence. *JCI Insight, 6*(24). doi:10.1172/jci.insight.154089

Martens, M., Ammar, A., Riutta, A., Waagmeester, A., Slenter, D. N., Hanspers, K., . . . Kutmon, M. (2021). WikiPathways: connecting communities. *Nucleic Acids Res, 49*(D1), D613-D621. doi:10.1093/nar/gkaa1024

Perez-Riverol, Y., Bai, J., Bandla, C., Garcia-Seisdedos, D., Hewapathirana, S., Kamatchinathan, S., . . . Vizcaino, J. A. (2022). The PRIDE database resources in 2022: a hub for mass spectrometry-based proteomics evidences. *Nucleic Acids Res, 50*(D1), D543-D552. doi:10.1093/nar/gkab1038

Qiu, J., Thapaliya, S., Runkana, A., Yang, Y., Tsien, C., Mohan, M. L., . . . Dasarathy, S. (2013). Hyperammonemia in cirrhosis induces transcriptional regulation of myostatin by an NF-kappaB-mediated mechanism. *Proc Natl Acad Sci U S A, 110*(45), 18162-18167. doi:10.1073/pnas.1317049110

Qiu, J., Tsien, C., Thapalaya, S., Narayanan, A., Weihl, C. C., Ching, J. K., . . . Dasarathy, S. (2012). Hyperammonemia-mediated autophagy in skeletal muscle contributes to sarcopenia of cirrhosis. *Am J Physiol Endocrinol Metab, 303*(8), E983-993. doi:10.1152/ajpendo.00183.2012

Rath, S., Sharma, R., Gupta, R., Ast, T., Chan, C., Durham, T. J., . . . Mootha, V. K. (2021). MitoCarta3.0: an updated mitochondrial proteome now with sub-organelle localization and pathway annotations. *Nucleic Acids Res, 49*(D1), D1541-D1547. doi:10.1093/nar/gkaa1011

Schneider, C. A., Rasband, W. S., & Eliceiri, K. W. (2012). NIH Image to ImageJ: 25 years of image analysis. *Nat Methods, 9*(7), 671-675. doi:10.1038/nmeth.2089

Sigrist, C. J., de Castro, E., Cerutti, L., Cuche, B. A., Hulo, N., Bridge, A., . . . Xenarios, I. (2013). New and continuing developments at PROSITE. *Nucleic Acids Res, 41*(Database issue), D344-347. doi:10.1093/nar/gks1067

Singh, S. S., Kumar, A., Welch, N., Sekar, J., Mishra, S., Bellar, A., . . . Dasarathy, S. (2021). Multiomics-Identified Intervention to Restore Ethanol-Induced Dysregulated Proteostasis and Secondary Sarcopenia in Alcoholic Liver Disease. *Cell Physiol Biochem, 55*(1), 91-116. doi:10.33594/000000327

Subramanian, A., Tamayo, P., Mootha, V. K., Mukherjee, S., Ebert, B. L., Gillette, M. A., . . . Mesirov, J. P. (2005). Gene set enrichment analysis: a knowledge-based approach for interpreting genome-wide expression profiles. *Proc Natl Acad Sci U S A, 102*(43), 15545-15550. doi:10.1073/pnas.0506580102

Szklarczyk, D., Gable, A. L., Nastou, K. C., Lyon, D., Kirsch, R., Pyysalo, S., . . . von Mering, C. (2021). The STRING database in 2021: customizable protein-protein networks, and functional characterization of user-uploaded gene/measurement sets. *Nucleic Acids Res, 49*(D1), D605-D612. doi:10.1093/nar/gkaa1074

Tabb, D. L. (2015). The SEQUEST family tree. *J Am Soc Mass Spectrom, 26*(11), 1814-1819. doi:10.1007/s13361-015-1201-3

Tacutu, R., Thornton, D., Johnson, E., Budovsky, A., Barardo, D., Craig, T., . . . de Magalhaes, J. P. (2018). Human Ageing Genomic Resources: new and updated databases. *Nucleic Acids Res, 46*(D1), D1083-D1090. doi:10.1093/nar/gkx1042

Titov, D. V., Cracan, V., Goodman, R. P., Peng, J., Grabarek, Z., & Mootha, V. K. (2016). Complementation of mitochondrial electron transport chain by manipulation of the NAD+/NADH ratio. *Science, 352*(6282), 231-235. doi:10.1126/science.aad4017

Tsien, C., Davuluri, G., Singh, D., Allawy, A., Ten Have, G. A., Thapaliya, S., . . . Dasarathy, S. (2015). Metabolic and molecular responses to leucine-enriched branched chain amino acid supplementation in the skeletal muscle of alcoholic cirrhosis. *Hepatology, 61*(6), 2018-2029. doi:10.1002/hep.27717

UniProt, C. (2021). UniProt: the universal protein knowledgebase in 2021. *Nucleic Acids Res, 49*(D1), D480-D489. doi:10.1093/nar/gkaa1100

Welch, N., Dasarathy, J., Runkana, A., Penumatsa, R., Bellar, A., Reen, J., . . . Dasarathy, S. (2020). Continued muscle loss increases mortality in cirrhosis: Impact of aetiology of liver disease. *Liver Int, 40*(5), 1178-1188. doi:10.1111/liv.14358

Welch, N., Singh, S. S., Kumar, A., Dhruba, S. R., Mishra, S., Sekar, J., . . . Dasarathy, S. (2021). Integrated multiomics analysis identifies molecular landscape perturbations during hyperammonemia in skeletal muscle and myotubes. *J Biol Chem, 297*(3), 101023. doi:10.1016/j.jbc.2021.101023

Zhao, M., Chen, L., & Qu, H. (2016). CSGene: a literature-based database for cell senescence genes and its application to identify critical cell aging pathways and associated diseases. *Cell Death Dis, 7*, e2053. doi:10.1038/cddis.2015.414
