# Supplementary material for: Dysregulated cellular redox status during hyperammonemia causes mitochondrial dysfunction and senescence by inhibiting sirtuin‐mediated deacetylation
Source: Aging Cell. 2023 Apr 26;22(7):e13852. doi: 10.1111/acel.13852 (PMC10352558; doi:10.1111/acel.13852)
Supplement: Supplementary file 2 — Data S2: Supporting Information. [file ACEL-22-e13852-s001.docx]

**Key Resource Table**

| **REAGENT OR RESOURCE** | **SOURCE** | **IDENTIFIER** |
| --- | --- | --- |
| **Antibodies** | | |
| Acetyl lysine (1C6) mouse monoclonal; 1:2000 | Novus biologicals, Centennial CO | Cat# NB100-74339 |
| SirT1 (D1D7) Rabbit monoclonal;(1:2000) | Cell Signaling Technology, Danvers, MA | CST # 9475S |
| SirT2 Rabbit monoclonal (D4S6J);(1:2000) | Cell Signaling Technology, Danvers, MA | CST# 12672 |
| SirT3 Rabbit Monoclonal ((D22A3);(1:2000) | Cell Signaling Technology, Danvers, MA | CST#5490S |
| SirT4 Rabbit Monoclonal; (1:2000) | Biovision, Milpitas, CA USA | 3224-30T |
| SirT5 Rabbit Monocolnal (D8C3); (1:2000) | Cell Signaling Technology, Danvers, MA | CST# 8782S |
| SirT6 Rabbit Monoclonal (D8D12); (1:2000) | Cell Signaling Technology, Danvers, MA | CST # 12486S |
| SirT7 Rabbit Monoclonal (D3K5A); (1:2000) | Cell Signaling Technology, Danvers, MA | CST#5360S |
| Mouse monoclonal anti-α-Tubulin (TU-02 ); (1:5000) | Santa Cruz  Biotechnology,  Dallas, Texas | Cat# sc-8035 |
| Mouse monoclonal anti-β-Actin (C4 ); (1:10000) | Santa Cruz  Biotechnology,  Dallas, Texas | Cat# sc-47778 |
| Mouse monoclonal NMNAT ( B10), (1:1000) | Santa Cruz  Biotechnology,  Dallas, Texas | Cat # sc-515206 |
| Nicotinamide phosphoribosyltransferase (NAMPT) Mouse monoclonal (E3); ( 1:1000) | [Santa Cruz,](https://www.google.com/search?sxsrf=ALiCzsbrKngxJ-kN_NUiuNaW0LWLx_lIBw:1653080562642&q=Santa+Cruz,+California&stick=H4sIAAAAAAAAAOPgE-LSz9U3KEjJy8kyUeIAsXMMcrO1tLKTrfTzi9IT8zKrEksy8_NQOFYZqYkphaWJRSWpRcWLWMWCE_NKEhWci0qrdBScE3My0_KL8jITd7Ay7mJn4mAAAIvgkB9lAAAA&sa=X&ved=2ahUKEwi5l-L9_O73AhUJg4kEHSkSAaMQmxMoAHoECGcQAg)  [Dallas, TX](https://www.google.com/search?sxsrf=ALiCzsae9GcmDNsVgM9u6Zl6oiU3Cfu_QQ:1653080618750&q=Dallas&stick=H4sIAAAAAAAAAOPgE-LUz9U3SDPOTUpT4gAzjYoKtbSyk63084vSE_MyqxJLMvPzUDhWGamJKYWliUUlqUXFi1jZXBJzchKLd7Ay7mJn4mAAAF2dbatUAAAA&sa=X&ved=2ahUKEwjK_8KY_e73AhXZk4kEHe8zC60QmxMoAHoECFkQAg) | sc-393444 |
| Anti-FLAG mosue monoclonal M2; (1:1000) | Milipore sigma, St. Louis, MO, USA | F3165-.2MG |
| Rabbit polyclonal anti-VDAC; 1:2000) | Cell Signaling Technology, Danvers, MA | Cat# 4866 |
| Rabbit polyclonal anti-phospho-p53 (Ser15); (1:1000) | Cell Signaling  Technology,  Danver, MA | Cat# 9284 |
| Mouse monoclonal anti-p53 (1C12); (1:2000)) | Cell Signaling  Technology,  Danver, MA | Cat# 2524s |
| Rabbit polyclonal anti-p21 (F-5); (1:2000)) | Santa Cruz  Biotechnology,  Dallas, Texas | Cat# sc-6246 |
| Rabbit monoclonal anti-p16 INK4A (D7C1M);  (1:2000) | Cell Signaling  Technology,  Danver, MA | Cat# 80772T |
| Rabbit polyclonal Acetyl-NF-κB p65 (Lys310) Antibody ;(1:2000) | Cell Signaling  Technology,  Danver, MA | CST #3045 |
| Rabbit polyclonal TATA binding prottien (TBP); (1:5000) | Cell Signaling  Technology,  Danver, MA | CST# 8515s |
| **Chemicals, peptides, and recombinant proteins** | | |
| (+)-Sodium L-ascorbate (Ascorbate) | Sigma-Aldrich, St. Louis, MO | Cat# A7631 |
| (L)-Malic Acid (Malate) | Sigma-Aldrich, St. Louis, MO | Cat# M1000 |
| β-Mercaptoethanol | Sigma-Aldrich, St. Louis, MO | Cat# M3148 |
| 3-[(3-cholamidopropyl) dimethylammonio]-1-  propanesulfonate (CHAPS) | EMD Millipore  Corp., Billerica,  MA | Cat# 220201 |
| 3,3’ -Diaminobenzidine (DAB) | Sigma-Aldrich, St. Louis, MO | Cat# D8001 |
| 4-Methlyumbeliferyl β-D-galactopyranoside (MUG) | Sigma-Aldrich, St. Louis, MO | Cat# M1633 |
| Adenosine diphosphate (ADP) | Sigma-Aldrich, St. Louis, MO | Cat# A5285 |
| Adenosine triphosphate (ATP) | Sigma-Aldrich, St. Louis, MO | Cat# A2383 |
| Ammonium acetate | Sigma-Aldrich, St. Louis, MO | Cat# A7330 |
| Antimycin a | Sigma-Aldrich, St. Louis, MO | Cat# A8674 |
| Bis-Tris | Sigma-Aldrich, St. Louis, MO | Cat# B4429 |
| Carbonyl cyanide p-trifluoro-methoxyphenyl  hydrazone (FCCP) | Sigma-Aldrich, St. Louis, MO | Cat# C2920 |
| Coomassie brilliant stain G-250 | Bio-Rad  Laboratories,  Hercules, CA | Cat# 1610406 |
| Coomassie brilliant stain R-250 | Bio-Rad  Laboratories,  Hercules, CA | Cat# 1610400 |
| Cytochrome c from bovine heart | Sigma-Aldrich, St. Louis, MO | Cat# C2037 |
| Digitonin | Sigma-Aldrich, St.Louis, MO | Cat# D5628 |
| Magnesium chloride | Sigma-Aldrich, St.  Louis, MO | Cat# 208337 |
| Magnesium sulfate heptahydrate | Sigma-Aldrich, St.  Louis, MO | Cat# M5921 |
| MitoSOX™ red mitochondrial superoxide indicator | Invitrogen,  ThermoFisher  Scientific,  Waltham, MA | Cat# M36008 |
| N-Dodecyl β-D-maltoside 98% | Sigma-Aldrich, St.  Louis, MO | Cat# D4641 |
| ,N,N,N’-Tetramethyl-p-phenylenediamine  dihydrochloride (TMPD) | Sigma-Aldrich, St.  Louis, MO | Cat# T3134 |
| NativePage™ 3 to 12%, bis-tris, 1.0 mm, mini gel | ThermoFisher  Scientific,  Waltham, MA | Cat# BN1001BOX |
| Nicotinamide adenine dinucleotide (NADH) | Sigma-Aldrich, St.  Louis, MO | Cat# N6005 |
| Nitro blue tetrazolium chloride (NBT) | ThermoFisher  Scientific,  Waltham, MA | Cat# N6495 |
| Oligomycin | Sigma-Aldrich, St. Louis, MO | Cat# O4876 |
| Phenylmethanesulfonylfluoride (PMSF) | Sigma-Aldrich, St.  Louis, MO | Cat# P-7626 |
| Rotenone | Sigma-Aldrich, St.Louis, MO | Cat# R8875 |
| Sodium azide | Sigma-Aldrich, St.Louis, MO | Cat# S2002 |
| Sodium chloride | Fisher Scientific,  Hampton. NH | Cat#S640 |
| Sodium succinate dibasic hexahydrate (Succinate) | Sigma-Aldrich, St. Louis, MO | Cat# S2378 |
| Tricine | Sigma-Aldrich, St. Louis, MO | Cat# T0377 |
| Ammonium formate | Sigma-Aldrich, St. Louis, MO | Cat# 516961-100G |
| Ammonium citrate | Sigma-Aldrich, St.Louis, MO | 247561-100G |
| Ammonium bicarbonate | [Santa Cruz,](https://www.google.com/search?sxsrf=ALiCzsbrKngxJ-kN_NUiuNaW0LWLx_lIBw:1653080562642&q=Santa+Cruz,+California&stick=H4sIAAAAAAAAAOPgE-LSz9U3KEjJy8kyUeIAsXMMcrO1tLKTrfTzi9IT8zKrEksy8_NQOFYZqYkphaWJRSWpRcWLWMWCE_NKEhWci0qrdBScE3My0_KL8jITd7Ay7mJn4mAAAIvgkB9lAAAA&sa=X&ved=2ahUKEwi5l-L9_O73AhUJg4kEHSkSAaMQmxMoAHoECGcQAg)  [Dallas, TX](https://www.google.com/search?sxsrf=ALiCzsae9GcmDNsVgM9u6Zl6oiU3Cfu_QQ:1653080618750&q=Dallas&stick=H4sIAAAAAAAAAOPgE-LUz9U3SDPOTUpT4gAzjYoKtbSyk63084vSE_MyqxJLMvPzUDhWGamJKYWliUUlqUXFi1jZXBJzchKLd7Ay7mJn4mAAAF2dbatUAAAA&sa=X&ved=2ahUKEwjK_8KY_e73AhXZk4kEHe8zC60QmxMoAHoECFkQAg) | CAS 1066-33-7 |
| Ammonium hydroxide | Sigma-Aldrich, St.Louis, MO | Cat# AX1303-3 |
| Nicotinamide Riboside chloride | MuseChem, Fairfield, NJ | Cat# 23111-00-4 |
| **Biological samples** |  |  |
| C57BL/6J male mice 8-10 weeks age | Jax.org | #000664 |
| Sprague Dawley rats (male) | Charles River | Crl:SD |
| Human control patients and patients with cirrhosis | Biorepository at corresponding author’s institution |  |
| **Oligonucleotides** | | |
| Sirt1 primers | Integrated DNA Technologies | FW 5'-CCTTGGAGACTGCGATGTTA-3', RV 5'-GTGTTGGTGGCAACTCTGAT-3 |
| Sirt2 primers | Integrated DNA Technologies | FW 5'-GCAGTGTCAGAGCGTGGTAA-3', RV 5'-CTAGTGGTGCCTTGCTGATG-3' |
| Sirt3 primers | Integrated DNA Technologies | FW 5'-TACAGGCCCAATGTCACTCA-3', RV 5'-ACAGACCGTGCATGTAGCTG-3' |
| Sirt4 primers | Integrated DNA Technologies | FW 5'-CGCTGCTCAAGATCCCTAAG-3', RV 5'-GCGACACAGCTACTCCATCA-3' |
| Sirt5 primers | Integrated DNA Technologies | FW 5'-GACTCAAGACGCCAGAATCC-3', RV 5'-CAGAGGATGTTCCCACCACT-3' |
| Sirt6 primers | Integrated DNA Technologies | FW 5'-CTGGTCTGGAACTCACTGCT-3', RV 5'-CGGGTGTGATTGGTAGAGAG-3' |
| Sirt7 primers | Integrated DNA Technologies | FW 5'-GGCACTTGGTTGTCTACACG-3', RV 5'-GTGATGCTCATGTGGGTGAG-3' |
| **Critical Commercial Assays** | | |
| ATP determination kit Invitrogen, | Thermo Fisher  Scientific,  Waltham, MA | Cat# A22066 |
| HDAC Activity Fluorometric Assay Kit | Biovison, Milpitas, CA USA | Catalog # K339-100 |
| MiR05-kit | O2k-Network Lab,  Innsbruck, Austria | Cat# MiPNet22.10 MiR05-Kit |
| NAD/NADH GLO Assay | Promega, Wisconsin, USA. | G9071 |
| Plasmid Midi Kit | QIAGEN | 12143 |
| RNeasy Plus Universal Mini Kit | QIAGEN | 73404 |
| SIRT3 Activity Assay Kit (Fluorometric) | Abcam, Waltham, MA. | ab156067 |
| SuperScript III First-Strand | Invitrogen (California, USA) | 18080-051 |
| **Experimental models: cell lines** | | |
| C2C12 myotubes | ATCC | CRL-1772 |
| Human inducible pluripotent stem cell derived myotubes (male and female subjects) | Gift from Jonathan Smith (Co-Author) |  |
| **Recombinant DNA** | | |
| Lactobacillus brevis with a C-terminal Flag-tag without (LbNOX) and with a mitochondrial localizing sequence (MitoLbNOX), were subcloned into pcDNA3.1 | Genescript | SC1626 |
| **Deposited Data** | | |
| Dysregulated cellular redox status during hyperammonemia causes mitochondrial dysfunction by inhibiting sirtuin mediated deacetylation | This paper | <http://www.proteomexchange.org> with dataset identifier PXD033430 and 10.6019/PXD033430  Reviewer account details:  **Username:**[reviewer_pxd033430@ebi.ac.uk](mailto:reviewer_pxd033430@ebi.ac.uk)  **Password:**Vhguxruy |
| Mitochondrial responses during hyperammonemia | ProteomeXChange PRIDE repository | <http://www.proteomexchange.org> with dataset identifier PXD026955 and 10.6019/PXD026955 |
| Integrated molecular landscape perturbations underlie cellular responses during hyperammonemia | NCBI Gene Expression Omnibus | SuperSeries GSE171645  <https://www.ncbi.nlm.nih.gov/geo/query/acc.cgi?acc=GSE171642>  <https://www.ncbi.nlm.nih.gov/geo/query/acc.cgi?acc=GSE171643>  <https://www.ncbi.nlm.nih.gov/geo/query/acc.cgi?acc=GSE171644> |
| **Software and algorithms** | | |
| SPSS | IBM, USA |  |
| ImageJ | NIH | imagej.nih.gov/ij |
| FlowJo | FlowJO LLC | https://www.bdbiosciences.com/en-us/products/software/flowjo-v10-software |
| IPA | QIAGEN, Hilden,  Germany | https://digitalinsights.qiagen.com/pro  ducts-overview/discovery-insightsportfolio/  analysis-andvisualization/  qiagen-ipa/ |
| R Studio | Open source | Open Source https://www.rstudio.com/products/rst  udio/download/ |
| DatLab | Oroboros, | Innsbruck, Austria  Cat# 27142-01 |
| Adobe Illustrator 2021 | Adobe,Z San Jose,  CA | https://www.adobe.com/products/ |
