# Supplementary material for: Dysregulated cellular redox status during hyperammonemia causes mitochondrial dysfunction and senescence by inhibiting sirtuin‐mediated deacetylation
Source: Aging Cell. 2023 Apr 26;22(7):e13852. doi: 10.1111/acel.13852 (PMC10352558; doi:10.1111/acel.13852)
Supplement: Supplementary file 3 — Data S3: Supporting Information. [file ACEL-22-e13852-s002.docx]

Index of Tables

| **S.Table number** | **Figure supported** | **Data description** |
| --- | --- | --- |
| 1 | S.Fig1,A-F | Cross-dataset matching of molecules within cellular, mouse, and human models& sirtuin expression heatmaps |
| 2 | S.Fig 2,A-C | Multiomics expression of features in the sirtuin signaling pathway |
| 3 | S.Fig 3,A-C | Multiomics expression of features in the NAD signaling pathway |
| 4 | S.Fig 4,A-C | Protein-protein interactions within the NAD and sirtuin signaling pathways in unbiased data from hyperammonemic myotubes and skeletal muscle |
| 5 | S.Fig 6,A-G | Differential expression of p65NFkB targets during hyperammonemia |
| No S.Table—Data uploaded to proteinXchange | S.Fig 7,A,B | Acetylome QC |
| 6 | Fig.2,B,C,D,F; S.Fig.8,B-F | Untargeted acetylomics in myotubes during hyperammonemia reveal novel therapeutic targets and Clustering of acetylated proteins in myotubes |
| 7 | S.Fig 9,A-C | Oxidative phosphorylation pathway components during hyperammonemia. |
| 8 | Data for S.Fig 10 | Correlation plot of differentially expressed acetylated sites |
| 9 | Data for S.Fig 11, A-G | Sub-correlation plots, circle diagrams, and motifs |
