# Supplementary material for: Dysregulated cellular redox status during hyperammonemia causes mitochondrial dysfunction and senescence by inhibiting sirtuin‐mediated deacetylation
Source: Aging Cell. 2023 Apr 26;22(7):e13852. doi: 10.1111/acel.13852 (PMC10352558; doi:10.1111/acel.13852)

S.Fig 1

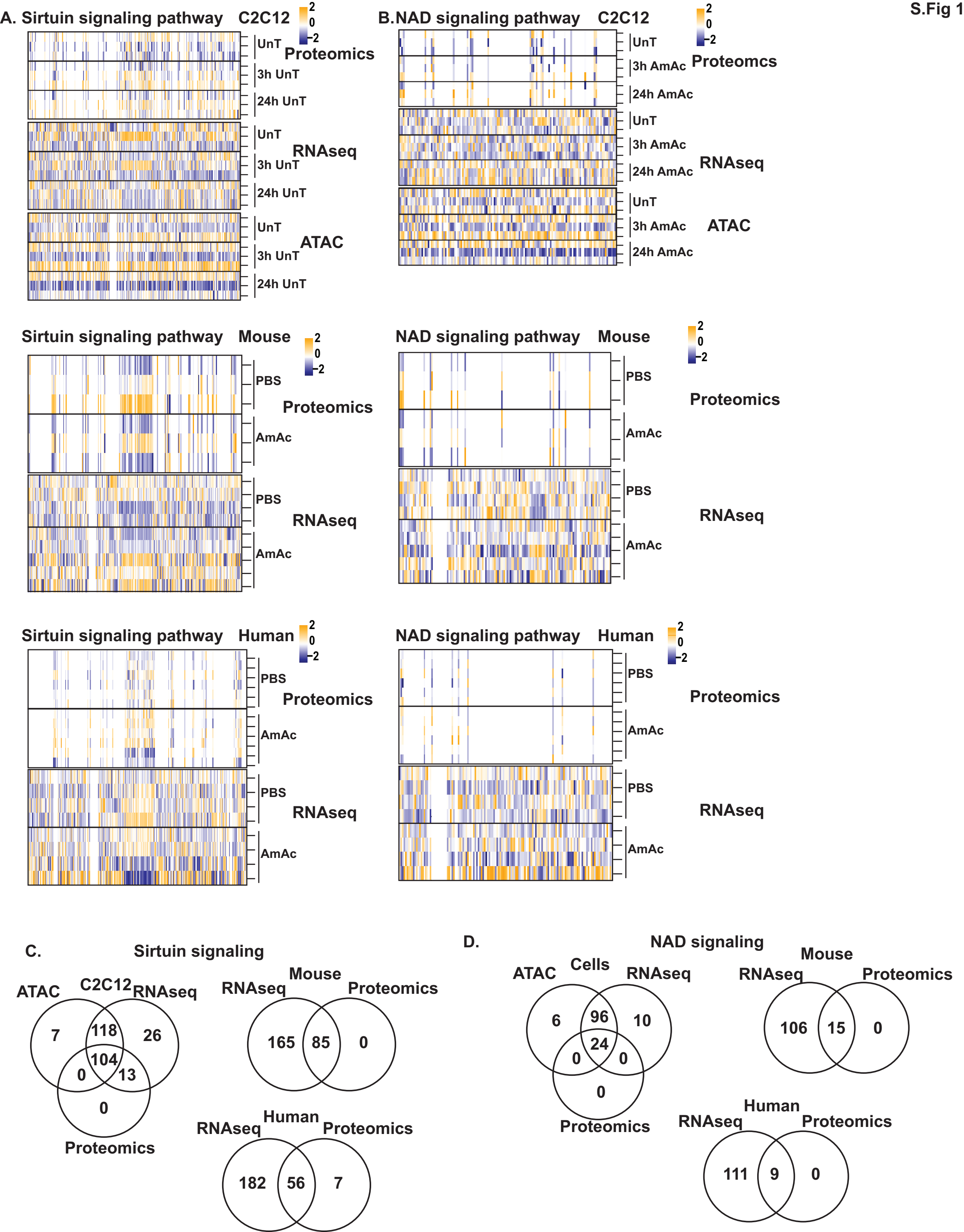
A. Sirtuin signaling pathway C2C12
2
0
-2
UnT
3h UnT
24h UnT
Proteomics
RNAseq
ATAC
B. NAD signaling pathway C2C12
3h AmAc
24h AmAc
Proteomcs
Sirtuin signaling pathway Mouse
PBS
AmAc
NAD signaling pathway Mouse
Sirtuin signaling pathway Human
NAD signaling pathway Human
C.
Sirtuin signaling
ATAC
C2C12
7
118
26
104
0
13
Mouse
165
85
Human
182
56
D.
NAD signaling
Cells
6
96
10
24
106
15
111
9

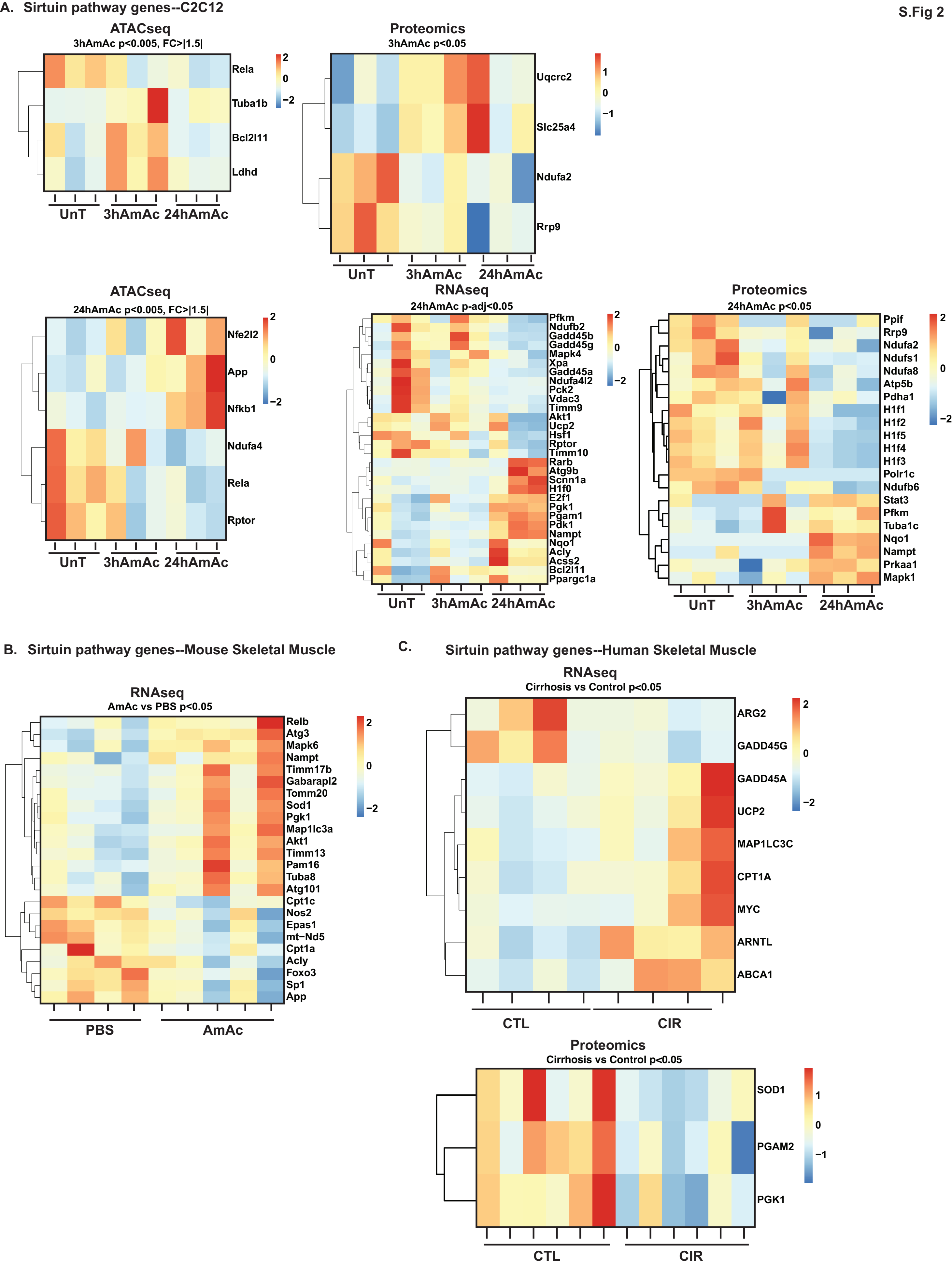
S.Fig 2
A. Sirtuin pathway genes--C2C12
ATACseq
3hAmAc p<0.005, FC>|1.5|
Rela
Tuba1b
Bcl2l11
Ldhd
UnT
3hAmAc
24hAmAc
Proteomics
3hAmAc p<0.05
Uqcrc2
Slc25a4
Ndufa2
Rrp9
ATACseq
24hAmAc p<0.005, FC>|1.5|
Nfe2l2
App
Nfkb1
Ndufa4
Rela
Rptor
RNAseq
24hAmAc p-adj<0.05
Pfkm
Ndufb2
Gadd45b
Gadd45g
Mapk4
Xpa
Gadd45a
Ndufa4l2
Pck2
Vdac3
Timm9
Akt1
Ucp2
Hsf1
Rptor
Timm10
Rarb
Atg9b
Scnn1a
H1f0
E2f1
Pgk1
Pgam1
Pdk1
Nampt
Nqo1
Acly
Acss2
Bcl2l11
Ppargc1a
Proteomics
24hAmAc p<0.05
Ppif
Rrp9
Ndufa2
Ndufs1
Ndufa8
Atp5b
Pdha1
H1f1
H1f2
H1f5
H1f4
H1f3
Polr1c
Ndufb6
Stat3
Pfkm
Tuba1c
Nqo1
Nampt
Prkaa1
Mapk1
B. Sirtuin pathway genes--Mouse Skeletal Muscle
RNAseq
AmAc vs PBS p<0.05
Relb
Atg3
Mapk6
Nampt
Timm17b
Gabarapl2
Tomm20
Sod1
Pgk1
Map1lc3a
Akt1
Timm13
Pam16
Tuba8
Atg101
Cpt1c
Nos2
Epas1
mt−Nd5
Cpt1a
Acly
Foxo3
Sp1
App
PBS
AmAc
C. Sirtuin pathway genes--Human Skeletal Muscle
RNAseq
Cirrhosis vs Control p<0.05
ARG2
GADD45G
GADD45A
UCP2
MAP1LC3C
CPT1A
MYC
ARNTL
ABCA1
CTL
CIR
Proteomics
Cirrhosis vs Control p<0.05
SOD1
PGAM2
PGK1
CTL
CIR

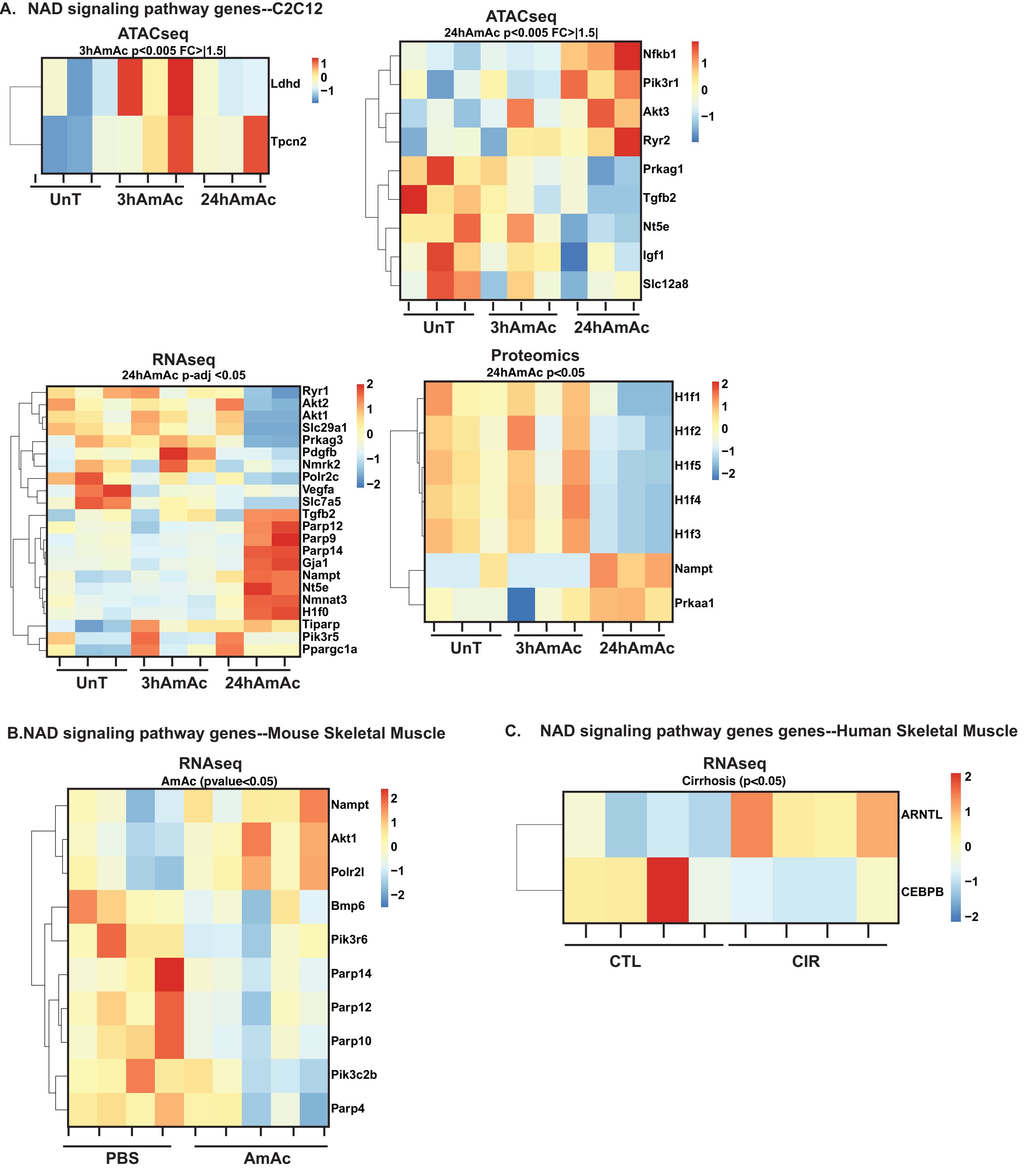
A. NAD signaling pathway genes--C2C12
ATACseq
3hAmAc p<0.005 FC>|1.5|
Ldhd
Tpcn2
1
0
−1
UnT
3hAmAc
24hAmAc
ATACseq
24hAmAc p<0.005 FC>|1.5|
Nfkb1
Pik3r1
Akt3
Ryr2
Prkag1
Tgfb2
Nt5e
Igf1
Slc12a8
1
0
−1
UnT
3hAmAc
24hAmAc
RNAseq
24hAmAc p-adj <0.05
Ryr1
Akt2
Akt1
Slc29a1
Prkag3
Pdgfb
Nmrk2
Polr2c
Vegfa
Slc7a5
Tgfb2
Parp12
Parp9
Parp14
Gja1
Nampt
Nt5e
Nmnat3
H1f0
Tiparp
Pik3r5
Ppargc1a
2
1
0
−1
−2
UnT
3hAmAc
24hAmAc
Proteomics
24hAmAc p<0.05
H1f1
H1f2
H1f5
H1f4
H1f3
Nampt
Prkaa1
2
1
0
−1
−2
UnT
3hAmAc
24hAmAc
B.NAD signaling pathway genes--Mouse Skeletal Muscle
RNAseq
AmAc (pvalue<0.05)
Nampt
Akt1
Polr2l
Bmp6
Pik3r6
Parp14
Parp12
Parp10
Pik3c2b
Parp4
2
1
0
−1
−2
PBS
AmAc
C. NAD signaling pathway genes genes--Human Skeletal Muscle
RNAseq
Cirrhosis (p<0.05)
ARNTL
CEBPB
2
1
0
−1
−2
CTL
CIR

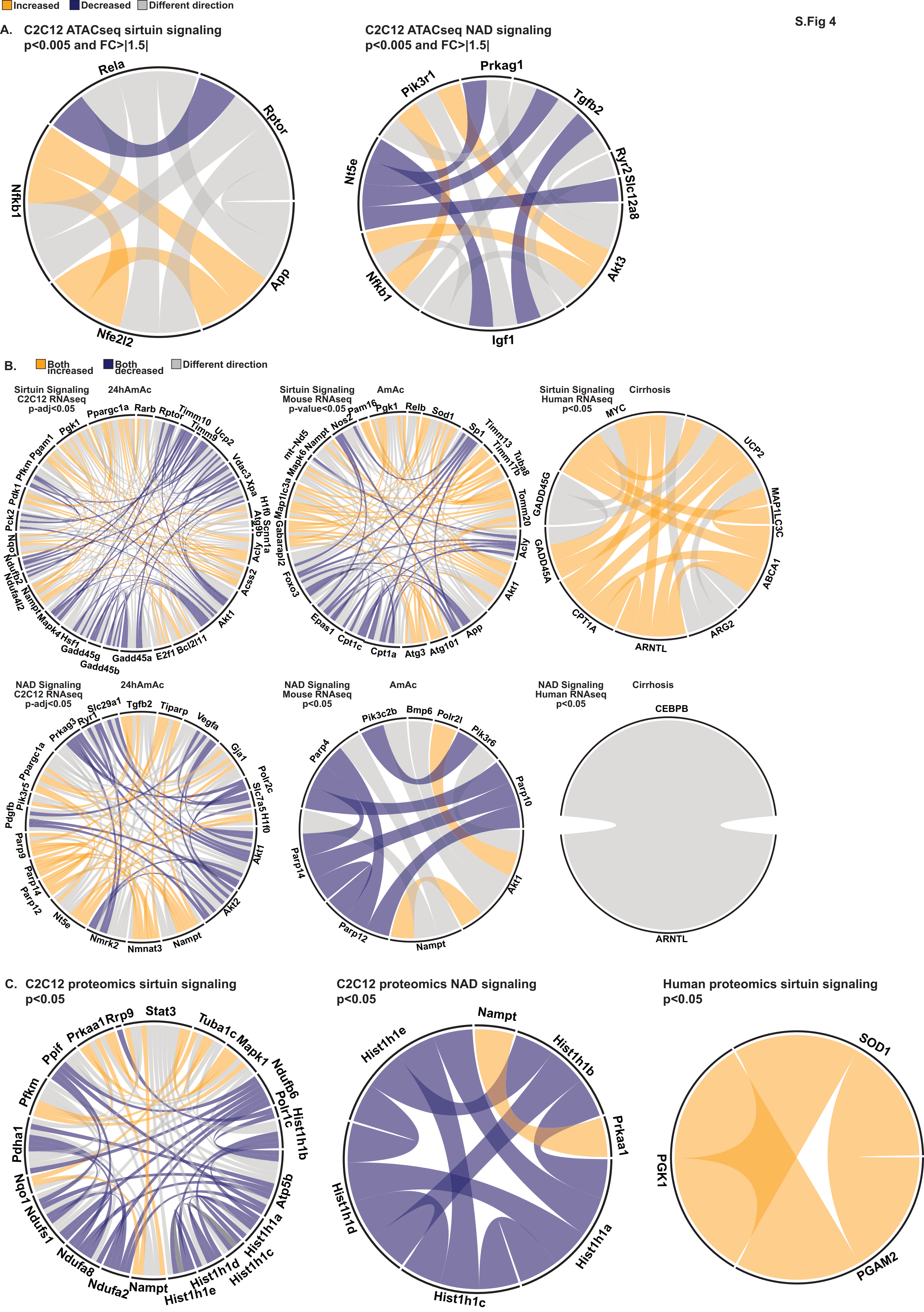

Increased
Decreased
Different direction
S.Fig 4
A. C2C12 ATACseq sirtuin signaling
p<0.005 and FC>|1.5|
Rela
Rptor
Nfkb1
App
Nfe2l2
C2C12 ATACseq NAD signaling
p<0.005 and FC>|1.5|
Prkag1
Pik3r1
Tgfb2
Nt5e
Ryr2
Slc12a8
Akt3
Nfkb1
Igf1
B.
Both increased
Both decreased
Different direction
Sirtuin Signaling
C2C12 RNAseq
p-adj<0.05
24hAmAc
Ppargc1a
Rarb
Rptor
Timm10
Timm9
Ucp2
Vdac3
Xpa
H1f0
Scnn1a
Atg9b
Acly
Acss2
Akt1
Bcl2l11
E2f1
Gadd45a
Gadd45b
Gadd45g
Hsf1
Mapk4
Nampt
Ndufa4l2
Ndufb2
Nqo1
Pck2
Pdk1
Pfkm
Pgam1
Pgk1
Sirtuin Signaling
Mouse RNAseq
p-value<0.05
AmAc
Pam16
Pgk1
Relb
Sod1
Sp1
Timm13
Timm17b
Tuba8
Tomm20
Acly
Akt1
App
Atg101
Atg3
Cpt1a
Cpt1c
Epas1
Foxo3
Gabarapl2
Map1lc3a
Mapk6
mt-Nd5
Nampt
Nos2
Sirtuin Signaling
Human RNAseq
p<0.05
Cirrhosis
MYC
UCP2
MAP1LC3C
ABCA1
ARG2
ARNTL
CPT1A
GADD45A
GADD45G
NAD Signaling
C2C12 RNAseq
p-adj<0.05
24hAmAc
Slc29a1
Tgfb2
Tiparp
Vegfa
Gja1
Polr2c
Slc7a5
H1f0
Akt1
Akt2
Nampt
Nmnat3
Nmrk2
Nt5e
Parp12
Parp14
Parp9
Pdgfb
Pik3r5
Ppargc1a
Prkag3
Ryr1
NAD Signaling
Mouse RNAseq
p<0.05
AmAc
Pik3c2b
Bmp6
Polr2l
Pik3r6
Parp10
Akt1
Nampt
Parp12
Parp14
Parp4
NAD Signaling
Human RNAseq
p<0.05
Cirrhosis
CEBPB
ARNTL
C. C2C12 proteomics sirtuin signaling
p<0.05
Stat3
Tuba1c
Mapk1
Ndufb6
Polr1c
Hist1h1b
Atp5b
Hist1h1a
Hist1h1c
Hist1h1d
Hist1h1e
Nampt
Ndufa2
Ndufa8
Ndufs1
Nqo1
Pdha1
Pfkm
Ppif
Prkaa1
Rrp9
C2C12 proteomics NAD signaling
p<0.05
Nampt
Hist1h1b
Prkaa1
Hist1h1a
Hist1h1c
Hist1h1d
Hist1h1e
Human proteomics sirtuin signaling
p<0.05
SOD1
PGAM2
PGK1

S.Fig 5

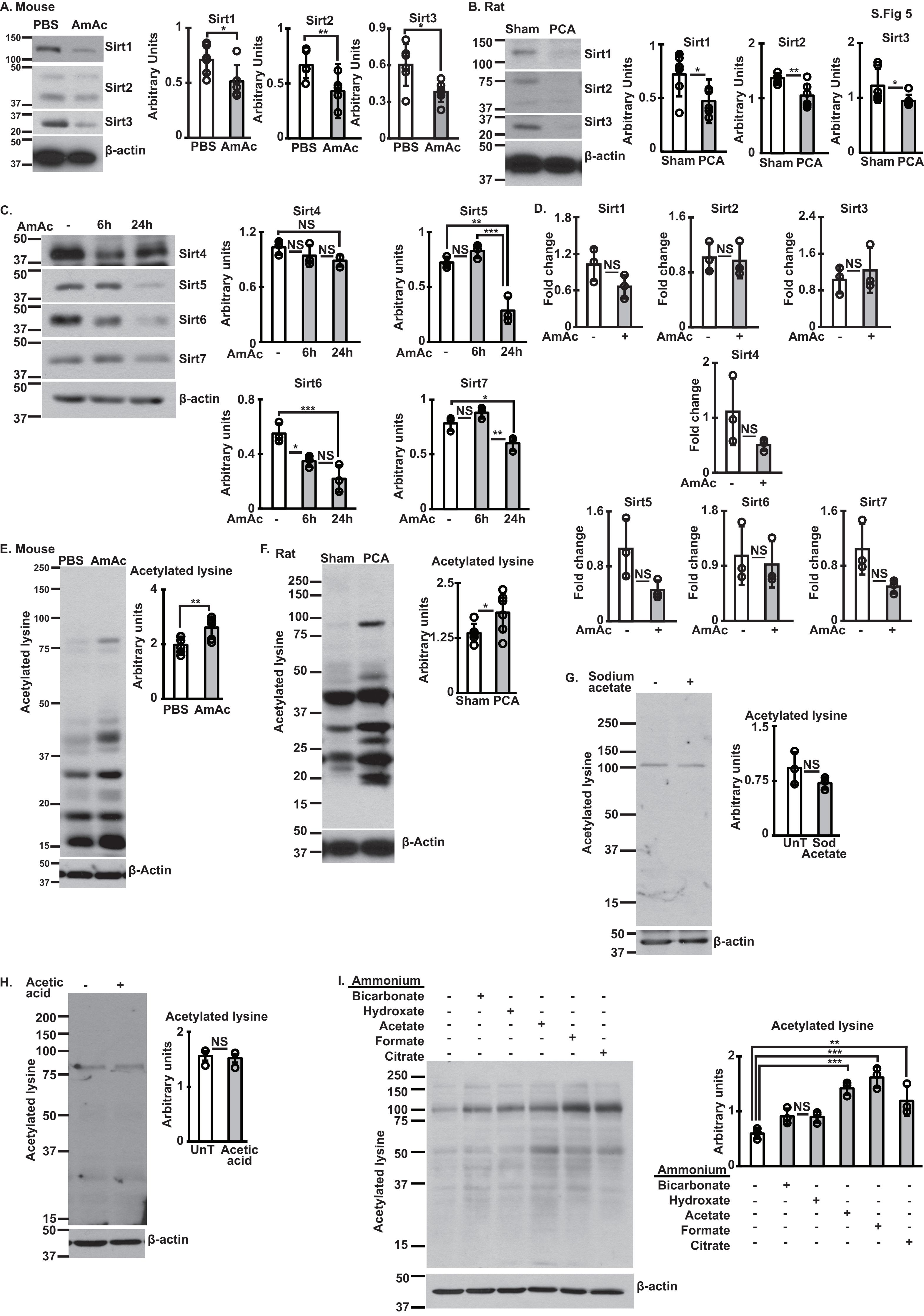

A. Mouse
PBS AmAc
Sirt1
Sirt2
Sirt3
β-actin
Arbitrary Units
PBS AmAc
B. Rat
Sham PCA
Sirt1
Sirt2
Sirt3
β-actin
Sham PCA
C.
AmAc - 6h 24h
Sirt4
Sirt5
Sirt6
Sirt7
β-actin
Arbitrary units
NS
D.
Sirt1
Sirt2
Sirt3
Sirt4
Sirt5
Sirt6
Sirt7
Fold change
AmAc - +
E. Mouse
PBS AmAc
Acetylated lysine
β-Actin
Arbitrary units
F. Rat
Sham PCA
Acetylated lysine
β-Actin
G. Sodium acetate - +
Acetylated lysine
β-actin
UnT Sod Acetate
H. Acetic acid - +
Acetylated lysine
β-actin
UnT Acetic acid
I. Ammonium
Bicarbonate
Hydroxate
Acetate
Formate
Citrate
Acetylated lysine
β-actin
Arbitrary units

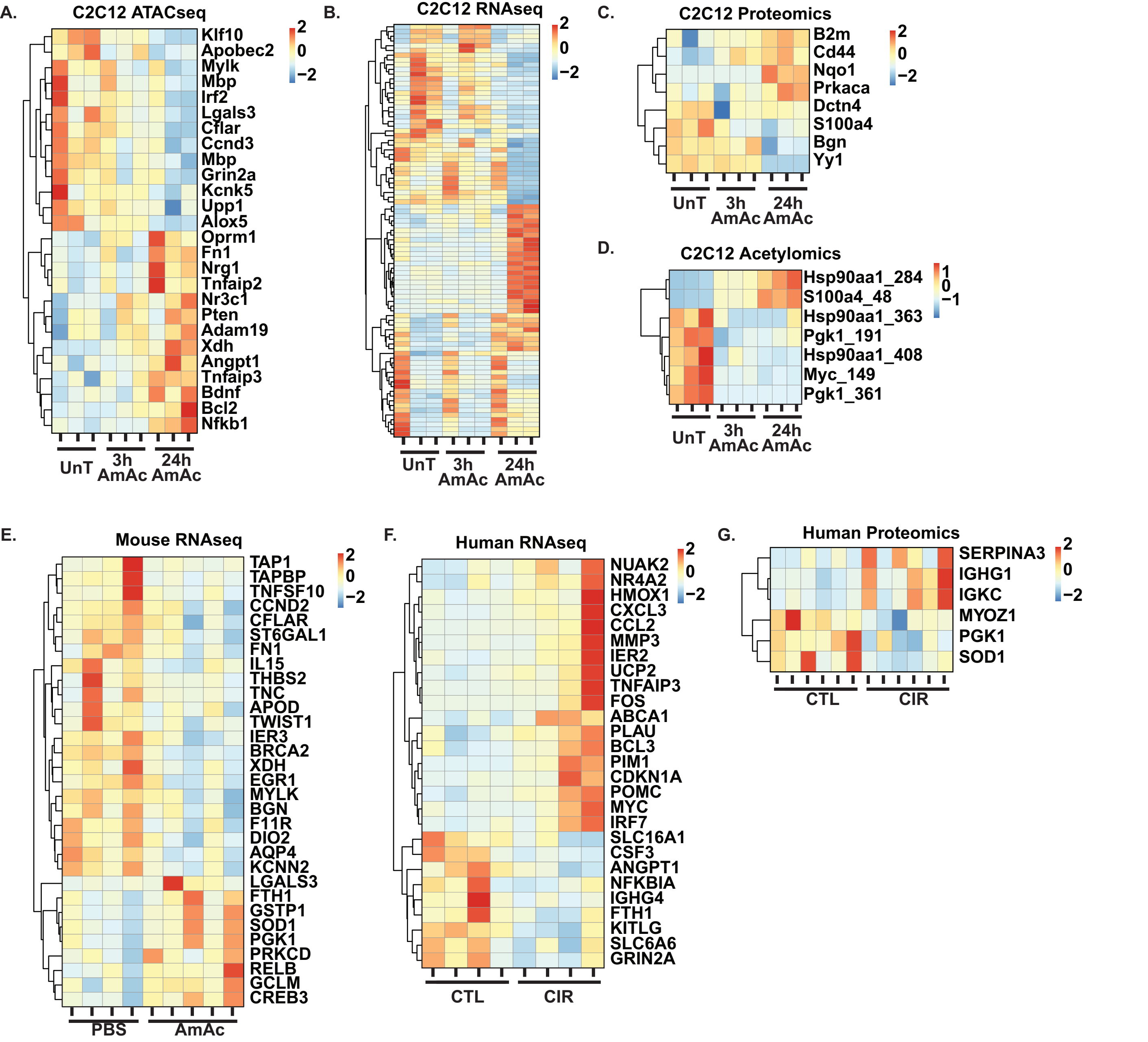
A.
C2C12 ATACseq
Klf10
Apobec2
Mylk
Mbp
Irf2
Lgals3
Cflar
Ccnd3
Mbp
Grin2a
Kcnk5
Upp1
Alox5
Oprm1
Fn1
Nrg1
Tnfaip2
Nr3c1
Pten
Adam19
Xdh
Angpt1
Tnfaip3
Bdnf
Bcl2
Nfkb1
2
0
−2
UnT
3h AmAc
24h AmAc
B.
C2C12 RNAseq
2
0
−2
UnT
3h AmAc
24h AmAc
C.
C2C12 Proteomics
B2m
Cd44
Nqo1
Prkaca
Dctn4
S100a4
Bgn
Yy1
2
0
−2
UnT
3h AmAc
24h AmAc
D.
C2C12 Acetylomics
Hsp90aa1_284
S100a4_48
Hsp90aa1_363
Pgk1_191
Hsp90aa1_408
Myc_149
Pgk1_361
1
0
−1
UnT
3h AmAc
24h AmAc
E.
Mouse RNAseq
TAP1
TAPBP
TNFSF10
CCND2
CFLAR
ST6GAL1
FN1
IL15
THBS2
TNC
APOD
TWIST1
IER3
BRCA2
XDH
EGR1
MYLK
BGN
F11R
DIO2
AQP4
KCNN2
LGALS3
FTH1
GSTP1
SOD1
PGK1
PRKCD
RELB
GCLM
CREB3
2
0
−2
PBS
AmAc
F.
Human RNAseq
NUAK2
NR4A2
HMOX1
CXCL3
CCL2
MMP3
IER2
UCP2
TNFAIP3
FOS
ABCA1
PLAU
BCL3
PIM1
CDKN1A
POMC
MYC
IRF7
SLC16A1
CSF3
ANGPT1
NFKBIA
IGHG4
FTH1
KITLG
SLC6A6
GRIN2A
2
0
−2
CTL
CIR
G.
Human Proteomics
SERPINA3
IGHG1
IGKC
MYOZ1
PGK1
SOD1
2
0
−2
CTL
CIR

Acetylome QC

A.

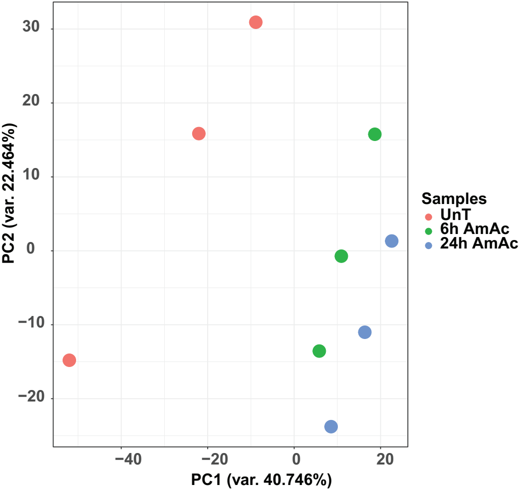

B.

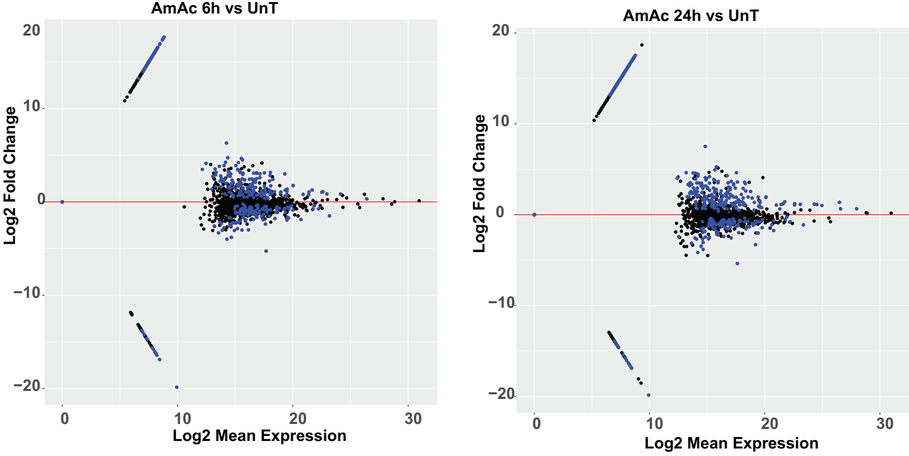

S.Fig 8

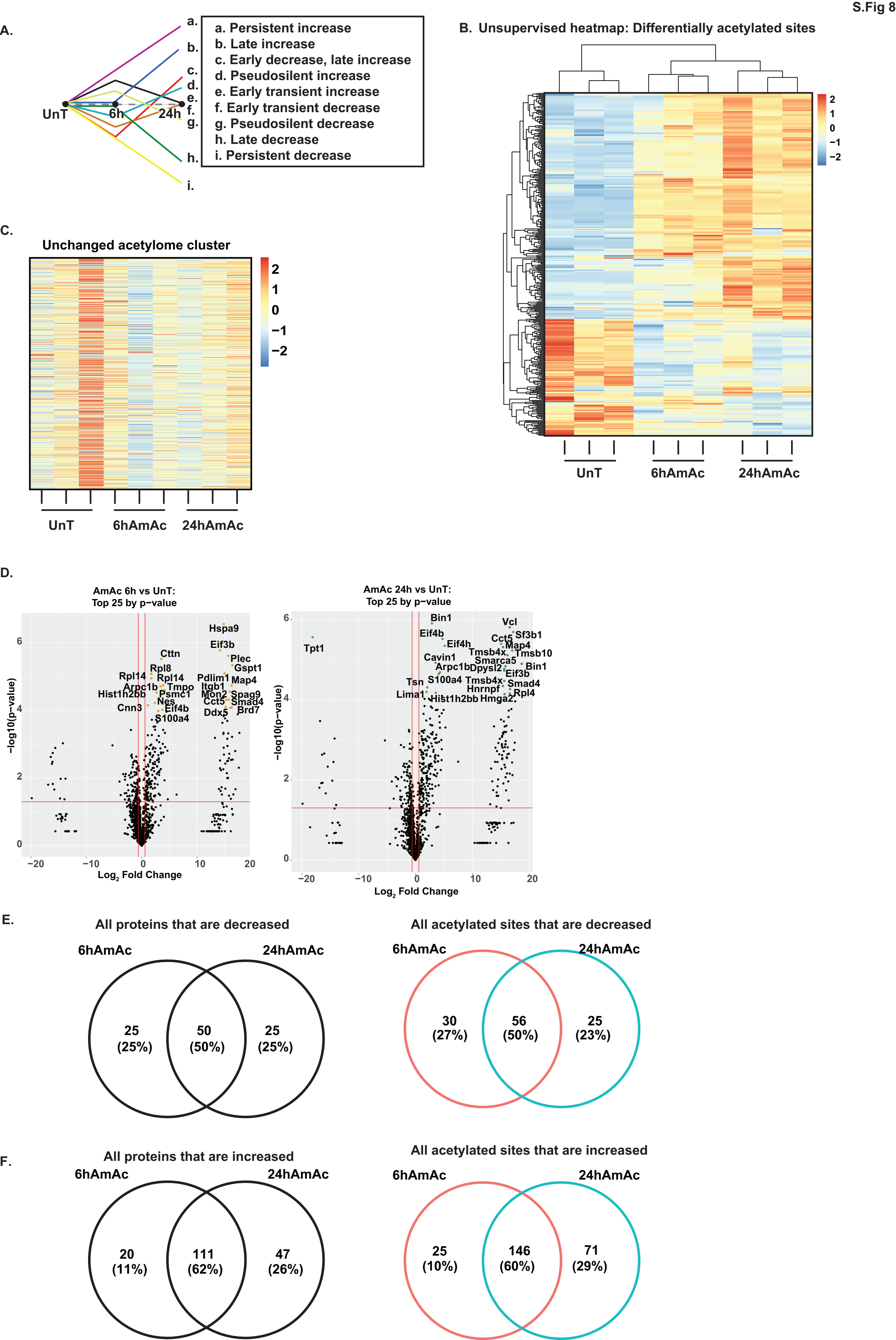
A.
a. Persistent increase
b. Late increase
c. Early decrease, late increase
d. Pseudosilent increase
e. Early transient increase
f. Early transient decrease
g. Pseudosilent decrease
h. Late decrease
i. Persistent decrease
UnT
6h
24h
B. Unsupervised heatmap: Differentially acetylated sites
UnT
6hAmAc
24hAmAc
C.
Unchanged acetylome cluster
UnT
6hAmAc
24hAmAc
D.
AmAc 6h vs UnT:
Top 25 by p−value
Hspa9
Eif3b
Cttn
Plec
Gspt1
Rpl8
Rpl14
Rpl14
Pdlim1
Map4
Arpc1b
Tmpo
Itgb1
Hist1h2bb
Psmc1
Mon2
Spag9
Nes
Cct5
Smad4
Cnn3
Eif4b
Brd7
Ddx5
S100a4
−log10(p−value)
Log2 Fold Change
AmAc 24h vs UnT:
Top 25 by p−value
Bin1
Vcl
Eif4b
Sf3b1
Tpt1
Cct5
Eif4h
Map4
Tmsb4x
Tmsb10
Cavin1
Smarca5
Arpc1b
Dpysl2
Bin1
Eif3b
Tsn
S100a4
Tmsb4x
Smad4
Hnrnpf
Lima1
Rpl4
Hist1h2bb
Hmga2
E.
All proteins that are decreased
6hAmAc
24hAmAc
25 (25%)
50 (50%)
25 (25%)
All acetylated sites that are decreased
6hAmAc
24hAmAc
30 (27%)
56 (50%)
25 (23%)
F.
All proteins that are increased
6hAmAc
24hAmAc
20 (11%)
111 (62%)
47 (26%)
All acetylated sites that are increased
6hAmAc
24hAmAc
25 (10%)
146 (60%)
71 (29%)

S.Fig 9

Decreased expression — Increased expression

- 0 +

Non-significant expression

A. 24hAmAc vs UnT: Oxidative phosphorylation

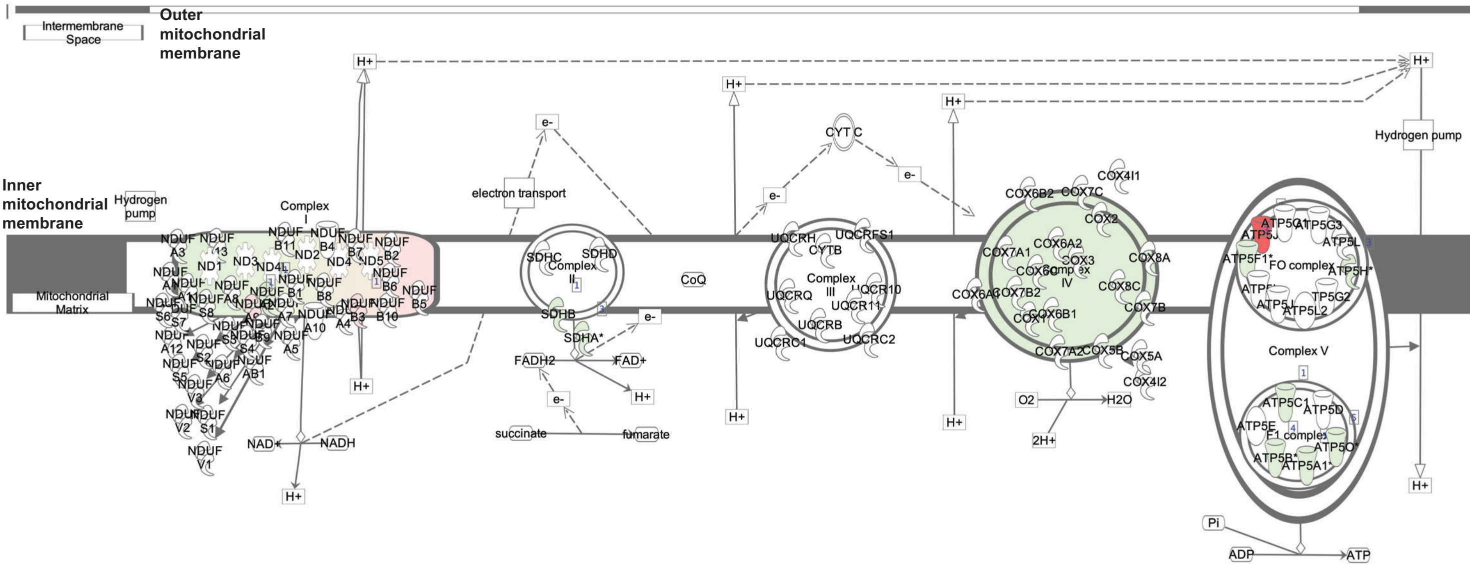

B. 24hAmAc vs 6hAmAc: Oxidative phosphorylation

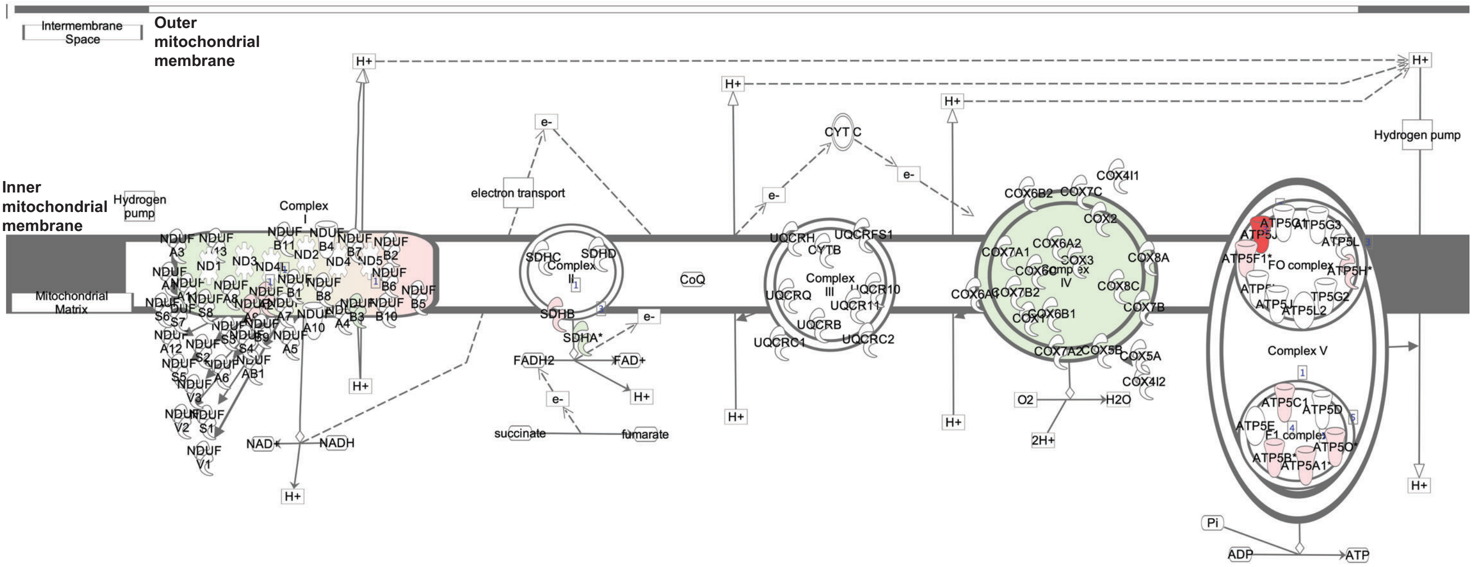

C. 6hAmAc vs UnT: Oxidative phosphorylation

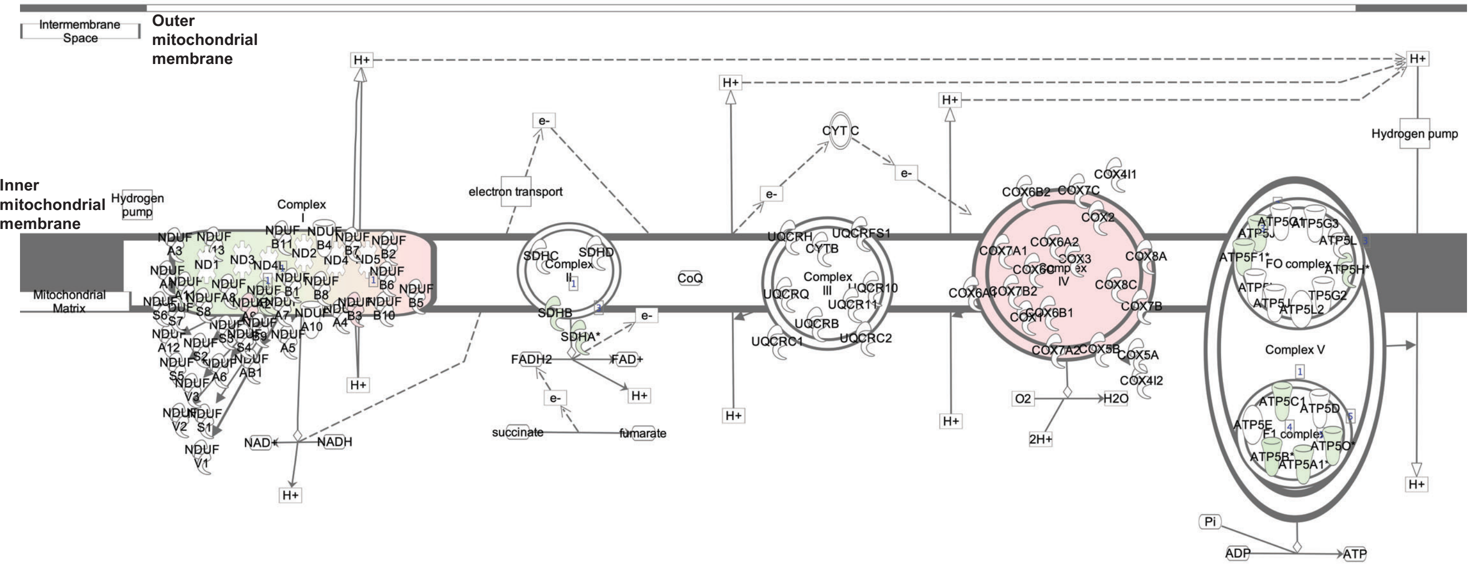

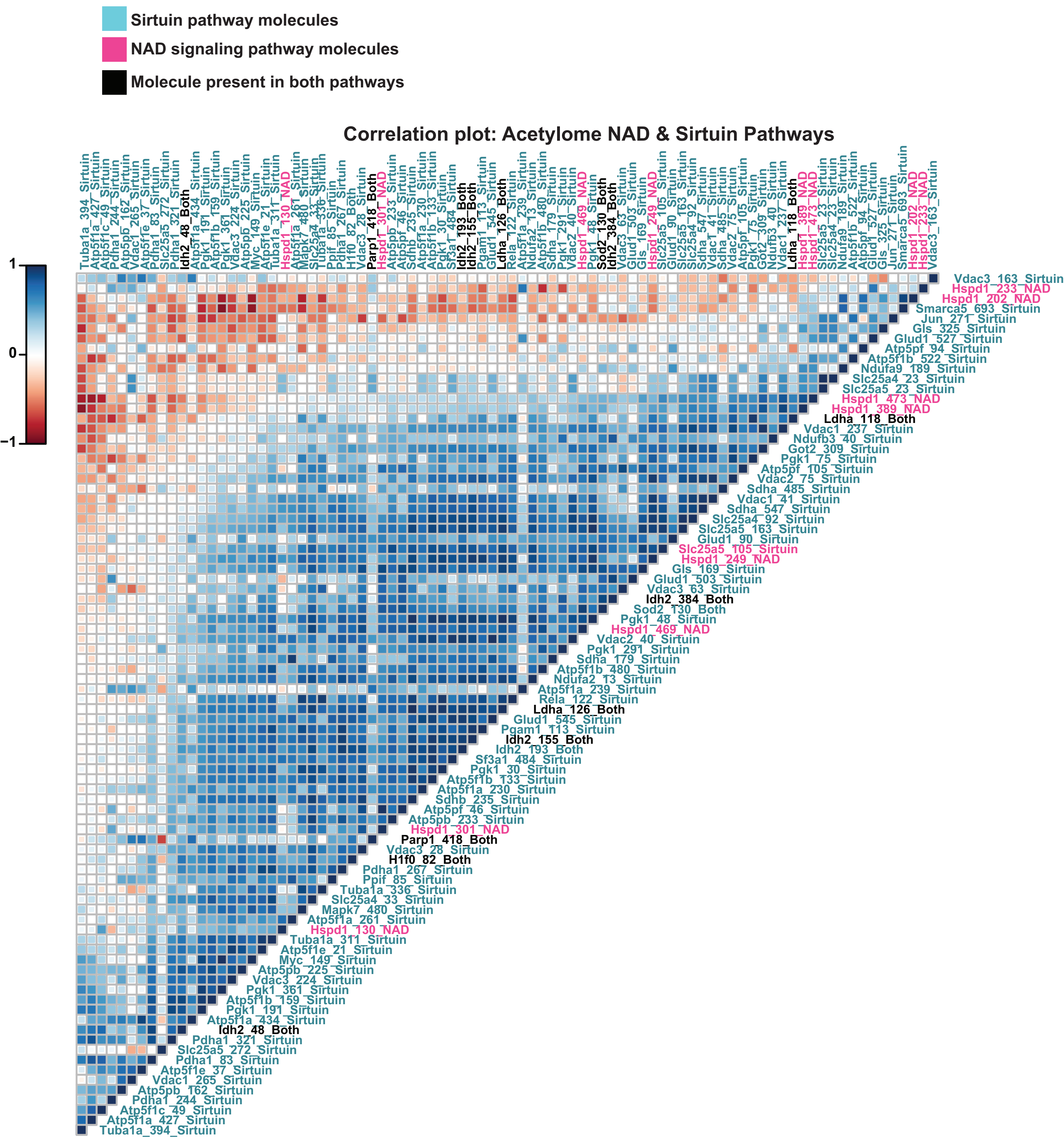
Sirtuin pathway molecules
NAD signaling pathway molecules
Molecule present in both pathways
Correlation plot: Acetylome NAD & Sirtuin Pathways
1
0
−1
Tuba1a_394_Sirtuin
Atp5f1a_427_Sirtuin
Atp5f1c_49_Sirtuin
Pdha1_244_Sirtuin
Atp5pb_162_Sirtuin
Vdac1_265_Sirtuin
Atp5f1e_37_Sirtuin
Pdha1_83_Sirtuin
Slc25a5_272_Sirtuin
Pdha1_321_Sirtuin
Idh2_48_Both
Atp5f1a_434_Sirtuin
Pgk1_191_Sirtuin
Atp5f1b_159_Sirtuin
Pgk1_361_Sirtuin
Vdac3_224_Sirtuin
Atp5pb_225_Sirtuin
Myc_149_Sirtuin
Atp5f1e_21_Sirtuin
Tuba1a_311_Sirtuin
Hspd1_130_NAD
Atp5f1a_261_Sirtuin
Mapk7_480_Sirtuin
Slc25a4_33_Sirtuin
Tuba1a_336_Sirtuin
Ppif_85_Sirtuin
Pdha1_267_Sirtuin
H1f0_82_Both
Vdac3_28_Sirtuin
Parp1_418_Both
Hspd1_301_NAD
Atp5pb_233_Sirtuin
Atp5pf_46_Sirtuin
Sdhb_235_Sirtuin
Atp5f1a_230_Sirtuin
Atp5f1b_133_Sirtuin
Pgk1_30_Sirtuin
Sf3a1_484_Sirtuin
Idh2_193_Both
Idh2_155_Both
Pgam1_113_Sirtuin
Glud1_545_Sirtuin
Ldha_126_Both
Rela_122_Sirtuin
Atp5f1a_239_Sirtuin
Ndufa2_13_Sirtuin
Atp5f1b_480_Sirtuin
Sdha_179_Sirtuin
Pgk1_291_Sirtuin
Vdac2_40_Sirtuin
Hspd1_469_NAD
Pgk1_48_Sirtuin
Sod2_130_Both
Idh2_384_Both
Vdac3_63_Sirtuin
Glud1_503_Sirtuin
Gls_169_Sirtuin
Hspd1_249_NAD
Slc25a5_105_Sirtuin
Glud1_90_Sirtuin
Slc25a5_163_Sirtuin
Slc25a4_92_Sirtuin
Sdha_547_Sirtuin
Vdac1_41_Sirtuin
Sdha_485_Sirtuin
Vdac2_75_Sirtuin
Atp5pf_105_Sirtuin
Pgk1_75_Sirtuin
Got2_309_Sirtuin
Ndufb3_40_Sirtuin
Vdac1_237_Sirtuin
Ldha_118_Both
Hspd1_389_NAD
Hspd1_473_NAD
Slc25a5_23_Sirtuin
Slc25a4_23_Sirtuin
Ndufa9_189_Sirtuin
Atp5f1b_522_Sirtuin
Atp5pf_94_Sirtuin
Glud1_527_Sirtuin
Gls_325_Sirtuin
Jun_271_Sirtuin
Smarca5_693_Sirtuin
Hspd1_202_NAD
Hspd1_233_NAD
Vdac3_163_Sirtuin

A. TCA Cycle

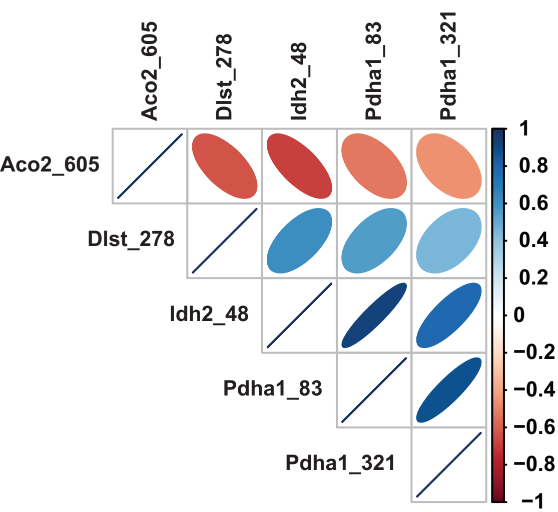

B. Oxidative Phosphorylation

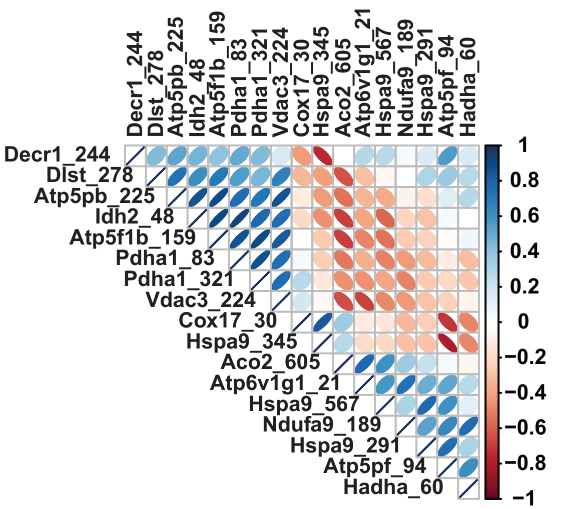

C. Electron Transport Chain

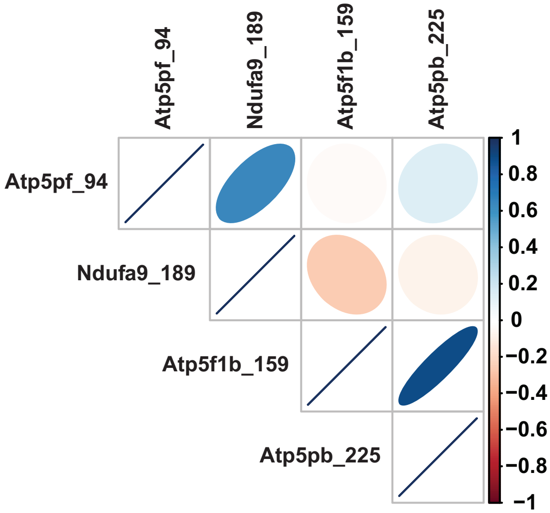

D. ETC and Sirtuin --Acetylome (6hAmAc)
NAD and Sirtuin --Acetylome (6hAmAc)

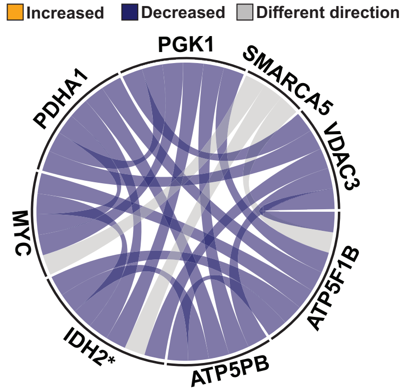

E. ETC and Sirtuin --Acetylome (24hAmAc)

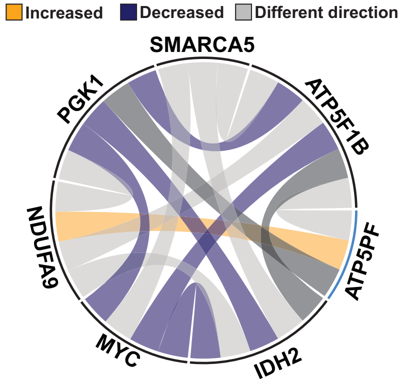

F. NAD and Sirtuin --Acetylome (24hAmAc)

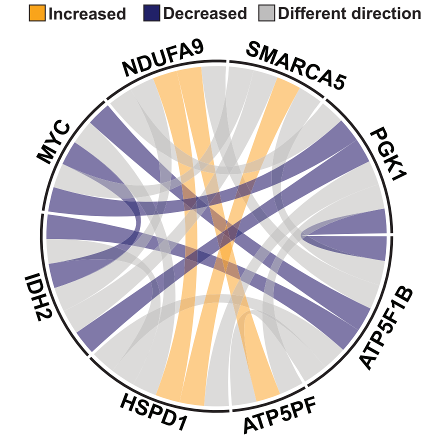

G.

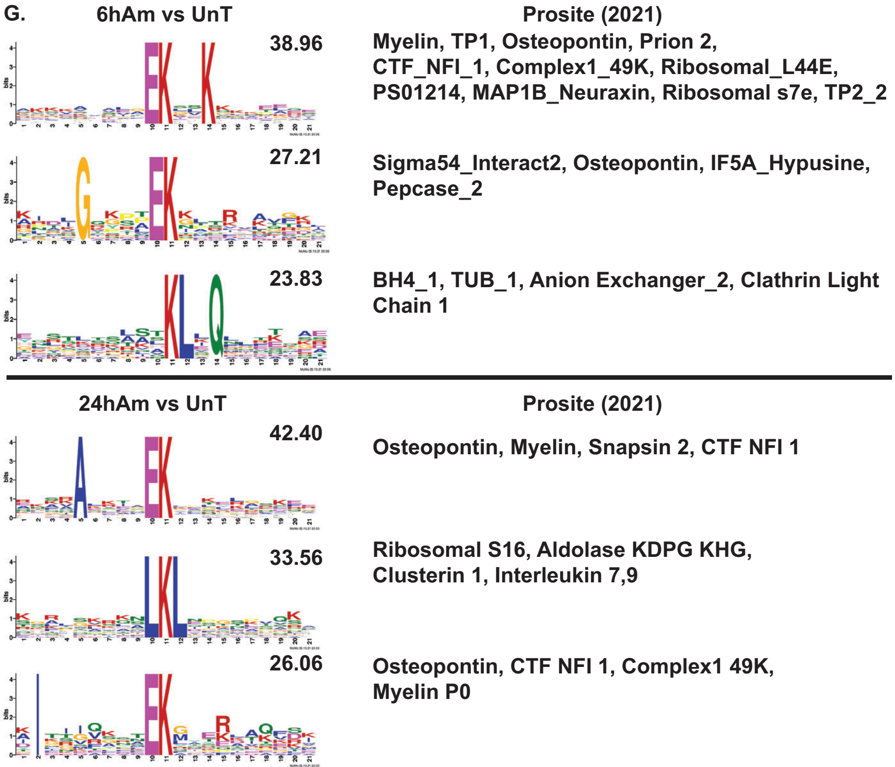

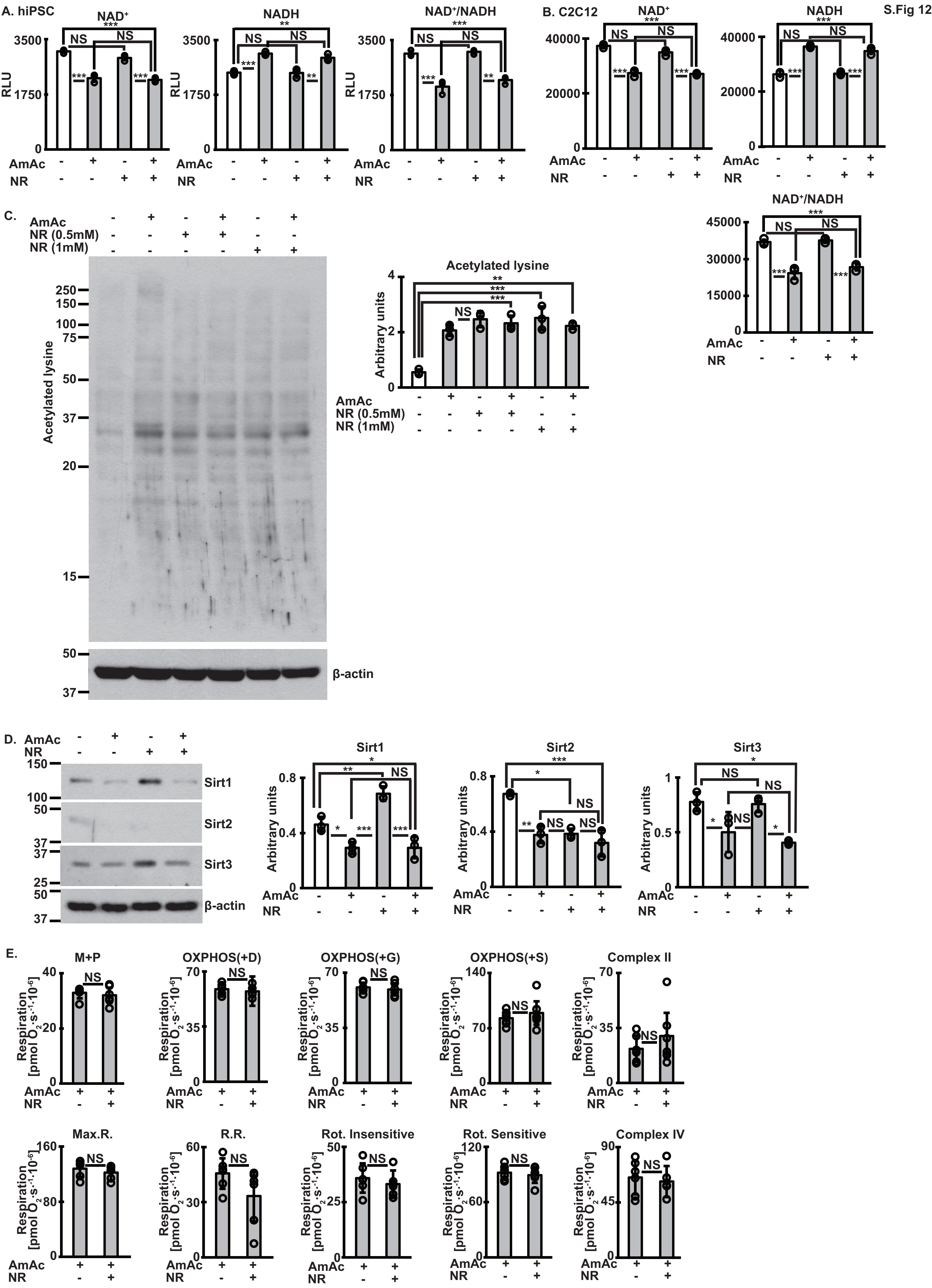
A. hiPSC
NAD+
NADH
NAD+/NADH
RLU
3500
1750
0
AmAc
NR
NS
***
**
B. C2C12
NAD+
NADH
NAD+/NADH
40000
20000
45000
30000
15000
C.
AmAc
NR (0.5mM)
NR (1mM)
Acetylated lysine
250
150
100
75
50
37
20
15
β-actin
Acetylated lysine
Arbitrary units
D.
Sirt1
Sirt2
Sirt3
Arbitrary units
E.
M+P
OXPHOS(+D)
OXPHOS(+G)
OXPHOS(+S)
Complex II
Max.R.
R.R.
Rot. Insensitive
Rot. Sensitive
Complex IV
Respiration
[pmol O2·s-1·10-6]

S.Fig 13

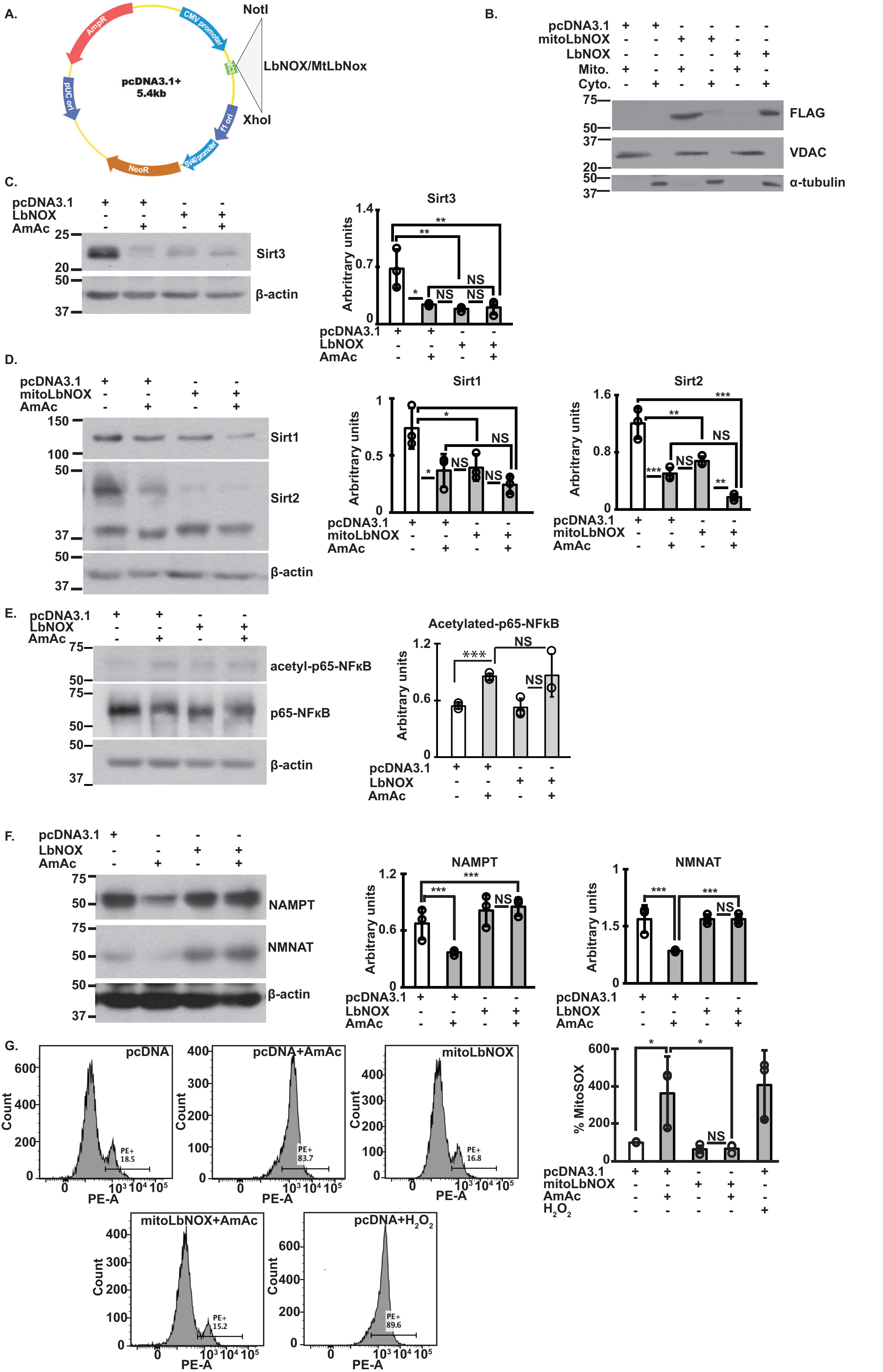

A.
pcDNA3.1+
5.4kb
AmpR
CMV promoter
MCS
NotI
LbNOX/MtLbNox
XhoI
f1 ori
SV40 promoter
NeoR
pUC ori
B.
pcDNA3.1
mitoLbNOX
LbNOX
Mito.
Cyto.
FLAG
VDAC
α-tubulin
C.
pcDNA3.1
LbNOX
AmAc
Sirt3
β-actin
Arbitrary units
NS
D.
mitoLbNOX
Sirt1
Sirt2
E.
acetyl-p65-NFκB
p65-NFκB
Acetylated-p65-NFkB
F.
NAMPT
NMNAT
G.
pcDNA
pcDNA+AmAc
mitoLbNOX
mitoLbNOX+AmAc
pcDNA+H2O2
Count
PE-A
PE+
18.5
83.7
16.8
15.2
89.6
% MitoSOX
H2O2

S.Fig 14

A. hiPSC: Basal protocol

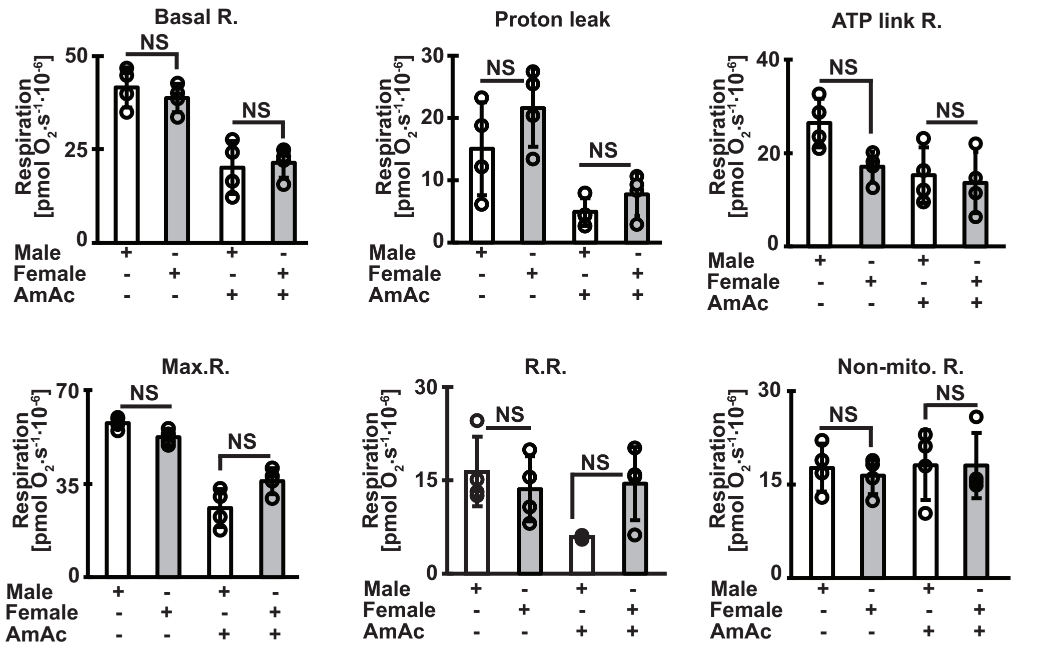

B. hiPSC: Basal protocol

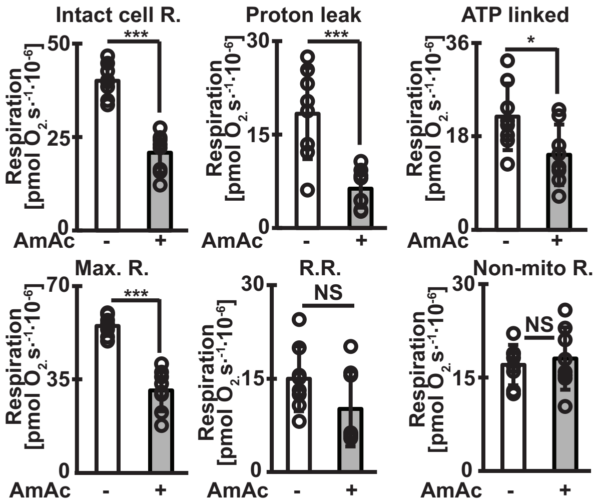

C. hiPSC

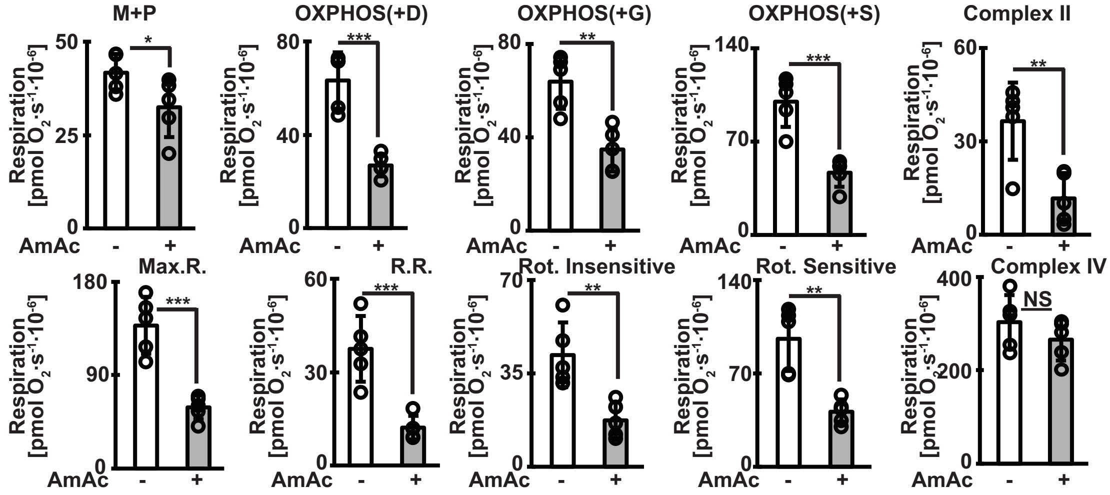

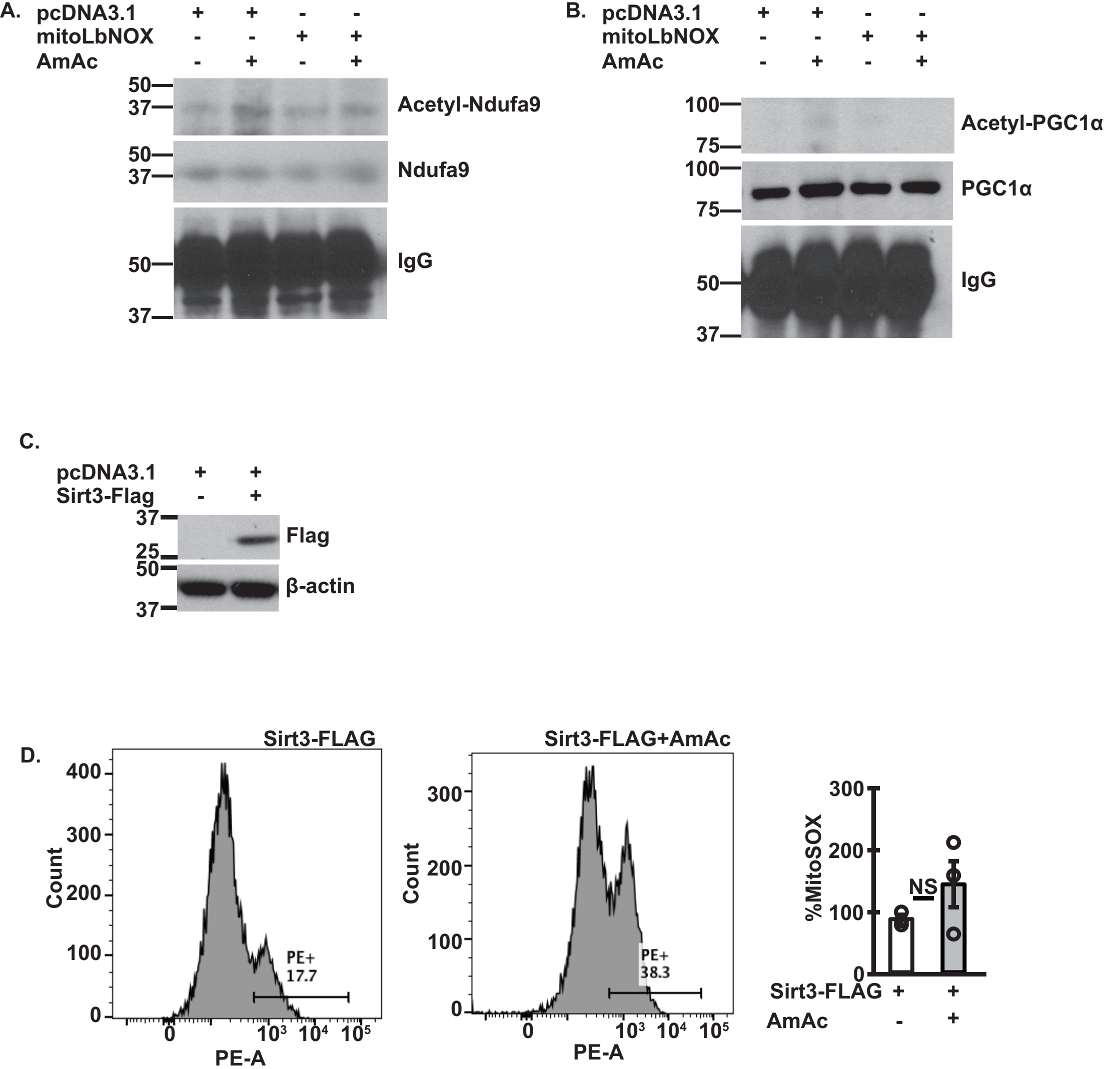
A.
pcDNA3.1 + + - -
mitoLbNOX - - + +
AmAc - + - +
50
37
Acetyl-Ndufa9
50
37
Ndufa9
50
37
IgG
B.
pcDNA3.1 + + - -
mitoLbNOX - - + +
AmAc - + - +
100
75
Acetyl-PGC1α
100
75
PGC1α
50
37
IgG
C.
pcDNA3.1 + +
Sirt3-Flag - +
37
25
Flag
50
37
β-actin
D.
Sirt3-FLAG
Count
400
300
200
100
0
PE-A
0
10³ 10⁴ 10⁵
PE+
17.7
Sirt3-FLAG+AmAc
Count
300
200
100
0
PE-A
0
10³ 10⁴ 10⁵
PE+
38.3
%MitoSOX
300
200
100
0
NS
Sirt3-FLAG + +
AmAc - +

S.Fig 16

A. hiPSC

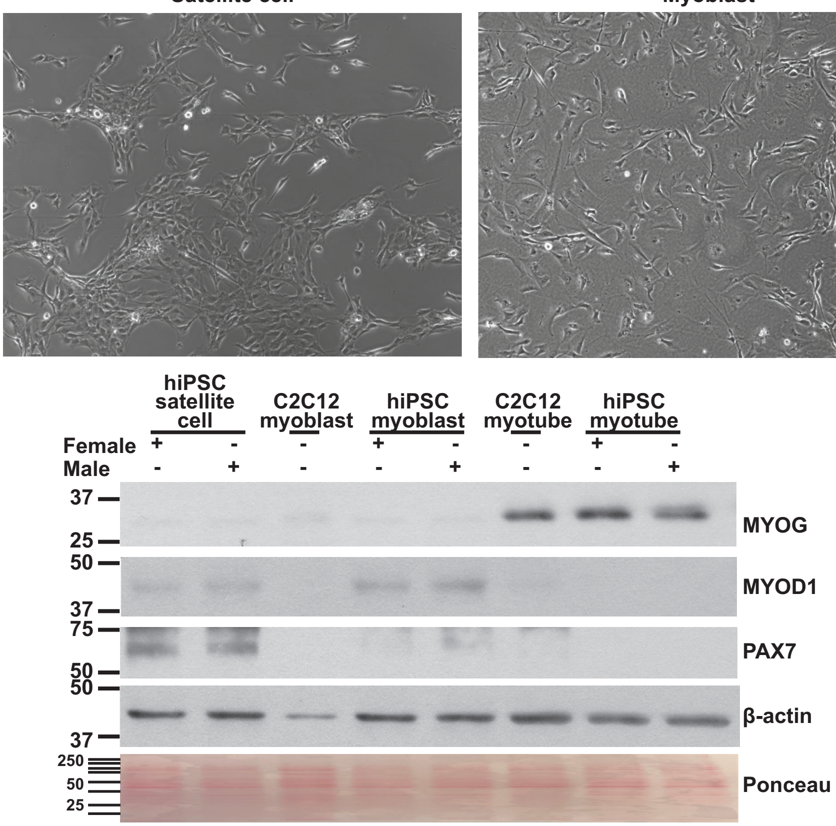

B. hiPSC

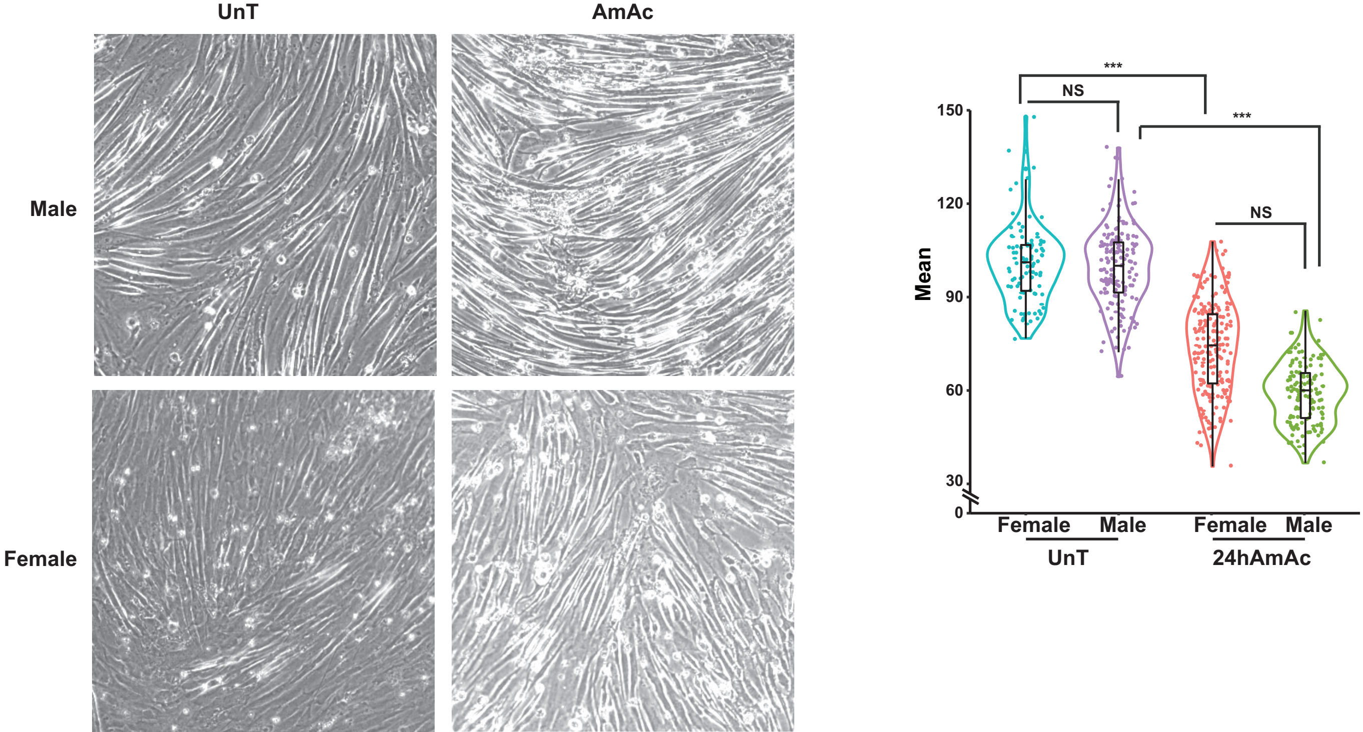

Supplement: Supplementary file 4 — Figure S1. Cross‐dataset matching of molecules within cellular, mouse, and human models. (a, b) Heatmaps and (c, d) Venn diagrams showing unique and shared molecules in the sirtuin and NAD signaling pathways from assay for transposase accessible chromatin sequencing (ATACseq), whole cell RNA sequencing (RNAseq) quantitative proteomics data within models of hyperammonemia 10 mM ammonium acetate (AmAc) in differentiated murine C2C12 myotubes treated with AmAc for 3 and 24 h vs. untreated (UnT) controls (n = 3 biological replicates for each group; hyperammonemic and control mouse gastrocnemius muscle, n = 3–5 in each group) and vastus lateralis muscle from human control subjects or patients with cirrhosis and hyperammonemia (n = 4–6 each). All counts were used without restriction to demonstrate expression patterns. Figure S2. Multi‐omic analyses of feature extraction of components the sirtuin signaling pathway. Heatmaps of differentially expressed components of sirtuin signaling pathway. (a) Assay for transposase accessible chromatin sequencing (ATACseq), RNAseq, and proteomics in differentiated murine C2C12 myotubes treated with 10 mM ammonium acetate (AmAc) for 3 or 24 h compared to no treatment (UnT). (b) RNAseq from gastrocnemius muscle from mice treated with AmAc for 28 days compared to those treated with vehicle (phosphate‐buffered saline [PBS]). (c) RNAseq and proteomics from skeletal muscle from humans with cirrhosis (CIR) or healthy controls (CTL). Significance cutoffs for each heatmap (ATACseq p < 0.005 with fold change >∣1.5∣; RNAseq in C2C12 myotubes adjusted p < 0.05 Benjamini–Hochberg correction; all others unadjusted p < 0.05) are as shown in the panels. Experiments were performed in biological replicates of n = 3 in each group for myotube experiments; n = 4 in PBS and 5 in AmAc‐treated mouse experiments; n = 4 in each group for RNAseq and n = 6 in each group for proteomics in human skeletal muscle. Figure S3. Multi‐omic analyses of feature extraction fr [file ACEL-22-e13852-s004.pdf]
